# Mapping the distribution of packing topologies within protein interiors shows predominant preference for specific packing motifs

**DOI:** 10.1186/1471-2105-12-195

**Published:** 2011-05-24

**Authors:** Sankar Basu, Dhananjay Bhattacharyya, Rahul Banerjee

**Affiliations:** 1 Crystallography and Molecular Biology Division, Saha Institute of Nuclear Physics, 1/AF, Bidhannagar, Kolkata - 700 064, India; 2 Biophysics Division, Saha Institute of Nuclear Physics, 1/AF, Bidhannagar, Kolkata - 700 064, India

## Abstract

**Background:**

Mapping protein primary sequences to their three dimensional folds referred to as the 'second genetic code' remains an unsolved scientific problem. A crucial part of the problem concerns the geometrical specificity in side chain association leading to densely packed protein cores, a hallmark of correctly folded native structures. Thus, any model of packing within proteins should constitute an indispensable component of protein folding and design.

**Results:**

In this study an attempt has been made to find, characterize and classify recurring patterns in the packing of side chain atoms within a protein which sustains its native fold. The interaction of side chain atoms within the protein core has been represented as a contact network based on the surface complementarity and overlap between associating side chain surfaces. Some network topologies definitely appear to be preferred and they have been termed 'packing motifs', analogous to super secondary structures in proteins. Study of the distribution of these motifs reveals the ubiquitous presence of typical smaller graphs, which appear to get linked or coalesce to give larger graphs, reminiscent of the nucleation-condensation model in protein folding. One such frequently occurring motif, also envisaged as the unit of clustering, the three residue clique was invariably found in regions of dense packing. Finally, topological measures based on surface contact networks appeared to be effective in discriminating sequences native to a specific fold amongst a set of decoys.

**Conclusions:**

Out of innumerable topological possibilities, only a finite number of specific packing motifs are actually realized in proteins. This small number of motifs could serve as a basis set in the construction of larger networks. Of these, the triplet clique exhibits distinct preference both in terms of composition and geometry.

## Background

Despite several decades of arduous effort, mapping of protein primary sequences to their three dimensional folds, referred to as the second genetic code, remains an unsolved scientific problem. What appears to be lacking is a comprehensive theory, integrating two factors which definitely condition the isomorphism between sequence and fold, namely (1) the pattern of hydrophobicities embedded in the polypeptide chain [1] and (2) the packing of amino acid side chains to give densely packed [2] protein interiors. Under the present circumstances, the more tractable approach is the 'inverse protein folding problem' [3,4], that is to identify protein primary sequences [5] consistent with and supportive of a given fold, an idea which has found considerable application in the *de novo *design of targeted protein structures [6-9]. Yet even here, it was realized earlier on, that in *de novo *design, attainment of dense, well-packed protein cores (a hallmark of native, correctly folded proteins) was neither an automatic part of the design process nor acquired simply by chance [10,11]. Most often, it was observed (especially for longer sequences) that design led to molten globules or complete unraveling of the structure [12,13]. An instructive example was the repeated failure to design parallel (α/β)_8 _- TIM barrel [14,15], finally resolved successfully by Offredi *et al. *[16], where a term optimizing for side chain packing specificity was deliberately included in the computational process. In fact, one indicator for successful computational design [17-20] is the attainment of densely packed side chains in the interior of the targeted protein, experimentally characterized by the absence of ANS binding [16]. Thus, a comprehensive theory with regard to the packing of side chain atoms within proteins would not only provide insight into protein structures, facilitate their prediction [21] and would also be a valuable aid in the design of novel proteins.

Traditionally, there have been two models of protein packing: (1) the 'jigsaw puzzle' and (2) the 'nuts and bolts' model which lie on the opposite ends of the spectrum. The jigsaw puzzle model attributed to Crick [22], postulates the stereo specific interdigitation of amino acid side chains giving rise to densely packed protein interiors. On the other hand, the nuts and bolts model [23] does not require the association of side chains with specific geometry and asserts that the internal architecture of proteins arises simply due to the high compaction of side chain atoms within a constrained volume. Lately, another model referred to as the 'oil drop' model [24,25] has been proposed in order to capture the dynamic fluctuations in protein cores. Possibly, all these models concentrate exclusively on some special features of interior packing. However, using a surface complementarity function a previous report from this laboratory [26] demonstrated that binary association between two hydrophobic side chains (Leu-Leu, Leu-Phe etc), with high surface fit and maximal overlap between their corresponding residue surfaces, did exhibit specific inter-residue geometry. It was thus clear that at least for a subset of contacts (with high fit and overlap) predictions of the jigsaw puzzle model were indeed valid.

One drawback from all such studies is that the inter-residue interactions which sustain a native fold are more accurately modeled as a network rather than a discrete assortment of binary interacting pairs. Several attempts have been made to view protein structures as contact networks [27-35] wherein the amino acids have been designated as nodes and their mutual non covalent interactions as edges. The character of these networks (in terms of degree distribution, clustering coefficients, characteristic pathlength etc.) exhibit variability depending on the cutoffs used to define inter-atomic contact. By and large, most protein contact networks preserve 'small-world' character (local cohesiveness, global reach) [29,34,36-38] and display signatures of assortative mixing (preferential attachment of new nodes to pre-existing high degree nodes) [34]. However, degree distribution can be exponential, sigmoidal or dependent on a single exponent - as a function of the criteria used to define atomic interactions [32]. It has also been noted that in certain aspects protein contact networks differ significantly from other real world networks, for example in the restricted number of edges a node can have. Apart from providing insights into protein structures, these networks have been used to identify residues implicated in folding nuclei [35] and transition states [28], identifying functional residues involved in the active site [30], hubs stabilizing the packing of secondary structural elements [32], rationalization of the difference in protein stabilities from thermophilic/mesophilic organisms [32] and estimation of folding rates [27,31]. The utility of the network view of the protein structure is thus fairly well established.

In this study, the distribution of such networks from a database of protein structures has been analyzed in order to identify specific topological patterns in side chain association within protein cores. Such an analysis led to the recognition that certain packing topologies defined as packing motifs were found preferably in proteins. A limited region of the topological space was exhaustively mapped in terms of frequently occurring packing motifs, combinations of which could lead to networks of larger sizes. It was found that indeed larger networks could be assembled out of a basis set of smaller ones. One such frequently occurring motif namely the three residue clique received special attention with regard to its composition and geometry of associating residues.

Central to pursuing the research objectives outlined above was the extension of the jigsaw puzzle model into protein contact networks. Thus protein contact networks have been defined primarily in terms of surfaces rather than distance between point atoms (although such networks have also been studied in parallel for the sake of comparison). As mentioned previously, earlier studies [26] had established quantitative measures (in terms of surface complementarity and overlap) to identify those residue pairs whose interacting side chains exhibit specific geometry. These measures have now been used to define 'surface contact networks' based only on those inter-residue interactions which severely constrain geometry and thus could play a predominant role in stabilizing a particular fold.

## Results and Discussion

The primary objective of this study is to find, characterize and classify recurring patterns in the packing of side chain atoms within a protein which sustains its native fold. In this task we have deliberately chosen those contacts which strongly and specifically condition the inter-residue geometry of association. Since the majority of atomic contacts inside a protein are contributed by side chain atoms, it is natural to represent such interior packing as a network, defined primarily in terms of contact between their corresponding van der Waals surfaces (ASCN). In addition, point atom contact networks (APCN) have also been studied simultaneously (albeit with a fairly strong interaction cut off: 3.8 Å), by way of comparison.

Contact between any two surfaces can be characterized in terms of overlap (Ov) that is the extent to which two surfaces are conjoined and by their goodness-of-fit or surface complementarity (S_m_) **(see Methods, section: Surface Complementarity)**. In a previous work, it was demonstrated that when surface association between two amino acid side chains were greater than equal to 0.1 and 0.5 in Ov and S_m _respectively (defined on a Connolly surface), angular distributions specifying inter-residue geometry exhibited significant deviations from a random distribution [26]. For a corresponding van der Waals surface, the values of S_m _were found to be marginally lower for the same binary interactions. In contrast to point atoms, the definition of 'contact' **(see Methods, section: Surface Complementarity) **between two surfaces is not necessarily commutative (i.e. A contact B does not imply B contact A). In networks based on surface contact, nodes representing residues A and B have been connected with an edge only when (1) the contact between A and B is commutative and (2) their reciprocal S_m _and Ov both are greater than equal to 0.4 and 0.08 respectively. For strong association between two residue surfaces their contact is expected to be commutative, which also effectively simplifies the network to an undirected graph. For both point atom and surface contact networks, inter-atomic distance and surface-overlap bear a strong positive linear correlation. S_m _on the other hand appears to be an additional feature for the latter. Interestingly, the choice of 3.8 Å as the interaction cut off for point atoms led to maximum resemblance between the two categories of networks.

**Figure 1:**
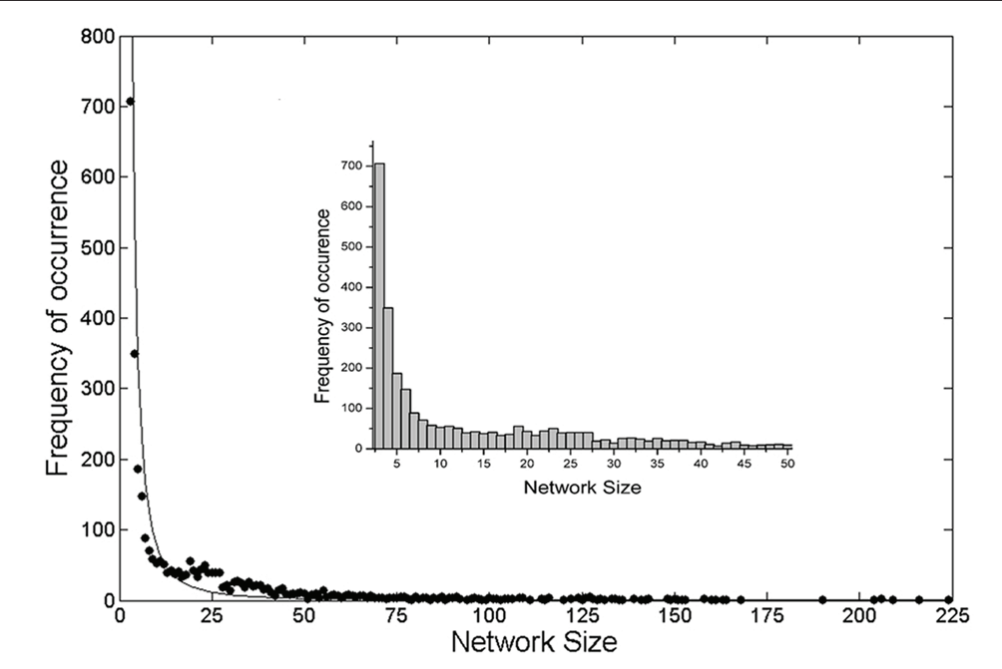
**Distribution of surface contact networks according to size**. Frequency distribution of networks of different sizes (n) for ASCN follows a power law decay (Corresponding histogram is displayed in the inset, the X axis being truncated at n = 50).

The first step was to study the distribution of networks in the protein database on the basis of size i.e. the number of constituent nodes. Networks of smaller size (3-10 nodes) dominated the distribution (Figure 1, additional file 1: Figure S1) with a rapid decay in frequency for larger networks (> 50 nodes). The distributions were however characterized by a long tail such that networks with greater than 200 nodes were also found, though with highly diminished frequency. The distributions for both point-atom and surface contact networks were very similar. The characteristic shape of the distribution could be adequately described by a power law (*f *(*x*) = *k.x*^-*n*^, where *x *is the network size), though, surface contact networks did exhibit some deviations in the range of graphs with 21 to 40 nodes (in terms of higher observed frequencies than expected from a power law). The exponent, *n *was found to be 2.2 and 2.1 for ASCN and APCN respectively. Relaxation of the cutoffs on S_m _and Ov did not appear to significantly alter the basic character of the distribution apart from decreasing the population for smaller networks thereby extending the tail for larger ones. On the other hand more stringent cut offs (S_m _> = 0.5, Ov > = 0.1) led to the disintegration of the larger graphs, consequently increasing the frequency of small (3-10 nodes) and medium (11-20) sized networks with a drastic curtailment in the number of larger graphs (highest network size obtained was 49 in comparison to 223 for S_m_> = 0.4, Ov > = 0.08) (Table 1). Thus, as has been previously observed [32], there appears to be a very narrow margin in terms of more stringent contact criteria which can abruptly change the spread and extent of contact networks within proteins.

**Table 1:** Frequency distribution of contact networks according to size

	Number of Networks					
	
Network Size	APCN	ASCN				
		
		(0.0, 0.0)	(0.2, 0.05)	(0.3, 0.07)	(0.4, 0.08)	(0.5, 0.1)
3	1168	58	162	336	**707**	1995

4	614	51	99	165	**349**	1016

5	433	29	57	99	**187**	641

6	273	28	33	62	**147**	452

7	198	11	32	49	**90**	314

8	148	6	28	34	**71**	230

9	125	12	16	29	**58**	195

10	99	4	17	27	**53**	134

11-20	564	32	78	152	**435**	476

21-30	236	40	119	224	**341**	47

31-40	130	72	107	153	**217**	8

41-50	72	50	60	104	**105**	4

51-100	165	241	243	228	**203**	-

101-150	60	127	122	99	**63**	-

151-200	33	99	88	69	**11**	-

201-250	10	70	51	29	**6**	-

251-300	-	26	19	8	-	

301-350	-	4	1	-	-	

Number of networks found in the database for different ranges of network size (i.e., number of constituent nodes). Cutoffs in surface complementarity (S_m_) and overlap (Ov) respectively are given in bold within parentheses (for ASCN). Results for the chosen set of cutoff values, used in the analysis (0.4, 0.08) are highlighted in bold.

The same calculations repeated for polypeptide chains distributed in bins with 75-150, 151-300, 301-500 residues gave similar curves, though for bins of larger chain length, networks of larger size appeared, thereby extending the long tail of the distribution. As expected, frequency distributions of polypeptide chains containing networks of a particular size gave a similar decaying trend with increasing network size; that is networks of smaller size were found embedded in polypeptide chains regardless of the chain length, whereas instances of larger graphs were progressively rare. These distributions tend to indicate that (in the subset of contacts with specific inter-residue geometry of association) small (3-10 nodes) to medium (11-20 nodes) sized networks are found universally in all protein structures, whereas linkage and/or fusion of these smaller networks to form larger ones is protein specific and is thus context dependent. Very large networks (> 150 nodes) were found only in 17 proteins (additional file 2: Table S1) almost all of which had chain length exceeding 400 residues with close packing between extended secondary structural elements (helices and sheets). Overall, most of the very large networks were found in α|β proteins (additional file 3: Table S2).

In a protein contact network, there is an obvious upper bound to the highest possible degree a node can have (dependent on the contact criteria) due to the limited volume of the residues involved in packing. For the present set of criteria, the highest degree of a node was found to be restricted to 8 and 9 for ASCN and APCN respectively.

**Figure 2:**
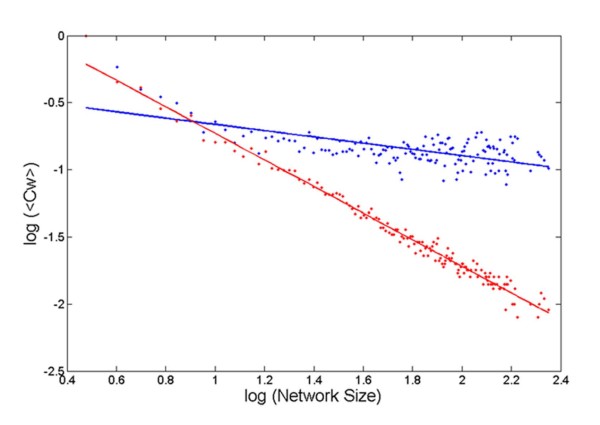
**Protein contact networks are locally cohesive**. Weighted average clustering coefficients (<Cw>) of contact networks for ASCN (blue) with their corresponding values for random networks (<Cr>: red) plotted against network size in a log-log scale.

From a graph theory perspective, local cohesiveness (or clustering) of side chains should lead to dense packing within protein cores. To test this proposition, average unweighted (C) and weighted (Cw) clustering coefficients were calculated for contact networks of all sizes. Both these coefficients gave rise to almost identical measures. In parallel, a statistically significant number of random graphs (of corresponding sizes) were generated **(see Methods, section: Deviation from random topology) **for the direct calculation of their clustering coefficients. In a log-log plot (Figure 2), average clustering coefficients of the contact networks decayed much less rapidly with increasing network size compared to corresponding random graphs. Since, these coefficients essentially determine the cliquishness of a typical neighborhood [39] in terms of clustering of local triplets [40], it follows that (closed) triplet cliques serve as the units of (non-zero) clustering. In other words, a graph of whatever size or connectivity will result in zero clustering (C = Cw = 0) if there is no 'closed triplet' found embedded within it. It is to be noted that any higher order clique could be considered as an association of nested triplet cliques. Since, these results confirm the probability of finding closed triplets to be much higher in proteins than random graphs, 3-cliques as 'clustering units' have thus received detailed attention in terms of geometry and composition, to be discussed in later sections.

### Packing Motif

One of the central concepts formulated in this study is that of a 'packing motif'. To start with, a packing motif is defined as a graph with a limited number of nodes (3-7), consisting of unique topological connections, which can be found either in isolation or can appear as a component or an induced subgraph, embedded within a larger graph. It follows that no two distinct motifs are super-imposable onto each other. In other words two motifs are identical (or topologically isomorphic) if there exists a one-to-one correspondence between their vertex sets which preserve adjacency [41]. The same motif can be found in different proteins and since a node (in the motif) does not conventionally represent any particular amino acid, it could stand for different sets of residues associated with diverse inter-residue geometries in the actual three dimensional assemblies. Thus a packing motif is a reduced representation of three dimensional residue clusters, rather analogous to super-secondary structural motifs where, for example, different combination of residues in unrelated proteins can fold into (say) a helix-turn-helix.

**Figure 3:**
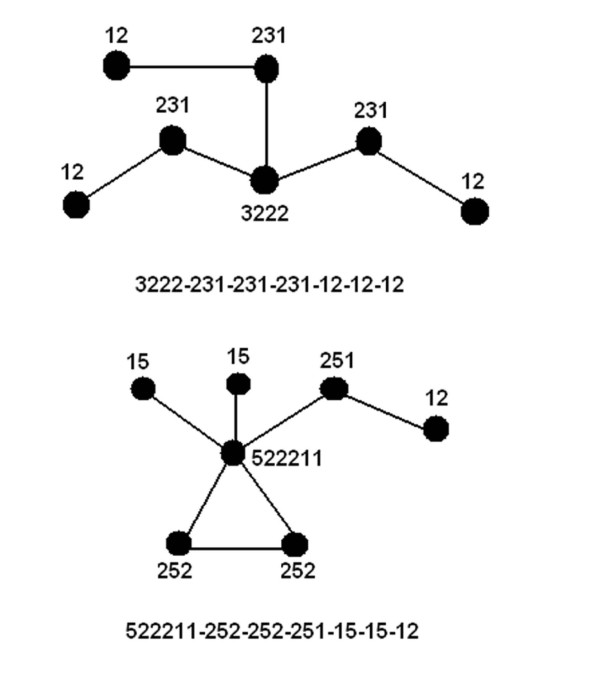
**A novel numerical scheme to identify graphs with unique topology**. Graphs (packing motifs) along with a unique number-string (motif identifier) displayed below each motif. Each node is assigned a concatenated numeric where the first digit stands for its own degree followed by degrees of its immediate neighbor sorted in a descending order.

In order to aid numerical manipulations, each motif was uniquely represented by a linear array of numbers (motif identifier) which can be regarded as a *complete set of invariants *[41] between any two isomorphic graphs. Initially each node of a given motif was assigned a string of numbers (of length *(d+1) *where *d *is its degree) starting with its own degree; followed by the degrees of its direct neighbors sorted in descending order. These numeric strings were collected as elements of an array and further sorted in descending order. Finally these sorted strings were concatenated, separated by a delimiter (Figure 3). This identifier-string representation of each motif facilitated the computational detection, classification and clustering of motifs from the protein database.

One of the primary objectives of this study is to (1) identify recurrent motifs in smaller graphs and (2) to ascertain whether larger graphs can be constituted by an assembly of suitable motifs with appropriate topological connections. Firstly, all contact networks observed in the protein database were sorted according to their size (n). For smaller graphs ranging from 3-7 nodes (or may be up to 10), each set (with nodes n = 3, 4 ... etc) is expected to populate a limited number of motifs (Table 2). Thus a motif could essentially be viewed as a prototype, while the actual networks observed in proteins as members belonging to a specific type of motif. To estimate the maximum number of possible motifs in networks of a given size (n), a series of random graphs were generated, conditioned by the fact that all nodes had to be connected to at least one other node in the graph **(see Methods, section: Algorithm to construct networks)**. In a protein contact network, there is a definite upper bound to the maximum number of edges a node can have. Therefore the highest degree (for a given network size, n) was determined from the set of actual protein contact networks, and this number was used to constrain the maximum attainable degree for the corresponding random graphs. Good agreement between the actual number of motifs observed in the database and the possible number of motifs from random graphs (with no cutoffs on the maximum attainable degree of a node) were found for n = 3, 4 with rapidly increasing divergence for n ≥ 5 (additional file 4: Table S3). Most probably this was due to systematic overestimation in the number of unique random graphs. Thus for graphs with n ranging from 7 to 10, the number of possible motifs were recalculated by varying the maximum allowable degree from 4 (for smaller side chains) to the highest observed value in corresponding protein contact networks, which happened to be either 6 or 7 (for bulkier residues). However despite lowering the cutoff on the permissible number of edges for a node it appeared that for n ≥ 5 a diminishing number of possible motifs is actually being realized within proteins (additional file 4: Table S3).

**Table 2:** Frequency distributions of small (3-10 nodes) networks with their corresponding number of motifs

	ASCN		APCN	
	
Network Size	Networks	Motifs	Networks	Motifs
3	707	2	1168	2

4	349	5	614	5

5	187	12	433	13

6	147	28	273	37

7	90	47	198	60

8	71	55	148	76

9	58	46	125	93

10	53	51	99	91

For a given network size, the number of networks observed in the database and the corresponding number of unique motifs have been tabulated. For example, 12 motifs were observed for 187 networks (ASCN) constituted of 5 nodes (3^rd ^entry of the table).

All networks were systematically searched for size of the maximal clique (n_c_) **(see Methods, section: Cliquishness) **which interestingly was found to be no more than 4 for embedded cliques (n_c _being 3 for a large majority of cases) and not exceeding 3 for complete graphs (or isolated cliques). In fact, the number of networks with a maximal clique of 3 and 4 nodes respectively, were found to be 1548 and 77 in case of ASCN (1662 and 146 in the same order: APCN). Since an n-clique should exactly have (^n^C_2 _- n) diagonal edges, it follows that any possible closed-ring topology of n > 4 to be found in the database can have at the most (^n^C_2 _- n -1) diagonal edges. Thus the possible network architectures spanning the space under study is expected to be restricted to a few basic topologies namely linear chains, closed triplets (with or without branching), closed quadruplets (including embedded 4-cliques), higher order ring closures (n > 4) with a restricted number of diagonal edges and possibly a series of non-planar graphs.

For n = 3 there were trivially only two possible motifs (1) the open linear chain (motif id: 211-12-12) and (2) the isolated closed triplet clique (motif id: 222-222-222). Both possibilities were found in protein contact networks, though with considerable difference in the number of their respective occurrences. The overwhelming majority of these three-node graphs are found to be open linear chains (660: ASCN; 1070: APCN) which offer greater flexibility unlike isolated closed triplet cliques (47: ASCN; 98: APCN) which can only occur, satisfying additional geometric constraints. It could also be possible that triplet cliques once formed display an inherent tendency to evolve into larger networks given the fact that a significantly larger number of these cliques were found to be embedded as induced subgraphs in larger graphs (8876: ASCN; 9102: APCN) relative to isolated instances. Out of a total of 719 polypeptide chains in the database, embedded triplet cliques were found at least once in 696, 689 for ASCN and APCN respectively whereas for isolated instances the corresponding numbers were 47 (ASCN) and 90 (APCN).

It is a relatively simple task (at least up to n = 5) to enumerate the possible number of motifs and then find their respective number of members (or the frequency of their occurrence) in the protein database. It is however a more complex exercise to propose a sound classification scheme, which leads to the regular ordering of actually observed motifs. To this end two additional concepts were defined namely family and path. Two motifs g(n) and g'(n+1) (with n and n+1 nodes respectively) are related by a path if the motif g'(n+1) can be formed from g(n) such that the node added to g(n) is linked to only one pre-existing node by a single edge. In other words the transformation g(n) → g'(n+1) is a path provided the newly added node has degree of one and the degree of one and only one pre-existing node in g(n) increases by one. Again, all motifs which can be linked by successive paths: g(n) → g'(n+1) → g''(n+2) ... etc. fall within the same family. However, in case the intermediate g'(n+1) was missing, g''(n+2) was still retained in the same family. Thus, essentially a path leads to linear branching(s) about nodes belonging to a basic core topology. It follows that a motif of larger size (greater than 7 nodes) can either belong to an already existing family provided it is appropriately linked by a path or belong to an entirely new family (for example, ring closures of n > 7) which was found to be remarkably less frequent.

**Figure 4:**
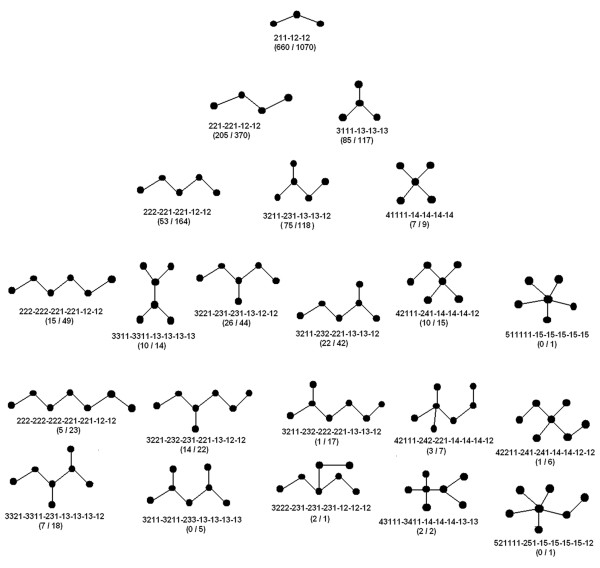
**Motifs belonging to family f1**. Network diagrams of motifs up to size 7 (nodes) belonging to family f1. The family describes topologies of minimally connected open linear chains. Motif identifier for each motif is displayed below the motif with the number of members for ASCN and APCN respectively in parentheses separated by a front slash.

**Figure 5:**
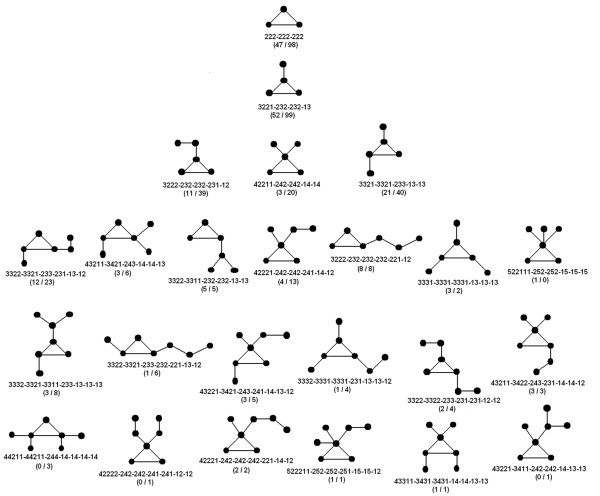
**Motifs belonging to family f2**. Network diagrams of motifs up to size 7 (nodes) belonging to family f2. The family describes topologies of triplet cliques with or without linear branching. Motif identifier for each motif is displayed below the motif with the number of members for ASCN and APCN respectively in parentheses separated by a front slash.

For n = 4, there are six possible motifs, of which five (with the sole exception of isolated quadruplet cliques or complete graphs of 4 nodes) were found to have members. Two motifs (221-221-12-12 and 3111-13-13-13) found to have the highest number of members (205, 85: ASCN and 370, 117: APCN respectively) could be related by a path to open linear chains (n = 3) or family: f1 (Figure 4). A third motif (3221-232-232-13 with 52 members in ASCN and 99 in APCN) was included in the family: f2, originating from closed triplet cliques (Figure 5). The remaining two (222-222-222-222 and 3322-3322-233-233) motifs were closed four membered rings (the latter having one diagonal edge) and were put in distinct families (f3a, f3b) (additional file 5: Figure S2). Thus up to n = 4, a total of 7 motifs with a total number of 1056 (ASCN) and 1782 (APCN) members were organized into 4 families (f1, f2, f3a, f3b), with the overwhelming majority (1049: ASCN and 1754: APCN) of members incorporated into families f1 and f2.

More or less the same trend was preserved for n = 5 where new motifs with significant number of members were again placed in the families f1 and f2. Additional motifs with marginal membership were included in f3a and f3b, which were essentially branched four membered rings. Two more families (f4a and f4b) were created at this point, the former (f4a) originating from the closed pentagon (with no diagonals) whereas the latter (f4b) includes the pentagon with a single diagonal edge (additional file 6: Figure S3). Other families at this point include topologies demonstrated by two or more closed triplets; fused along their edges (f5) (additional file 7: Figure S4), connected at a node (f6a) or connected by an edge (f6b) (additional file 8: Figure S5). Once again, families other than f1 and f2 exhibited negligible memberships. Moving up levels n = 6, 7 led to the inclusion of only five more families: (a) linkage of two four membered rings through a node (f7: 1 member each in ASCN and APCN) (additional file 8: Figure S5), (b) embedded quadruplet cliques with additional linear branching (f8a: 1 in ASCN and 4 in APCN) (c) non-planer graphs excluding quadruplet cliques (f8b: 1 in ASCN and 3 in APCN) (additional file 9: Figure S6), (d) closed six membered ring with or without diagonals edges (f4c: 3 each in ASCN and APCN) (additional file 6: Figure S3) and (e) graphs where two non-adjacent nodes are connected by more than two sequences of successively connected nodes (f8c: 4 each in ASCN and APCN) (additional file 9: Figure S6). The addition of nodes from n = 5 to n = 6, 7 primarily led to the addition of motifs in the pre-existing families by, (1) increasing the length and branching of the linear chain (f1), (2) increased linear branching about the triplet cliques (f2), (3) progressive branching and inclusion of diagonal edges of the higher order closed rings (f3a, f3b, f4a, f4b, f4c, f5).

At this stage it became obvious that the initial definitions were leading to a proliferation of families with almost negligible membership. Thus to reduce the number of such families some exceptions were made. For families originating from five membered rings (f4a & f4b), motifs with a closed triplet fused about any two vertices of the pre-existing pentagon (3332-3322-3321-233-232-232-13: f4a & 43322-3432-3432-243-242-232, 533221-3532-3532-253-252-232-15, 44322-44321-3442-244-242-232-12: f4b) (additional file 6: **Figure S3) **were included in the same respective families. Finally up to n = 7, 94 (ASCN) and 117 (APCN) motifs with 1480 (ASCN) and 2686 (APCN) members respectively were organized into 13 families.

The same procedure described above was performed separately for polypeptide chains in each individual protein class (all α, all β, α|β, α+β), in order to investigate any preference for specific motifs or families. By and large no outstanding preference was observed (after suitably normalizing for the number of polypeptide chains in each class) **(see Methods, section: propensity)**, though a somewhat reduced propensity was found for family f2 (originating from closed triplet cliques) in the case of all α proteins (0.67) with a relative increase in propensity for α|β (1.20). The statistics was not robust for most families barring f1 and f2 due to their extremely low frequency of occurrence (additional file 10: Table S4).

The trend regarding the distributions of motifs (with preference for families f1 and f2) was not radically changed for different cutoffs on S_m _and Ov, other than the reduction of smaller (isolated) motifs on gradually relaxing the cutoffs. Notably, the population of f2 (family of closed triplet cliques) was found to gradually approach that of f1 (family of open linear chains) upon systematic lowering of the cutoffs, allowing weaker links to close the cluster. On the other hand, the application of more stringent cutoffs (S_m _> = 0.5, Ov > = 0.1) led to an increase in the population of smaller motifs, predominantly in the f1 family. Most notable was the increase in frequencies of motifs with 7 nodes (f1) probably due to the exclusion of a few weaker links leading to 'minimally connected' open linear chains (additional file 11: Table S5).

Since from n = 5 to n = 6, 7 a diminishing number of families (only 5) are added with negligible membership, it is highly likely that larger networks (n > 10) will generate motifs either populating already existing families or will be assembled by joining pre-existing motifs following a defined set of rules. Since the same trend of preferential membership in the first two families were followed in networks of size n = 8, 9, 10 **(data not shown) **it was decided to begin the construction of higher order graphs out of a motif basis set obtained from networks of sizes up to n = 7, with the understanding that motifs of sizes greater than 7 nodes (located in appropriate families) would also be utilized depending on the context of a particular network. Variants of a motif with branching(s) from nodes different from those originally observed (especially for closed ring topologies) though preserving core topology would also be used in the resolution of larger graphs into subgraphs. For n = 10, the total number of motifs became comparable to the number of networks or members (Table 2). Thus, the resolution of larger graphs in terms of the proposed basis set was attempted for n greater than 10.

**Figure 6:**
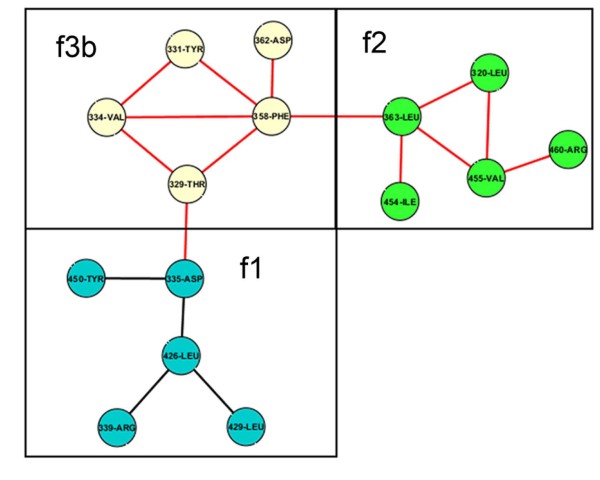
**A contact network resolved into components**. A contact network of size 15 (from 1OWL.pdb) resolved into isolated components (separated by boxes) belonging to families f1, f2 and f3b.

**Figure 7:**
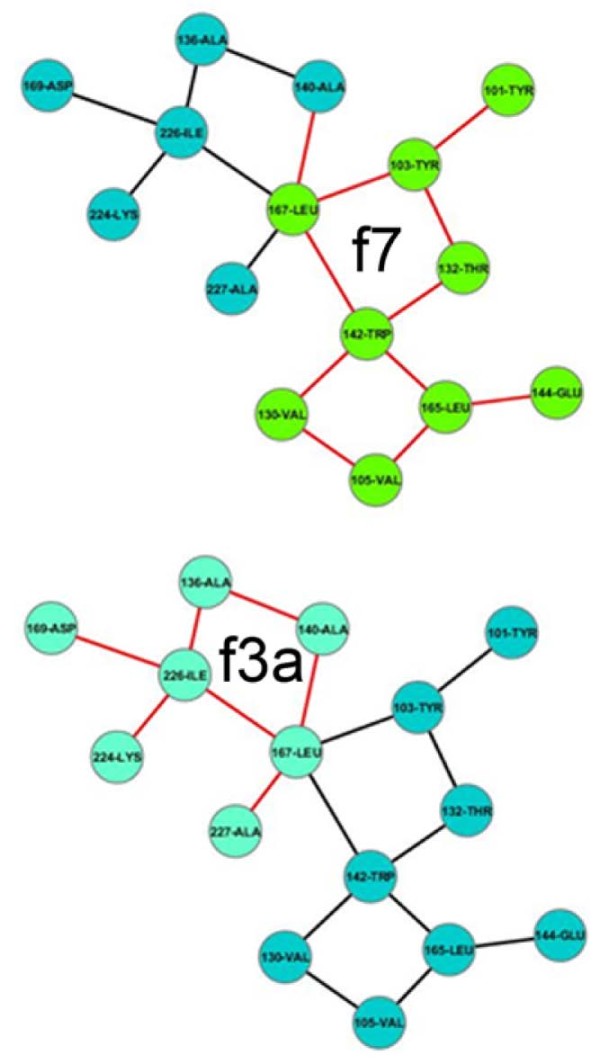
**A contact network resolved into induced subgraphs**. A contact network of size 15 (from 2HNF.pdb) resolved into induced subgraphs (highlighted by different colors) belonging to families f3a and f7.

**Figure 8:**
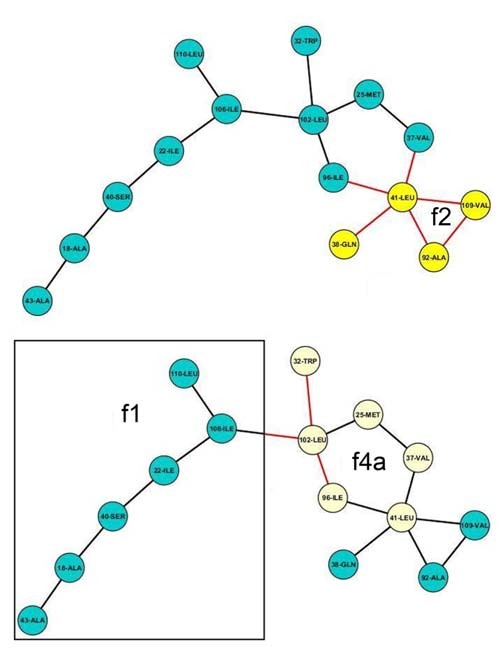
**A contact network resolved into induced subgraphs and components**. A contact network of size 15 (from 1MQV.pdb) resolved into induced subgraphs (highlighted by different colors) and components (separated by boxes) belonging to families f2, f4a and f1 respectively.

Generally, a graph can be resolved into either a degenerate subset of spanning subgraphs (derived by deleting edges of a graph such that the number of nodes remains conserved) and/or induced subgraphs (by deleting nodes with their incident edges such that two nodes adjacent in the subgraph must be adjacent in the original graph) [41,42]. Thus, analogous to a spanning subset, deleting a judiciously chosen set of specific edges of a graph should produce independent unconnected components. Since, in this study, such isolated components are treated as graphs **(see Methods, section: Algorithm to construct networks) **it should be possible to resolve a larger graph into a set of motifs (regarded as components) or their variants from pre-existing families, by deleting specific edges. Such edges, however, strictly exclude those being involved in a closed ring (of any size, n > = 3), so that the method does not trivially produce an arbitrary combination of motifs. On the other hand, in an induced subgraph, there exists an identical topological relation between any two corresponding nodes to that of the original graph. This one-to-one mapping serves as the basis for a computational search for motifs embedded as induced subgraphs in a larger graph. These two fundamental concepts of graph-analysis were successfully implemented to test the hypothesis whether the motif space is by and large adequate in assembling larger graphs. Contact networks for n = 15 (38: ASCN; 47: APCN) were carefully examined using Cytoscape [43] (additional file 12: Dataset S1) and it was found that the majority of (24: ASCN; 28: APCN) networks could be resolved across one or more edges to produce isolated components which were invariably motifs belonging to pre-existing families (Figure 6). Other networks could not be resolved into pre-existing motifs by simply cutting across edges and in such instances the majority of possible induced subgraphs embedded in the graph were recognized as pre-existing in the motif basis set (Figure 7). Cases were also found where a larger graph was resolved into both components and induced subgraphs (10: ASCN, 15: APCN) (Figure 8). It is to be noted that there can be more than one sequence of steps to assemble a graph from degenerate sets of subgraphs following either of these procedures. As expected, for all cases, newly emerging motifs were restricted only to ring closures of greater than 6 nodes. Thus, predictably, for graphs with more than 15 nodes, new motifs should mostly be closed ring topologies with increasing number of nodes in the ring.

Possibly, introduction of cofactors and metals into proteins could distort contact networks to give rise to novel topological architectures. Preliminary analyses of networks associated with metal binding sites (on a reduced database consisting of 63 polypeptide chains) exhibit novel 'highly connected' motifs not found in ASCN/APCN which show a greater tendency to form higher order cliques (n_c _= 5, 6) provided the metal(s) is also included in the contact network.

### Triplet Clique

The classification of motifs into families reveals that the overwhelming majority of contact networks found in protein structures occur in the first two families (f1 + f2) originating from core topologies of either open linear chains or closed triplet cliques. Although the simple rule governing the classification of motifs leads to about thirteen families in all, a significant proportion of these families have such negligible membership that they can be currently disregarded. To investigate whether the most frequently occurring motifs exhibit any preference in their constituent amino acid residues and whether their side chains pack with specific geometry, closed triplet clique (regarded as the 'clustering unit') was chosen for further investigation. Analysis of the relative frequencies of isolated and embedded triplet cliques appeared to suggest that isolated cliques (or in other words, complete graphs of three nodes) have an inbuilt tendency for further branching(s) about the three constituent nodes resulting in their being embedded in larger graphs. Thus, to improve the statistics, both isolated triplet cliques and those embedded as induced subgraphs in larger graphs were pooled together. Further, since hydrophobic residues show greater propensity for burial and inclusion into contact networks, only the subspace of triplet cliques composed exclusively of hydrophobic residues (Ala, Val, Leu, Ile, Phe, Tyr, Trp) were considered. The resultant number of triplet cliques thus reduced to 4874, 1545 out of a total of 8923, 9200 for ASCN and APCN respectively. Interestingly, the number of such cliques was found to be significantly higher for ASCN relative to APCN, and since surface contact networks have been defined with a view to identify side chain associations with specific inter-residue geometry, results from ASCN alone are being discussed, which in any case should give superior statistics.

**Figure 9:**
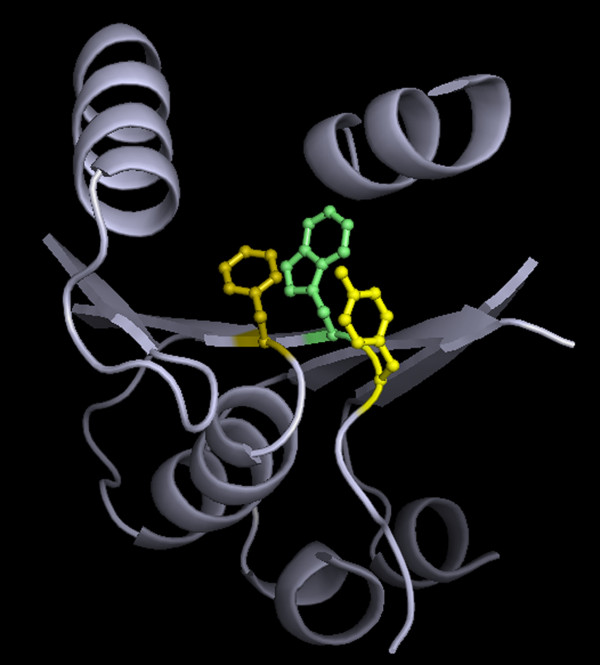
**A three-residue clique, embedded in the protein interior**. An embedded triplet clique (from 3F67.pdb) constituted of 119-Phe (Olive), 142-Trp (Lime) and 143-Tyr (Yellow) displayed as sticks in a background of broken stretches of the backbone being displayed as cartoon (Cyan). The image was constructed using PyMol [44].

For a combination of three residues packed in the form of a closed triplet clique (Figure 9) three possibilities can be expected: (i) **C1**: all the three constituent residues are non-identical (e.g., Phe-Leu-Val) (ii) **C2**: one residue unique, the other two being identical (e.g., Ala-Ala-Leu) and (iii) **C3**: all three residues identical (Leu-Leu-Leu). Starting with the set of seven hydrophobic residues (listed above) the total number of possible combinations for each case are 35, 42 and 7 respectively. For assemblies of three identical residues (C3), the highest frequencies were observed for Leu-Leu-Leu (55.5%), followed by Ile-Ile-Ile (~19.2%) and an almost equal proportion for Phe-Phe-Phe and Val-Val-Val (both ~12%) (additional File 13: Table S6). A negligible fraction of triplets was found to be composed exclusively of Ala, Trp and Tyr. In all probability an assembly of three leucines provides optimal conditions, in terms of shape and size for cohesive packing. Ala and Trp represent the opposite ends of the spectrum with regard to volume and the association of tyrosines could be disfavored due to the partial charge of its terminal side chain oxygen (OH). A similar trend was observed for triplet cliques with one unique and two identical residues (C2). 40% of all triplets in this category were composed of two leucines, with X-Ile-Ile, X-Phe-Phe and X-Val-Val exhibiting frequencies 20.2%, 16.8% and 16.7% respectively. Predictably, X-Ala-Ala, X-Trp-Trp and X-Tyr-Tyr were rarely found. For hydrophobic clusters with three non-identical residues (C1), the most frequent composition was that of Ile-Leu-Val (~15.4%). It is notable that the most frequent triplet clique in this category can also be considered to be an exception as the overwhelming majority of triplets consist of at least one aromatic residue (Trp, Tyr or Phe: 79.2%). Even here, occurrence of only one aromatic in the triplet clique appears to be preferred over two, whereas cliques composed exclusively of aromatics seldom occur.

Thus, the data indicates that even though most of the possible residue combinations are realized in local closed triplets within proteins, there is a wide divergence in their respective frequencies. Some residue combinations definitely appear to be preferred over others. Moreover, since only a subspace has been studied, the compositional propensities appear to be fairly pronounced, rather than outstanding. Without the use of surfaces and careful classification of triplet cliques (based on their compositions) these could well be overlooked. The overall pattern in composition remained fairly unmodified upon changing cutoffs in S_m _and Ov. Even then, the formation of well packed three residue cliques in proteins appears to be constrained in terms of the total volume occupied by the triplet and probably their inter-residue geometry. In all probability, only some residue combinations optimally satisfy these constraints. The question then is what are the geometrical constraints imposed on these three-residue cliques?

**Figure 10:**
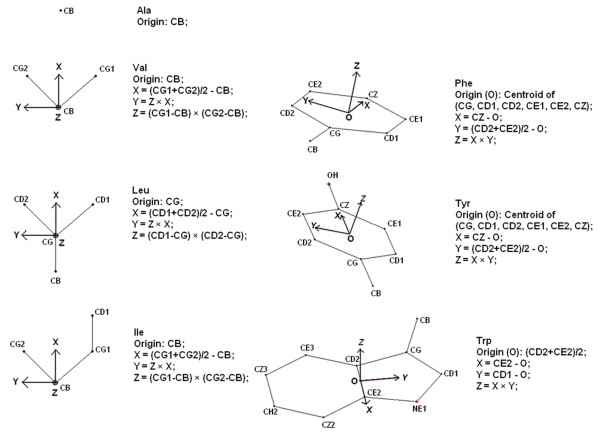
**Internal frames defined on individual residues**. Internal (right handed) frames of reference for the amino acid residues defined on the side chain atoms.

**Figure 11:**
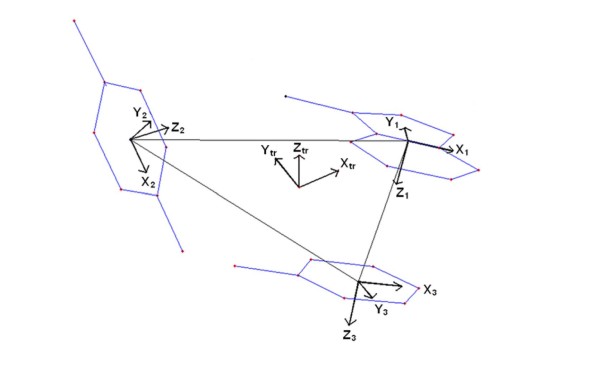
**Global frame of reference defined on the triangle, based on a three residue clique**. The triangle formed by joining the origins of the three internal frames of references (**X**_**1**_**, Y**_**1**_**, Z**_**1**_**; X**_**2**_**, Y**_**2**_**, Z**_**2**_**; X**_**3**_**, Y**_**3**_**, Z**_**3**_) defined on the residues, constituting the triplet clique. The global frame of reference (**X**_**tr**_**, Y**_**tr**_**, Z**_**tr**_) defined on the triangle, is also displayed.

Extending the methodology established by Thornton and Singh [45] (to study inter-residue geometry between two amino acids) an internal right-handed Cartesian frame of reference was defined (Figure 10) for each of the three residues constituting the triplet clique. Connecting the origins of the three internal frames of reference constructs a triangle, which can be considered to be a reduced geometric representation of the assembly. A global frame of reference was then defined on the triangle plane **(see Methods, section: Geometry of three-node packing motifs)**, the global Z axis being the normal to the plane and the origin being set at the centroid of the triangle (Figure 11). However, there can always be two degenerate directions of the normal (Z axis). Therefore in order to secure uniformity among all the reference frames the following conventions were adopted:

1. For **C1 **(all three residues different), the three residues (R1, R2, R3) were first sorted on the basis of their side chain volume R1 > R2 > R3. Let the vector directed from the origin of R1 to R2 be **v1 **and that from R1 to R3 be **v2**. Then the global Z axis was defined as **v1 **× **v2 **and the global X axis as the unit vector directed from the global origin towards the origin of R1.

2. For a given composition in **C2 **(e.g., Leu-Phe-Phe) one specific example was arbitrarily chosen whose unique residue was designated R1 and R2, R3 were assigned such that the identical procedure outlined above (in 1) resulted in an acute angle being subtended between the global Z and the internal Z of R1. All other triangles with the same composition were superposed onto this template. The calculations were repeated starting from different templates to confirm that the results were not artifacts of this geometrical procedure.

3. In case of **C3 **(e.g., Leu-Leu-Leu), a randomly chosen triplet was arbitrarily assigned R1, R2, R3 and the global frame was defined following procedure (1). All other triangles of the same composition were superposed onto this template. To select for the best possible superposition (additional file 14: Figure S7) in each case 6 combinatorial possibilities were checked. Similar to C2, the calculation was repeated for different starting templates.

For almost all the compositions the lengths of the triangular edges and the internal angles were severely constrained, with standard deviations ranging from ~ 0.5 - 0.6 Å and ~ 5 - 10° in lengths and angles respectively. In almost all the cases, the triangle approximates to being equilateral with the average of all the three sides lying between 5-6 Å and angles close to 60° (± 10°). Inclusion of bulky residues in the triplet cliques (Tyr-Phe-Leu), (Tyr-Phe-Ile) did not appear to significantly alter the overall trends observed in these triangular parameters. The longest average lengths were observed for Ile-Leu-Leu (6.3 ± 0.6), Leu-Ile-Ile (6.4 ± 0.5), Ile-Val-Val (6.3 ± 0.5) and Ile-Phe-Phe (6.3 ± 0.6) (Table 3).

**Table 3:** The triangle constructed from the associated residues in a triplet clique approximates to being equilateral

Composition			Frequency	**<r**_**12**_**>**	**<r**_**13**_**>**	**<r**_**23**_**>**	**< Ω**_**1**_**>**	**< Ω**_**2**_**>**	**< Ω**_**3**_**>**
ILE	LEU	VAL	322	5.9 (0.7)	5.9 (0.7)	5.6 (0.5)	56.5 (7.5)	61.6 (9.2)	61.9 (8.6)

ILE	LEU	LEU	291	6.3 (0.6)	5.6 (0.6)	5.5 (0.6)	55.0 (7.9)	55.9 (5.9)	69.1 (7.1)

PHE	ILE	LEU	276	5.8 (0.8)	5.4 (0.6)	5.9 (0.6)	63.2 (9.5)	55.5 (9.3)	61.3 (10.9)

VAL	LEU	LEU	268	5.4 (0.5)	5.9 (0.4)	5.6 (0.6)	59.2(7.8)	65.2 (5.6)	55.5 (5.4)

PHE	LEU	VAL	246	5.5 (0.6)	5.3 (0.6)	5.6 (0.6)	61.6 (8.2)	57.7 (9.4)	60.7 (9.4)

PHE	LEU	LEU	237	5.1 (0.5)	5.8 (0.5)	5.6 (0.5)	62.1 (8.4)	65.7 (7.6)	52.1 (5.7)

LEU	LEU	LEU	202	5.2 (0.5)	6.1 (0.3)	5.7 (0.4)	59.5 (4.2)	67.8 (4.9)	52.6 (4.7)

LEU	ILE	ILE	187	6.4 (0.5)	5.7 (0.6)	6.3 (0.8)	63.4 (11.7)	52.8 (7.0)	63.8 (7.6)

LEU	PHE	PHE	162	5.1 (0.5)	5.8 (0.5)	5.5 (0.5)	60.7 (9.3)	66.1 (7.0)	53.3 (5.9)

TYR	ILE	LEU	151	5.7 (0.8)	5.3 (0.6)	6.0 (0.6)	65.5 (8.8)	53.9 (9.5)	60.5 (11.1)

PHE	ILE	VAL	150	5.7 (0.7)	5.4 (0.7)	5.9 (0.7)	64.9 (10.6)	55.6 (10.7)	59.5 (10.3)

VAL	ILE	ILE	134	5.5 (0.6)	6.3 (0.6)	6.2 (0.8)	63.1 (11.1)	64.9 (7.5)	52.0 (7.2)

LEU	VAL	VAL	128	5.9 (0.4)	5.3 (0.5)	5.7 (0.5)	61.1 (8.0)	54.8 (4.7)	64.1 (6.1)

ILE	VAL	VAL	119	6.3 (0.5)	5.5 (0.6)	5.7 (0.5)	57.8 (8.1)	54.4 (6.4)	67.8 (5.9)

TYR	PHE	LEU	117	5.5 (0.6)	5.4 (0.6)	5.4 (0.5)	59.6 (9.0)	59.6 (10.1)	60.7 (9.5)

ILE	PHE	PHE	105	6.3 (0.6)	5.5 (0.7)	5.5 (0.6)	54.6 (8.5)	54.9 (7.3)	70.5 (7.2)

TYR	LEU	LEU	104	4.9 (0.4)	5.9 (0.5)	5.5 (0.6)	60.9 (8.5)	67.9 (7.1)	51.2 (6.3)

TYR	LEU	VAL	98	5.4 (0.6)	5.4 (0.7)	5.5 (0.4)	60.7 (7.6)	59.4 (10.1)	59.9 (10.4)

PHE	ILE	ILE	94	5.5 (0.6)	6.3 (0.7)	6.4 (0.9)	66.0 (11.3)	63.1 (8.5)	50.8 (6.7)

PHE	VAL	VAL	88	4.9 (0.6)	5.8 (0.5)	5.7 (0.5)	63.5 (10.0)	65.7 (7.7)	50.8 (6.8)

TYR	PHE	ILE	85	5.6 (0.5)	5.7 (0.9)	5.8 (0.8)	61.8 (12.1)	59.9 (13.3)	58.2 (9.9)

TYR	PHE	VAL	77	5.5 (0.6)	5.5 (0.6)	5.5 (0.7)	59.9 (10.7)	59.3 (10.0)	60.7 (9.9)

ILE	ILE	ILE	70	5.5 (0.6)	6.4 (0.5)	7.0 (0.6)	72.1 (6.2)	59.9 (4.5)	48.0 (5.8)

TYR	ILE	VAL	69	5.8 (0.9)	5.4 (0.6)	5.9 (0.7)	63.4 (10.6)	55.0 (10.3)	61.5 (11.7)

VAL	PHE	PHE	67	5.8 (0.5)	5.0 (0.5)	5.4 (0.5)	59.8 (9.0)	52.3 (6.9)	67.8 (8.0)

TRP	PHE	LEU	56	5.5 (0.6)	5.6 (0.6)	5.4 (0.6)	58.7 (8.5)	61.6 (10.3)	59.7 (9.3)

TYR	PHE	PHE	53	5.9 (0.5)	5.1 (0.4)	5.5 (0.5)	59.8 (9.2)	52.6 (5.7)	67.5 (8.1)

LEU	VAL	ALA	50	5.7 (0.5)	4.7 (0.4)	4.7 (0.4)	53.4 (5.3)	53.1 (6.8)	73.5 (7.7)

Average lengths of sides (Å) and internal angles (°) (along with their standard deviations in parentheses) of the triangle formed by joining the origins of the three internal frames corresponding to the constituent residues (in a triplet clique) are tabulated. Only those compositions have been given whose frequencies are greater than equal to 50.

Relative geometries of the three constituent residues from the perspective of the abstract triangle defined above were analyzed by means of two more angles, namely tilt and swivel. Dot product of the global Z axis defined on the triangle plane with Z axes (Z_1_, Z_2_, Z_3_) of the internal frames of the three residues defines the tilt angle (θ_t_). It essentially describes the orientation of the residue (principal) plane **(see Methods, section: Geometry of three-node packing motifs) **with respect to the triangle plane. As is well known, the angular distribution of two randomly oriented vectors should fall of as a function of sin θ' dθ'/2 (where θ' is the angle between the two vectors) [45] and the deviation of an actual observed distribution from one which is random can be estimated by means of χ^2^. Examination of χ^2 ^of θ_t _shows that for triplet cliques composed of at least one aromatic, their corresponding tilt angles (θ1_t_) exhibit significant deviation from randomness. Compositions such as Phe-Leu-Leu (χ^2 ^(θ1_t_) = 72.1), Phe-Leu-Val (60.0), Phe-Ile-Leu (48.5), Tyr-Leu-Leu (46.7), Tyr-Phe-Leu (44.6), Tyr-Ile-Leu (39.0), Phe-Ile-Val (37.7), Tyr-Leu-Val (35.5) etc (Table 4) indicates a preferred orientation of the aromatic ring plane (residue: 1 in the triplet) with respect to the global triangle plane. The actual distribution of the angles (θ_t_) shows the angular bins 60-90°, 90-120° to be preferentially populated (with respect to a random distribution) in contrast to ranges 0-30°, 150-180°, 30-60°, 120-150°, which exhibit a corresponding depletion (Table 5). Thus, both the angular distribution and visual inspection of the triplet cliques indicate that for bulky aromatics, their normals (to the residue plane) tend to be perpendicular to the global Z axis, as if the side chain tend to enclose the volume demarcated by the edges of the triangle, rather than penetrating into its perimeter (Figure 9). The other residues (Ile, Leu, Val) however did not exhibit any consistent specificity in their tilt.

**Table 4:** χ^2 ^for angular variables for triplet clique compositions exhibiting specific geometry

Composition			Frequency	**χ**^**2**^**(θ1**_**t**_**)**	**χ**^**2**^**(θ2**_**t**_**)**	**Χ**^**2**^**(θ3**_**t**_**)**	**χ**^**2**^**(φ1**_**s**_**)**	**χ**^**2**^**(φ2**_**s**_**)**	**χ**^**2**^**(φ3**_**s**_**)**
PHE	ILE	LEU	276	48.5	11.2	35.8	15.3	14.5	5.8

PHE	LEU	VAL	246	60.0	20.6	5.2	14.0	8.5	6.8

PHE	LEU	LEU	237	72.1	25.5	13.5	13.9	20.8	3.8

TYR	ILE	LEU	151	39.0	2.9	7.3	20.5	16.0	10.0

PHE	ILE	VAL	150	37.7	9.3	16.7	19.4	4.9	19.5

TYR	PHE	LEU	117	44.6	6.2	2.8	18.5	5.7	10.6

TYR	LEU	LEU	104	46.7	15.2	8.6	6.4	5.3	16.4

TYR	LEU	VAL	98	35.5	8.7	9.5	7.4	3.1	6.6

PHE	VAL	VAL	88	21.6	24.9	5.1	14.1	4.2	4.5

TYR	PHE	VAL	77	20.3	3.0	4.0	9.0	10.2	2.0

TYR	ILE	VAL	69	23.4	2.5	3.8	4.5	2.0	4.7

TRP	LEU	LEU	47	29.5*	4.0*	5.1*	3.9	17.7	2.9

TRP	LEU	VAL	41	23.9*	5.3*	3.0*	6.3	6.3	3.9

χ^2 ^of tilt angles (θ1_t_, θ2_t_, θ3_t_) and swivel angles (φ1_s_, φ2_s_, φ3_s_) of residues constituting the clique (where 1, 2, 3 corresponds to the same sequence of residues given in the table e.g., PHE → 1, ILE → 2, LEU → 3 for the first entry) for compositions showing significant deviation in θ1_t _from a random distribution. **χ**^**2**^_**0.05 **_for three-bin and six-bin models are 5.991 and 11.071, respectively. Compositions which have a predicted frequency of less than 5 for any particular angular bin, assuming a random distribution are marked with an asterisk (*). This minimal number (of data points) is 37 for a 3-bin and 74 for a 6-bin model for tilt (θ_t_) angles and 30 for a 6-bin model for swivel (φ_s_) angles.

**Table 5:** Distribution in θ1_t _for triplet clique compositions exhibiting high χ^2^

				% Occupancy in bins with θ (deg.) range					
**Composition**			**χ**^**2**^**(θ1**_**t**_**)**	**0-30**	**30-60**	**60-90**	**90-120**	**120-150**	**150-180**

**Random (6bin)**:				6.7	18.3	25.0	25.0	18.3	6.7

**Random (3bin)**:				13.4	36.6	50.0	-	-	-

PHE	ILE	LEU	48.5	4.0	26.1	69.9	-	-	-

PHE	LEU	VAL	60.0	4.5	21.1	74.4	-	-	-

PHE	LEU	LEU	72.1	2.1	21.1	76.8	-	-	-

TYR	ILE	LEU	39.0	2.0	23.8	74.2	-	-	-

PHE	ILE	VAL	37.7	4.0	21.3	74.7	-	-	-

TYR	PHE	LEU	44.6	1.7	17.9	80.4	-	-	-

TYR	LEU	LEU	46.7	0.0	17.3	82.7	-	-	-

TYR	LEU	VAL	35.5	2.0	18.3	79.7	-	-	-

PHE	VAL	VAL	21.6	0.0	28.4	71.6	-	-	-

TYR	PHE	VAL	20.3	3.9	20.8	75.3	-	-	-

TYR	ILE	VAL	23.4	1.4	20.3	78.3	-	-	-

TRP	LEU	LEU	29.5	0.0	2.1	44.7	44.7	6.4	2.1

TRP	LEU	VAL	23.9	2.4	7.4	43.9	43.9	2.4	0.0

Angular distribution of θ1_t _in different angular bins (3-bin models for Phe and Tyr and 6-bin model for Trp: 30° bins) for compositions that have shown significant deviations from a random distribution:

In order to investigate the rotation of the residue planes (XY plane of the residue-internal frames) about an axis parallel to their own internal Z, the component of the global Z axis of the triangle was projected onto the respective planes and the orientation of this vector (Zp) with respect to the internal X axis (defined as the swivel angle φ_s _ranging from 0-360°) was computed. Since the angle φ_s _is restricted to a plane, each quadrant is expected to be equally populated for a random distribution. χ^2 ^in φ_s _did not appear to show any significant preferences for any residue. Therefore, for a given tilt, the residue plane can adopt multiple orientations about an axis perpendicular to it.

In order to determine whether the formation of triplet cliques was due to either long or short range contacts, the position of residues along the polypeptide chain was examined. The sequence separation of two residues (involved in triplet clique formation) was termed local when they were separated by less than (or equal to) 10 contiguous residues and non-local when greater than 10. Overall, out of a total of 26769 clique forming contacts, 6585 were local and 20184 non-local. The same calculations repeated for polypeptide chains distributed in bins with 75-150, 151-300, 301-500 residues followed the same trends. Thus, although the majority of contacts were non-local, a non-negligible fraction (~ 25%) are between residues closely located along the polypeptide chain.

As has been mentioned previously **(section: Packing motifs)**, motifs from the f2 family were relatively favored in α|β proteins and disfavored in the all α class. The effect was accentuated for the set of all closed triplet cliques (isolated and embedded) wherein the propensities for the classes were - all α (0.53), all β (0.88), α|β (1.45) and α+β (0.87).

**Figure 12:**
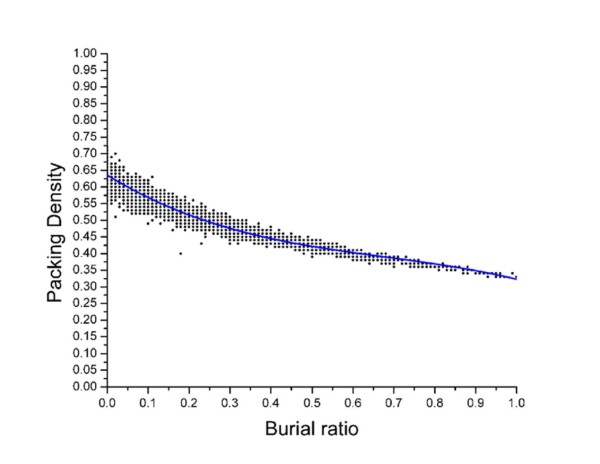
**Packing density as a function of burial ratio**. Packing density decays with increasing burial ratio (which is an index of the exposure to the solvent) following a cubic polynomial (plotted for tyrosine).

**Figure 13:**
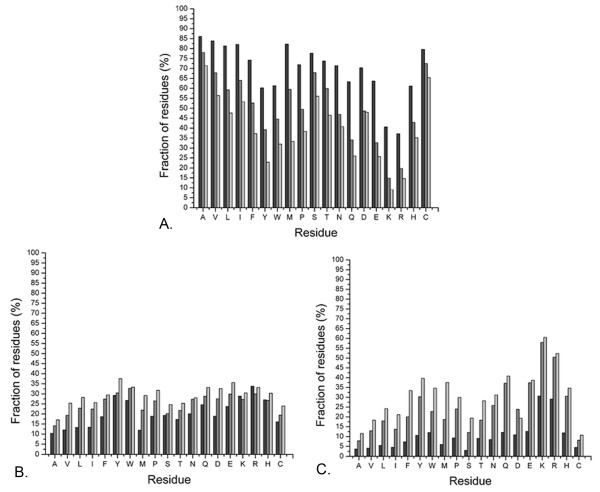
**Nodes of a clique exhibit greater propensity to get completely buried**. Percentage fraction of individual residues categorized into clique-nodes (dark gray), non-clique nodes of clique containing networks (gray) and nodes of networks devoid of cliques (light gray) sorted according to their exposure to solvent in terms of burial ratio: (**A)**. for burial ratio < = 0.05 (completely buried), **(B)**. for 0.05 < burial ratio < = 0.15 (partially buried with lower exposure to the solvent), **(C)**. for 0.15 < burial ratio < = 0.3 (partially buried with higher exposure to the solvent).

Calculations were also carried out to quantify local packing densities **(see Methods, section: Packing density) **in and around triplet cliques and also in their absence. Plots of packing density (f(x)) versus burial ratio (x) **(see Methods, section: Burial ratio) **exhibited an almost identical correlation for all the residue types (Figure 12), decaying as a cubic polynomial (*f *(*x*) = *a.x*^3 ^+ *b.x*^2 ^+ *c.x *+ *d*), demonstrating loose packing with higher exposure to the solvent. Networks were distributed into two major categories, those with triplet cliques and those devoid of them. The former were further subcategorized into the set of clique-nodes alone and that of the other non-clique members. It was evident from the results that the clique-nodes are predominantly completely buried (burial ratio < = 0.05) and thus on an average, more tightly packed than the other non-clique members whose average exposure to the solvent was consistently found to be higher (Figure 13). These regions of high local packing densities occur at or near the cliques with gradual decrease at the periphery. On the other hand, networks devoid of triplet cliques are on an average less tightly packed as a consequence of higher exposure to solvent.

### Utility of surface contact networks in fold recognition - a case study

Finally, an attempt was made to probe the efficacy of surface contact networks in correctly identifying sequences (situated amidst decoys) consistent with a given fold. Cyclophilin-like fold (pdb code: 2HAQ) was chosen as a test case and two decoy sets (a) random sequences and (b) spliced sequences from other folds, both of the same length as 2HAQ (166 residues) were generated and threaded onto the 2HAQ backbone template **(see Methods, section: Decoy sets and threading)**. Each decoy set consisted of 250 sequences. In addition to 2HAQ, two more sequences (native to the given fold, from pdb files: 3BO7_B and 3KOP_F) having sequence identities of 37% and 11% (with 2HAQ) respectively were also included, the latter to ascertain whether the method worked for sequences with low identities, yet mapping to the same fold. Surface contact networks of 2HAQ and 17 other close homologues (sequence identities upon structural alignment with 2HAQ > = 40%, rmsd < 1.5 Å) were rigorously analyzed **(see Methods, section: Topological fold detection measures) **to identify a subset of links in 2HAQ where each link was conserved in (at least 80% of) the homologues and thus could be regarded as a characteristic signature, representative of the fold. This fold specific subgraph, {Lcyp} consisting of 31 links between 36 nodes (mostly buried, hydrophobic residues) spans the three dimensional structure of the entire protein in terms of spatially connecting almost all the secondary structural elements (additional file 15: Table S7). They were also found to have appreciably high surface complementarity (average S_m_: 0.608 (0.07), min: 0.48, max: 0.71) and overlap (average Ov: 0.15 (0.03), min: 0.1, max: 0.21). In order to characterize the corresponding subgraph of {Lcyp} in a threaded structure, all nodes in {Lcyp} were mapped in that structure **(see Methods: Topological fold detection measures) **and the corresponding nodes extracted along with all their inter-connecting links. This (induced) subgraph (in the threaded structure) was then compared with {Lcyp} (serving as a template) by means of two complementary topological measures (*snet, dnet*) defined **(see Methods, section: Topological fold detection measures) **to quantify the compatibility of a sequence to a given fold. *snet *essentially evaluates the fraction of links in {Lcyp} that are conserved in the corresponding subgraph, whereas *dnet *estimates the dissimilarity in the topological pattern between {Lcyp} and the corresponding subgraph in terms of an abstract distance measure. Thus, for the corresponding subgraph extracted from the 2HAQ native structure, *snet *will attain its highest possible value of 1.00 and *dnet *will be very close to its lowest possible value of 0.00.

Statistical analysis involved computing *snet, dnet *and estimating their mean (μ) and standard deviation (σ) for the 250 decoys of each set. For the sequences 2HAQ, 3BO7_B and 3KOP_F, the same sequence was threaded 250 times (onto the backbone template of 2HAQ), each time with a randomly selected set of side chain conformers **(see Methods, section: Decoy sets and threading)**.

The statistical parameters (μ, σ) for both the decoy sets were in very good agreement. Average values of *snet *were found to be fairly low for both the decoy sets (Table 6) compared to the native sequences 2HAQ, 3BO7_B and 3KOP_F, the difference in their means being about 2 - 3 times the value of σ_decoy_. Again the highest average *snet *was obtained for 2HAQ followed by 3BO7_B and 3KOP_F. The fact that 3KOP_F was effectively discriminated from the decoys demonstrated the possible utility of the method in identifying such cases (without actually solving for the optimal side chain packing arrangement). *dnet *was also able to differentiate between decoys and sequences native to the fold even though with marginally lower significance (~ 1.5 - 2.5 times σ_decoy_). Thus, even this limited calculation appeared to indicate the ability of surface contact networks to correctly identify native sequences from decoys. All statistical parameters were found to be by and large stable over 100, 150, 200 and 250 files.

**Table 6:** Topological measures to discriminate between native and decoy sets - a case study on the cyclophilin-like fold

Category	Similarity (*snet)*			Distance *(dnet)*		
	**Mean**	**Min**	**Max**	**Mean**	**Min**	**Max**

Random Sequences	0.156 (0.093)	0.032	0.452	0.880 (0.081)	0.650	1.000

Sequences from other folds	0.143 (0.097)	0.000	0.387	0.880 (0.079)	0.676	1.000

2HAQ	0.405 (0.089)	0.129	0.677	0.688 (0.069)	0.486	0.909

3BO7_B	0.390 (0.081)	0.194	0.613	0.734 (0.060)	0.558	0.880

3KOP_F	0.307 (0.063)	0.032	0.484	0.766 (0.049)	0.639	0.969

250 files from each category, two decoy sets and three sequences native to the cyclophilin-like fold were used in the calculations. For all sequences, side chain conformers were assigned randomly. 3BO7_B and 3KOP_F has 37% and 11% sequence identity to 2HAQ. Standard deviation for each measure is given in parenthesis.

Apart from fold detection, these measures can also be used to study networks in general by actually quantifying their resemblance and/or dissimilarity. In other words, both *snet *and *dnet *should precisely reflect and sort networks according to varying degrees of likeness compared to a template. In order to benchmark these functions, {Lcyp} was compared with corresponding subgraphs from seven other homologous structures from the cyclophilin-like fold (independent of the 17 which were use to construct {Lcyp}), with gradually decreasing sequence identities ranging from 39% to 10% upon structural alignment with 2HAQ. Prior to computing the topological measures, each structure was aligned to 2HAQ to generate a one-to-one mapping of their residues. These maps were utilized to extract the corresponding subgraphs of {Lcyp} from the homologous structures. In case of insertions-deletions or non-alignment, the rows and columns corresponding to the missing node(s) (in the related adjacency matrix of the homologue) were zero-padded. Both the measures *snet *and *dnet *exhibited excellent correlations with respect to sequence identity and root mean square deviation (between CA atoms) of the homologues upon structural alignment to 2HAQ (Table 7). These results appeared to validate the ability of these topological measures to correctly assess the resemblance of fold specific subgraphs.

**Table 7:** Validation of the topological measures (*snet, dnet*) used to compare fold specific subgraphs

PDB ID	Sequence identity (%)	Number of residues aligned	Rmsd (Å)	Similarity *(snet)*	Distance *(dnet)*
2POE_A	39	152	1.2	0.806	0.390

3BO7_B	37	152	1.5	0.742	0.489

2NUL_A	37	147	1.8	0.774	0.385

2OSE_A	30	153	2.1	0.548	0.614

1ZX8_A	15	108	2.5	0.516	0.679

3KOP_F	11	126	2.8	0.484	0.712

2P0O_A	10	96	2.9	0.452	0.725

7 crystal structures (from the cyclophilin-like fold) with varying sequence identities (less than 40% w.r.t. 2HAQ) were superposed onto 2HAQ and the corresponding subgraphs of {Lcyp} constructed, prior to the estimation of *snet, dnet*. Both the topological measures show very high correlations with sequence identities as well as root mean square deviation upon structural alignment with 2HAQ.

## Conclusions

The work presented here is based on the confluence of two related though distinct ideas, (1) some network topologies are preferred within protein interiors, leading to the concept of packing motifs and (2) the 'jigsaw puzzle' model can be successfully extended into the domain of protein contact networks

The implementation of both these ideas depends partly on representing the internal architecture of proteins in terms of surfaces rather than point atoms. It has been noted previously that use of surfaces improved the performance of a side chain (torsion) prediction test [46,47] and provided simple well defined criteria to identify those contacts which definitely constrain inter-residue geometry of the associating amino acid side chains [26]. Presumably these set of interactions could be playing a more critical role in sustaining the native fold. Networks based on surface contacts (with appropriate cut offs on S_m _and Ov) is in effect a straightforward extension of the jigsaw puzzle model. In the search for compositional or geometrical bias, surface contact networks appear to be indispensable. In particular, triplet cliques composed exclusively of hydrophobic residues had a frequency 3 fold higher in ASCN than APCN starting from a comparable (total) number of triplet cliques. Further more, compositional preferences along with strong geometrical constraints were far better explored by surfaces than point atoms (additional file 16: Table S8).

One feature which appears to be more or less conserved in surface contact networks (irrespective of the cutoff criteria in surface complementarity and overlap) is the almost ubiquitous presence of smaller networks (3-10 nodes) in all proteins which probably coalesce to produce larger networks specific to the particular fold. Thus, the distribution in network sizes and topologies appear to favor a nucleation-condensation phenomenon [48,49] in protein packing wherein open linear chains, closed triplet cliques and other closed ring topologies could serve as basic packing units which could either get linked or recruit neighboring residues to grow into networks of larger size. This notion of packing units led to the definition of 'packing motifs', which could serve as a 'basis set' in the assembly of extended graphs.

Since graphs ranging from 3 to more than 200 nodes have been detected in proteins, the concept of a 'basis set of motifs' should represent sets of similar topologies (along with their variations in terms of linear branching) rather than a rigid set of isolated unique graphs. This was the rationale behind the organization of motifs into families (or set of similar graphs with gradual addition of nodes following a path such that the core topology remains unaltered) and it soon became clear that some families were overwhelmingly preferred in protein topological space. These families emanated from the 'minimally connected' open linear chains and three residue cliques (regarded as clustering units) and cast their dominant influence in frequency distribution of motifs. Other families occurred with such abysmally low frequencies that they could be considered oddities rather than the rule. Thus, in accord with the inductive approach of the current work, it was felt that larger graphs (n > 10) would either fall into pre-existing families or could be assembled by known motifs or their variants. This possibility was explored for networks of 15 nodes and the observations tended to support the hypothesis.

One major drawback of the present study is the lack of a computational method to analyze larger graphs. Although, starting from the novel numerical scheme (Figure 3) a suitable algorithm was able to identify all possible induced subgraphs of a given larger graph, yet manual intervention was indispensable to resolve the larger graph into an optimal set of constituent motifs. Despite its rigor the tedious visual method restricted the analysis to a small fraction of the network space. However, even with this handicap, the general trend appears to be unmistakable. It thus appears that the topological space available to protein contact networks is severely constrained with clearly defined preferences. A unique constraint is the definite upper bound found in the size of the maximal clique (n_c _< = 4) - a property rarely observed in real world networks [50]. Obviously, this is due to the atomic environment in protein interiors that restricts the permissible number of edges a node can have. Thus, although only a limited portion of the topological space has been actively explored, the conclusions are expected to be significantly in the right direction.

The next step was to enquire whether packing motifs exhibited any preferences in terms of their constituent residues and geometry. For this, triplet cliques were selected due to their ubiquitous presence primarily as induced subgraphs embedded in larger graphs. It soon became evident that in the sub-space of hydrophobic residues, regular trends of propensities favoring specific residues or their combination do indeed exist and certain geometrical features exhibit very strong constraints (especially the approximately equilateral triangle connecting the three residue-origins and the tilt angles of aromatic residues). What is perhaps notable is that these compositional and geometric preferences stand a possibility of detection only when statistical analysis is performed subsequent to the precise classification of motifs and the appropriate partitioning of the topological space. In other words, looking for preferences in case of a pooled set of three residue graphs or subgraphs without adequate classification/characterization is most likely to end in failure.

The most direct application of this study should be in the area of protein fold recognition which is to select a polypeptide chain belonging to a particular fold [21,51-53] from a set of decoys. The most challenging aspect of this problem is to identify those chains consistent with a fold (represented by a set of main chain coordinates), even though identity upon alignment with the sequence (native to the three dimensional structure) is significantly low (< 20%). Preliminary calculations show that topological measures (*snet *and *dnet*) defined on surface contact networks are indeed able to discriminate sequences native to a particular fold (cyclophilin-like) from decoy sets. Although the threading procedure was fairly straightforward and the decoy sets rather limited, yet all indications appeared to suggest the robustness of the fold prediction method. Most probably the scores could be improved by the adoption of more sophisticated threading procedures which actually solves for the most optimal side chain packing arrangement, rather than averaging over a large number of random side chain conformers. However, the interesting fact is that the native sequences specific to a particular fold could be distinguished from the decoy sets, based on the statistics of the topological measures alone, despite having randomized side chain conformers. The utility of these functions to sort networks based on topological resemblance (with respect to a template) is also notable. Large scale improvement and application of these methods are currently being investigated.

Another fruitful area of research could be to explore the possibility of introducing triplet cliques into designed proteins to stabilize their packing analogous to the engineering of disulfide bridges, in order to improve thermal stability. A library of triplets exhaustively documenting their conformational, geometrical and topological features might be useful in this regard.

Thus in conclusion, it appears that out of innumerable topological possibilities, only a finite number are actually realized in protein contact networks which either are themselves or could be assembled from a limited set of preferred motifs. One such recurrent motif, the triplet clique, exhibits clear preferences in its constituent residues and very strong constraints with regard to certain geometrical features.

## Methods

### The Database

Initially, 918 unique protein crystal structures were selected from the protein data bank (RCSB-PDB) [54] with a maximum R factor of 20% and a resolution cutoff of 2.0 Å. Upon sequence alignment of any two proteins from the database, in no case was their sequence identity greater than 30%. For oligomeric proteins the largest polypeptide chain was retained and for atoms with multiple occupancies, those with the highest occupancy were used in the calculations. In case of equal occupancy the first conformer was selected. Proteins with incomplete side chain atoms and those with missing stretches of amino acid residues were individually surveyed in RasMol [55]. If the missing stretch(s) or residue(s) involving incomplete side chain atoms was found to be either in the extremities (N/C terminal) of the chain or on completely exposed loop regions with no participation in interior packing, the protein was included in the database, otherwise rejected. The length of the chains ranged from 75 to 500 amino acid residues. The final database consisted of 719 polypeptide chains of which 18.5% was all α, 19.9% - all β, 32.5% - α|β and 29.1% - α+β (additional File 17: Table S9). The protein class for each chain was decided by visual examination in Rasmol and a search in the SCOP database [56]. 53 multidomain proteins were appropriately truncated and their domains allotted to the relevant class. The program Reduce [57,58] was used to geometrically fix hydrogen atoms on the proteins prior to the calculations.

### Burial ratio

The exposure of residues to solvent (probe radius 1.4 Å) was estimated by the ratio (burial) of solvent accessible areas (SAA) of the amino acid, X in the polypeptide chain to that of an identical residue located in a Gly-X-Gly peptide fragment with a fully extended conformation. Residues that were completely (0.00 < = burial ratio < = 0.05) or partially buried (0.05 < burial ratio < = 0.3)) were only considered in the analysis.

### Algorithm to construct networks

As is well known every network can be represented as a graph, *G *= (*V, E*) which formally consists of a set of vertices (or nodes) *V *and a set of edges (or links) *E *between them. Trivially a graph can contain one or more standalone nodes (a node which is not connected to any other node in the graph) and a subgraph is called a component [41] of the graph provided each node is connected at least to one other node of the graph. In protein contact networks to be defined, no standalone node was considered. Thus, in this context, 'graph' and 'component' were treated synonymously. In the present study, a nodal point stands for the side chain of a particular residue, and two types of networks were defined based on surfaces and point atoms. For the case of point atoms, if any two atoms located on two different side chains were within 3.8 Å of each other, the two representative nodes were connected by a link. The number of atomic contacts between two side chains was considered to be the weight of the connecting edge. The network spanning the entire protein was constructed by exhaustively searching for contacts in the neighborhood of buried residues until no more nodes could be included in the network. Thus a protein could have more than one contact network embedded within it with no common nodes between them. The smallest networks considered had three nodes. With the exception of glycine any residue could be represented by a node.

### Van der Waals surface generation

The van der Waals surfaces for the proteins (including all hydrogen atoms) were sampled at 10 dots/Å^2^, the atomic radii being assigned from the all atom molecular mechanics force field [59]. The details of the surface generation have been discussed elsewhere [26]. In case of disulphide bridges care was taken to remove the extra points due to the interpenetration of the van der Waals spheres of the covalently linked sulphur atoms. Thus, the entire surface of the polypeptide chain was sampled as an array of discrete area elements defined by their location (x,y,z) and the direction cosines (dl,dm,dn) of their normals.

### Surface Complementarity

Based on the van der Waals surface, surface complementarity (Sm) [60] and overlap (Ov) were defined as in a previous report [26]. Briefly, for a surface point (a) located on a buried side chain (referred to as a target), its nearest neighbor (b) was identified from the surface points of its surrounding residues, within a distance of 3.5 Å. Then the following expression was computed:

where **n**_***a ***_and **n**_***b ***_are two unit normal vectors corresponding to dot surface points a and b respectively, with *d*_*ab *_the distance between them and *w *a scaling factor, set to 0.5. Thus for a target, a distribution of *S *values was obtained for all its side chain dot surface points. The surface complementarity (S_m_) for a particular target was defined as the median of this distribution {*S(a,b)*}. The entire side chain surface of a target can be partitioned into patches based on the neighboring residues whose surface point(s) were identified as its nearest neighbors. For a specific target (A) and neighbor (B) the overlap (Ov^A→B^) between them was defined as

where N_AB _is the number of points on the target (A) that have their nearest neighboring points on B and N_A _is the total number of surface points for A. The surface complementarity for this patch involving A, B will henceforth be referred to as S_m_^A→B^. Contact between any two residues (target and neighbor) can now be defined in terms of surfaces (based on S_m _and Ov). Any two residues (target: A, neighbor: B) are said to 'interact' with each other when S_m_^A→B^, Ov^A→B ^are greater than equal to 0.4 and 0.08 respectively. It will be noted that the measures of S_m _and Ov are non-commutative, that is S_m_^A→B ^, Ov^A→B ^are not necessarily equal to S_m_^B→A ^and Ov^B→A^. We formally define inter-residue surface 'contact' when their 'interactions' are mutually reciprocal, that is both S_m_^A→B^, S_m_^B→A ^and Ov^A→B^, Ov^B→A ^simultaneously satisfy the interaction criteria. For any contact, S_m _and Ov were taken to be the mean of (S_m_^A→B ^, S_m_^B→A^) and (Ov^A→B^, Ov^B→A^) respectively. Similar to point atom contact networks a node in this case is also representative of the residue side chain (surface). Two nodes are connected by an edge when their corresponding residue surfaces are in 'contact'. Weight of such an edge was defined as , analogous to calculating the magnitude of two mutually orthogonal vector components. Based on the definitions given above such networks, henceforth referred to as 'surface contact networks', will also be undirected.

Thus, two distinct types of networks have been defined and used in this study (1) All Residue Surface Contact Network (ASCN) and (2) All Residue Point Atom Contact Network (APCN). All contact networks were represented computationally in terms of one-zero adjacency matrices, (N×N, for a network of N nodes) where the matrix element *a*_*ij *_= 1 denotes node *i *to be connected to node j and 0 otherwise. Since both types of networks were undirected, these adjacency matrices were essentially symmetric. Based on the adjacency matrices the following network parameters were estimated:

**Degree**: defined as the number of edges emanating from a node.

**Strength of a node**: defined as the sum of the weights of all edges of a node, *i *given by:

where *w*_*ij *_is the weight of the edge linking the *i*^*th *^and the *j*^*th *^node and the summation is over all nodes (*N*) of the network [40].

#### Unweighted and weighted clustering coefficients

Expressions for these coefficients are defined as follows:

#### Unweighted

where *k*_*i *_is the degree of the *i*^*th *^node and *|{e*_*jh*_*}| *is the total number of actually existing connections among the set of nodes (taken pairwise, {j,h}) from the direct neighborhood of node *i *and ^*ki*^*C*_*2 *_is the number of maximum possible connections within the same set [39].

#### Weighted

where the symbols have the same significance as given above and under identical conditions [40].

#### Cliquishness

Clique is an induced subgraph where every node is connected to every other node. In case of an undirected graph containing a clique of n nodes, the embedded clique should contain ^n^C_2 _edges. On the other hand, a complete graph will have any two nodes connected to each other. In this analysis the term 'isolated clique' refers to such complete graphs. Order (or size i.e., the number of constituent nodes, n_c_) of the maximal clique was searched progressively in all networks starting from triplets. Initially, a systematic search for all possible combinations of 3 nodes (from a network) was performed to identify the closed triplet cliques and on occurrence, n_c _was set to 3. Then from the immediate neighborhood of a 3-clique, each node was sampled to test for adjacencies with all three nodes of the clique. A new node, on satisfaction of this criterion, was added to the previous clique and n_c _was increased by one. The search was continued till convergence.

### Deviation from random topology

To estimate deviation from a random topology, unweighted and weighted clustering coefficients were individually averaged over all nodes in a network and were compared with the same measure obtained for random graphs of identical size. Following standard methods, first, the link density (***L***_***d***_) of a graph was estimated, defined as the ratio of the total number of actually existing edges in the graph and the number of maximum possible edges if it were a complete graph. Random graphs of identical size were generated by systematically calling each pair of nodes along with a random number seed and the pair was assigned a weighted connection if the random number was found to be lesser than the corresponding ***L***_***d ***_value obtained from the original graph. Weight of an edge was also assigned randomly, scaled appropriately to the values obtained from the observed contact networks.

### Geometry of three-node packing motifs

The methodology of Singh and Thornton [45] was adopted to identify preferred modes of packing in terms of the specific geometry of interacting amino acid side chains. An internal right handed frame of reference was defined for all the hydrophobic residues based on their side chain atoms. Conventionally, the Z axis was taken to be normal to the principal plane defined by either the ring atoms (phenyl for Phe, Tyr and indole for Trp) for aromatic residues or a defined set of three side chain atoms (forming the fork) for branched chain amino acids (Val, Leu, Ile) (Figure 10).

To characterize the geometry of graphs or subgraphs consisting of three nodes, a plane, P_triangle _was defined passing through the origins of the three internal frames of reference (Figure 11). The resulting triangle defined by connecting the three origins was characterized by three internal angles Ω_1_, Ω_2 _and Ω_3 _and the lengths of the three sides of the triangle r_12_, r_13_, and r_23. _A preferred right handed frame was placed at the centroid of this triangle such that the X axis (X_tr_) points towards the origin of a preferred residue chosen according to the composition of the triplet, **(see Results, section: Triplet Clique)**, the Z axis (Z_tr_) taken normal to P_triangle _and Y_tr _= Z_tr _× X_tr _. Three inter-planar tilt angles namely **θ1**_**t**_, **θ2**_**t **_and **θ3**_**t **_were then defined as angles subtended between Z_tr _and the Z axes of the three residue-internal frames. Three additional swivel angles **φ1**_**s**_, **φ2**_**s**_, **φ3**_**s **_were further defined as those subtended by Z_p _(the component of Z_tr_, projected on residue XY planes) and the X axes of the three residue-internal frames. The distributions of these angles in appropriate bins were analyzed for their deviation from a random distribution by means of χ^2^. The distribution in the angle subtended by two randomly oriented vectors has probability density given by sin θ' dθ'/2, where θ' is the angle between the vectors [45] whereas for two coplanar random vectors each bin should be equally populated. Thus, for a random distribution, the probability of θ1_t_, θ2_t_, θ3_t _falls as a function of sin θ' dθ'/2 (three-bin models for Phe and Tyr and six-bin models for Val, Leu, Ile, Trp: 30° bins) and each bin should be equally populated for φ1_s_, φ2_s _and φ3_s _(six-bin models for Phe, Tyr, Trp, Val, Leu, Ile: 60° bins).

### Packing density

Packing density is conventionally defined as the ratio of the volume enclosed by the van der Waals (VDW) envelope for an atom, atomic group or molecule to that of the actual volume occupied by it in space, conventionally taken to be its Voronoi volume [2,61] (which is the volume of a polyhedron, systematically extended around the atomic group until it comes into contact with similar polyhedra in its neighborhood). The program Voronoia.exe [62,63] was used to compute local packing densities around residues within a polypeptide chain. In this software, instead of voronoi volume, solvent excluded (SE) volume [64] of the atomic group (defined as the space which is not accessible to any center of solvent spheres, calculated by rolling a solvent sphere of 1.4 Å probe radius over the protein surface) is calculated. Then packing density is given by the following ratio:

The method is considered an improvement over previous algorithms due to the fact that cavities are critically distinguished and eliminated from the actual spaces between two molecular entities and also the neighboring surfaces are cut about non planar boundaries.

#### Propensity

Propensity (*P(f/C)*) for a family of motifs (*f*) to belong to a particular class (*C*) was calculated by the equation:

where *N*_*fC *_is the number of motifs '*f*' found in chains belonging to class *C, N*_*f *_is the number of motifs '*f*' found in all classes, *N*_*C *_is the number of chains belonging to class *C *in the database and *N *is the total number of chains in the database. *C *stands for one of the classes (all α, all β, α|β, α+β).

Similarly, propensity (*P(S/C)*) for a network of a given size (*S*) to belong to a particular class (*C*) was calculated by the equation:

where *N*_*SC *_is the number of networks of size *S *found in chains belonging to class *C, N*_*S *_is the number of networks of size *S *found in all classes and *N*_*C*_, *N, C *have same significance as above.

### Decoy sets and threading

To study the application of surface contact networks in fold recognition, cyclophilin-like fold (pdb code: 2HAQ) was selected as the test case and two decoy sets were assembled, of 250 sequences each. The first set consisted of random sequences of the same length (166 residues) as 2HAQ and the second was composed of naturally occurring 166 residue stretches truncated from the N terminal end, from other folds. In general, the sequence identity of these decoys w.r.t. 2HAQ was less than 10% and no two sequences in each decoy set had identities greater than 15% between them. To determine whether the fold recognition method could identify sequences compatible with the same fold (cyclophilin-like) even though exhibiting low sequence identity (less than 20%) with 2HAQ, the method was tested on the sequence extracted from 3KOP_F (11%). In addition, another chain, 3BO7_B (37%) was also included. To simplify matters, 3KOP_F and 3BO7_B were purposely chosen as their native chain lengths were identical to that of 2HAQ.

To start with, the actual three dimensional coordinates of all the residue conformers as listed in Dunbrack's rotamer library [65] were generated. The main chain coordinates were extracted from 2HAQ and was considered to be representative of the cyclophilin-like fold. For threading any sequence onto this template, the main chain N, CA, C coordinates of the appropriate residue (to be threaded) were selected from the library and superposed onto the corresponding native coordinates. For every threaded residue, the rotamer was selected randomly from the possibilities present in the library. Since CA is a tetrahedral center, this procedure automatically superposes the CB atom as well. The root mean square deviations of N, CA, C, CB atoms of the threaded structures (w.r.t. 2HAQ native coordinates) were found to be less than 0.1 Å which vouched for the correctness of the method. For each residue position, the superposed side chain coordinates of the rotamer were then appended to the original main chain coordinates of the template. Subsequent to threading, each structure was energy-minimized by 500 steps of Steepest Descents (SD) followed by 20000 steps of Adopted Basis Newton-Raphson (ABNR) method with a gradient tolerance (tolgrd) of 0.001 and a distance dependent dielectric multiplied by 4.0 using the CHARMM-22 force-field [66,67]. The constant harmonic force parameter was set to 250.0 for N, CA, C and O atoms and 10.0 for CB to conserve the main chain three dimensional representation of the fold. Every structure was checked to have reasonably acceptable geometry using PROCHECK [68]. For 3KOP_F, 3BO7_B and the native sequence 2HAQ, the threading procedure was performed 250 times, each time with a different set of randomized rotamer combination. For each threaded structure, surface contact networks were generated as described previously **(see Methods, section: Surface complementarity)**.

### Topological fold detection measures

17 additional structures belonging to the cyclophilin-like fold were chosen from the protein data bank [54] which had greater than 40% sequence identity upon structural alignment with 2HAQ (PDB ID_Chain (rmsd (Å), sequence identity (%)): 1XO7_A (0.5, 74), 3ICH_A (0.8, 65), 2PLU_A (1.3, 63), 2X25_B (1.2, 61), 2CFE_A (1.2, 60), 1QOI_A (0.8, 57), 1A58_A (1.2, 57), 1IHG_A (1.2, 57), 2R99_A (1.3, 57), 1DYW_A (1.4, 57), 2HQJ_A (1.4, 57), 2CMT_A (1.2, 56), 3K2C_B (1.4, 54), 2GW2_A (0.8, 53), 2HE9_A (0.8, 53), 2FU0_A (1.3, 47), 1ZKC_A (1.2, 42)). Surface contact networks (at S_m _> = 0.4, Ov > = 0.08) were generated for all the 17 native structures along with 2HAQ. Unlike networks defined while describing packing motifs, these networks could contain unconnected components and even isolated binary links. Here the primary emphasis was to represent a fold as a unique subset of relevant links, highly conserved amongst members of that fold. Pairwise structural alignment (using Dali Server [69]) with 2HAQ (considered to be the template) provided the mapping between the nodes of 2HAQ and each of the 17 homologous proteins. In case of insertions-deletions or non-alignment, the node was considered to be absent in the related protein. Every link in the contact network of 2HAQ was searched systematically in the 17 homologues and counted for the number of times the corresponding (mapped) nodes were found to be present and connected. Only those links from 2HAQ were retained which were present in at least 80% of the other (17) homologues. This subset of links was considered to be representative of the cyclophilin-like fold and designated as {Lcyp}.

To test for fold compatibility of any sequence threaded onto 2HAQ, two complementary topological measures (*snet, dnet*) were defined based on {Lcyp} and the corresponding subgraph in the threaded structure. It should be noted that for the threaded structure, the specification of a node was identical to that of 2HAQ depending on its residue number.

#### Similarity

The fraction of links in {Lcyp} that are conserved in the threaded structure.

where N_t _is the number of equivalent links from {Lcyp} found in the threaded structure and N_s _is the total number of links in {Lcyp}.

#### Distance

The subgraph {Lcyp} can be represented as an adjacency matrix, *A *which is considered to be an element in a vector space. The rows and columns of the matrix are the sequentially ordered array of residues which appear as nodes in {Lcyp}. For every threaded structure, an adjacency matrix, *A'*, similar to the one defined on {Lcyp} can be characterized with the same order of the residue positions (along the polypeptide chain of 2HAQ) maintained in the rows and columns. Thus, *A(i,j) *and *A'(i,j) *represents the adjacency between the same *i*^*th *^and *j*^*th *^node in graphs *A *and *A' *since their residue positions are identical in both. Distance between two such (undirected) graphs of identical size could be determined by counting the number of links that are present in one and absent in the other and then normalizing by the number of links present in either of the two graphs.

where *A(i,j) *and *A'(i,j) *are the matrix elements of adjacency matrices *A *and *A' *based on 2HAQ and the threaded structure respectively and *nL *is the number of elements in the set *E *∪ *E*' where *E *and *E' *are the sets of links corresponding to graphs *A *and *A'*. It can be shown that *dnet (A, A') *is formally a metric in a vector space (proof not given).

### Languages/Softwares used

Codes for network generation and calculation of network parameters were developed in PERL (v.5.8). Surface generation and surface complementarity/overlap calculations were performed on a DEC-Alpha server with programs written in Fortran 90. Matlab (v.7.5) was used to analyze geometry. Networks were visually analyzed using Cytoscape [43] (v.2.6.2) and related crystal structures were surveyed in RasMol [55] (v.2.4.7.2) and PyMol [44] (v.1.3). The threading program was written in Fortran 90 and energy-minimization was carried out using CHARMM [66]. Structural alignments were performed using DALI server [69].

## List of Abbreviations used

**ASCN: A**ll Residue **S**urface **C**ontact **N**etwork; **APCN: A**ll Residue **P**oint Atom **C**ontact **N**etwork.

## Authors' contributions

SB carried out the entire work and also wrote most of the computer programs necessary for the calculations. RB conceived the problem, designed the calculations and prepared the manuscript along with SB. DB provided technical guidance on the energy-minimization protocol. All authors read and approved the final manuscript.

## Supplementary Material

Additional file 1 **Figure S1. Distribution of point atom contact networks according to size**. Frequency distribution of networks of different sizes (n) for APCN follows a power law decay (Corresponding histogram is displayed in the inset, the X axis being truncated to n = 50). Click here for file

Additional file 2 **Table S1. List of proteins with very large networks**. Accession number (PDB ID), protein-class, polypeptide chain length, network size, fold, overall description of the protein and the source organism have been tabulated for each protein. Click here for file

Additional file 3 **Table S2. Distribution of networks by size amongst protein classes**. The top row contains the fraction of the polypeptide chains in each class (bold in parenthesis). Along with frequency, the propensity **(see Methods, section: propensity) **of the network of a given size to be found in a particular class (enclosed in parenthesis) is also given. Click here for file

Additional file 4 **Table S3. Correlation between number of (unique) motifs: observed in the database versus simulated from random graphs**. For a given network size (n), the number of unique motifs observed in the database is tabulated along with the corresponding number generated from simulated random graphs without and with cutoffs on the highest attainable degree of a node. Click here for file

Additional file 5 **Figure S2. Motifs belonging to families f3a and f3b**. Network diagrams of motifs up to size 7 (nodes) belonging to family f3a (left panel) and f3b (right). Motif identifier for each motif is displayed below the motif with the number of members for ASCN and APCN respectively in parentheses separated by a front slash. Click here for file

Additional file 6 **Figure S3. Motifs belonging to families f4a, f4b and f4c**. Network diagrams of motifs up to size 7 (nodes) belonging to family f4a (left panel), f4b (middle) and f4c (right). Motif identifier for each motif is displayed below the motif with the number of members for ASCN and APCN respectively in parentheses separated by a front slash. Click here for file

Additional file 7 **Figure S4. Motifs belonging to family f5**. Network diagrams of motifs up to size 7 (nodes) belonging to family f5. Motif identifier for each motif is displayed below the motif with the number of members for ASCN and APCN respectively in parentheses separated by a front slash. Click here for file

Additional file 8 **Figure S5. Motifs belonging to families f6a, f6b and f7**. Network diagrams of motifs up to size 7 (nodes) belonging to family f6a (left panel), f6b (middle) and f7 (right). Motif identifier for each motif is displayed below the motif with the number of members for ASCN and APCN respectively in parentheses separated by a front slash. Click here for file

Additional file 9 **Figure S6. Motifs belonging to families f8a, f8b and f8c**. Network diagrams of motifs up to size 7 (nodes) belonging to family f8a (left panel), f8b (middle) and f8c (right). Motif identifier for each motif is displayed below the motif with the number of members for ASCN and APCN respectively in parentheses separated by a front slash. Click here for file

Additional file 10 **Table S4. Distribution of motifs and families in each protein-class**. Motifs (obtained from ASCN, S_m _> = 0.4, Ov > = 0.08) are sorted according to size (up to 7 nodes) and grouped under their respective families. The frequency of their occurrence is given along with the propensity **(see Methods, section: propensity) **of a given class to contain a family of motifs. Other than f1 and f2, rest of the families do not have sufficient members for robust statistics. Click here for file

Additional file 11 **Table S5. Distribution of motifs and families obtained at different contact cutoffs**. Motifs are sorted according to size (up to 7 nodes) and grouped under their respective families. Cutoffs on S_m _and Ov are mentioned in parenthesis. Results for the chosen set of cutoff values, used in the analysis (0.4, 0.08) are highlighted in bold. Click here for file

Additional file 12 **Dataset S1. Surface contact networks constituted of 15 nodes resolved into optimal set of motifs**. Network diagrams of 38 contact networks of size 15 (ASCN) resolved into optimum sets of motifs (or variants) which are either components (separated by boxes) or induced subgraphs (highlighted with different colors). Families of these motifs are also mentioned. Source PDB IDs are displayed at the (right) bottom of each graph. Click here for file

Additional file 13 **Table S6. Triplet cliques constituted of hydrophobic residues exhibit preferences in their amino acid composition**. Frequency distributions of triplet clique compositions in categories (a) C1 (all three residues different), (b) C2 (two residues identical) and (c) C3 (all three identical) are tabulated respectively. Click here for file

Additional file 14 **Figure S7. Optimally superposed triangles**. Triangles (formed by joining the origins of the internal frames based on the three residues in a triplet clique) sampled from compositions belonging to categories **C1 **(all three residues different: top left), **C2 **(two residues identical: bottom left) and **C3 **(all three residues identical: right) superposed onto each other. Click here for file

Additional file 15 **Table S7: Links constituting the subset {Lcyp}**: {Lcyp} represents the subset of links in the surface contact network of 2HAQ which is highly conserved among its close homologues defining the (cyclophilin-like) fold specific subgraph. Sec str represents the location of these links with respect to secondary structural elements and lp stands for the fraction of times the link was found in the corresponding subgraph of the 17 other homologues, used to define {Lcyp}. Two nodes in the same row are connected by a link whose surface complementarity (S_m_) and overlap (Ov) are tabulated. In column 2 and 5, Sx, Hy, Lxy and Txy represents Strand x, Helix y, Loop and Turn connecting secondary structural element x and y respectively. Click here for file

Additional file 16 **Table S8. Specific geometry more clearly manifested by surfaces than point atoms**. χ^2 ^of tilt angles (θ1_t_, θ2_t_, θ3_t_) and swivel angles (φ1_s_, φ2_s_, φ3_s_) for triplet compositions for **(a) ASCN (b) APCN**. 1, 2, 3 corresponds to the same sequence of residues given in the table e.g., ILE → 1, LEU → 2, VAL → 3 for the first entry of **(a)**. **χ**^**2**^_**0.05 **_for three-bin and six-bin models are 5.991 and 11.071, respectively. Compositions which have a predicted frequency of less than 5 for any particular angular bin assuming a random distribution are marked with an asterisk (*). This minimal number (of data points) is 37 for a three-bin and 74 for a six-bin model for tilt (θ_t_) angles and 30 for a six-bin model for swivel (φ_s_) angles. Only those compositions have been given whose frequencies are greater than equal to 25. Click here for file

Additional file 17 **Table S9. The Database**. Polypeptide chains used in the analysis sorted according to class. The accession numbers (PDB ID), chain identifiers along with resolution in Angstroms and the first and last residue numbers (in case of multidomain proteins) are given in parenthesis. Click here for file
