# Supplementary figures and images for: Mapping the distribution of packing topologies within protein interiors shows predominant preference for specific packing motifs
Source: BMC Bioinformatics. 2011 May 24;12:195. doi: 10.1186/1471-2105-12-195 (PMC3123238; doi:10.1186/1471-2105-12-195)

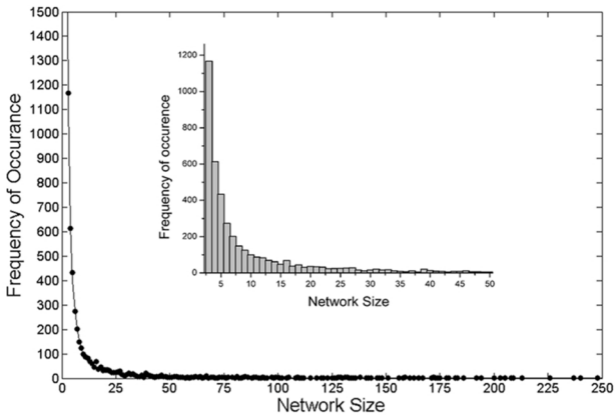

Supplement: Additional file 1 — Figure S1. Distribution of point atom contact networks according to size. Frequency distribution of networks of different sizes (n) for APCN follows a power law decay (Corresponding histogram is displayed in the inset, the X axis being truncated to n = 50). [file 1471-2105-12-195-S1.PDF]

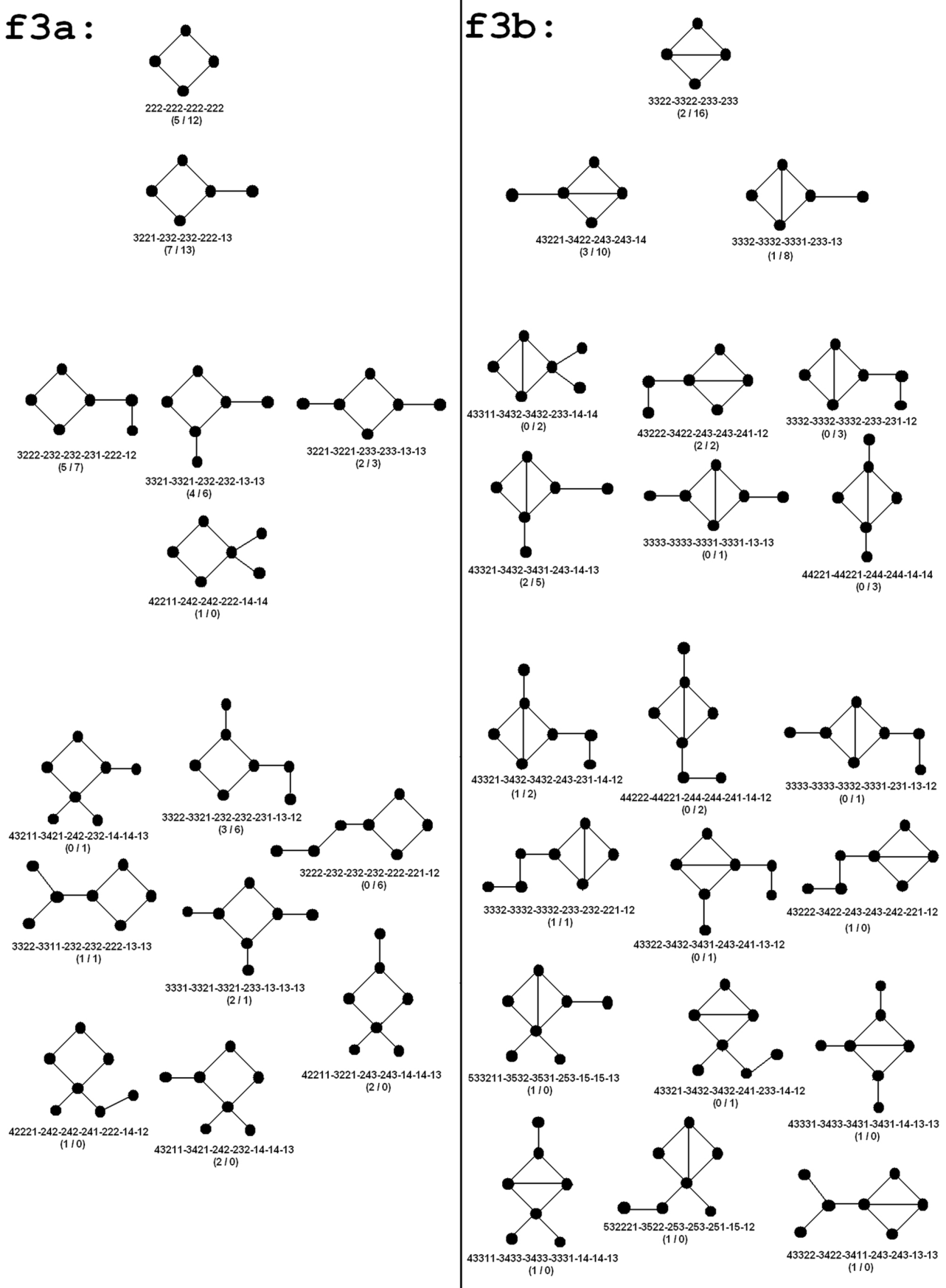

Supplement: Additional file 5 — Figure S2. Motifs belonging to families f3a and f3b. Network diagrams of motifs up to size 7 (nodes) belonging to family f3a (left panel) and f3b (right). Motif identifier for each motif is displayed below the motif with the number of members for ASCN and APCN respectively in parentheses separated by a front slash. [file 1471-2105-12-195-S5.PDF]

f4a:

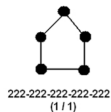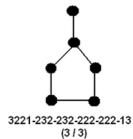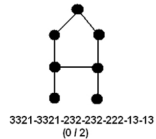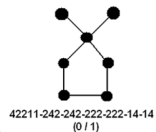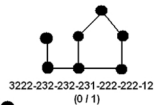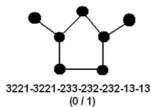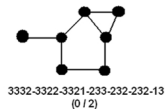

f4b:

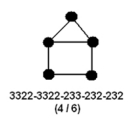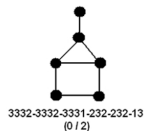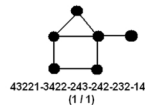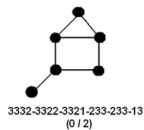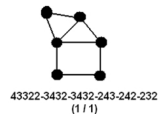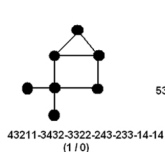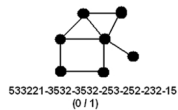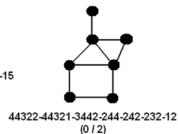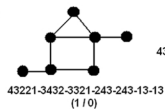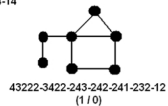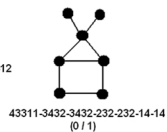

f4c:

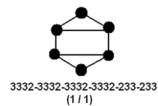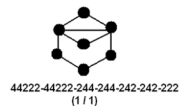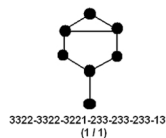

Supplement: Additional file 6 — Figure S3. Motifs belonging to families f4a, f4b and f4c. Network diagrams of motifs up to size 7 (nodes) belonging to family f4a (left panel), f4b (middle) and f4c (right). Motif identifier for each motif is displayed below the motif with the number of members for ASCN and APCN respectively in parentheses separated by a front slash. [file 1471-2105-12-195-S6.PDF]

f5:

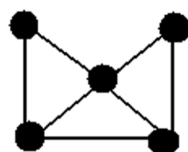

43322-3432-3432-243-243  
(0 / 2)

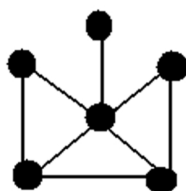

533221-3532-3532-253-253-15  
(0 / 1)

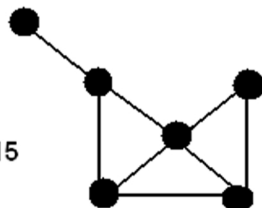

43332-3433-3432-3431-243-13  
(0 / 1)

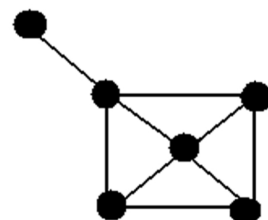

44333-44331-3443-3443-3433-14  
(0 / 1)

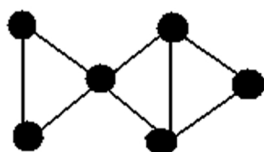

43322-3432-3432-242-242-233  
(1 / 0)

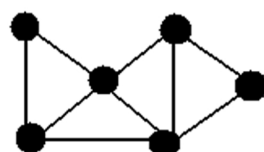

44332-44332-3442-3442-243-243  
(0 / 1)

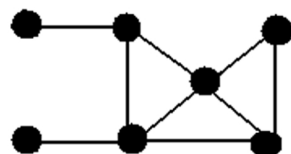

44332-44331-3442-3441-243-14-13  
(1 / 0)

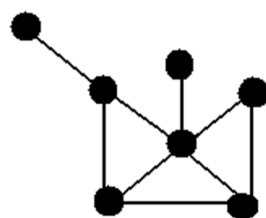

533321-3533-3532-3531-253-15-13  
(0 / 1)

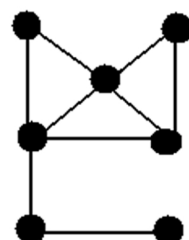

44322-44322-3442-244-243-241-12  
(0 / 1)

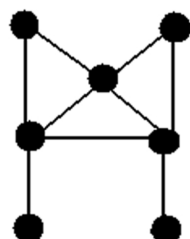

44422-44421-44421-244-244-14-14  
(1 / 1)

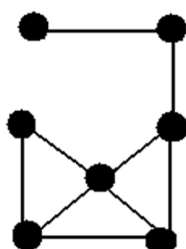

43332-3433-3432-3432-243-231-12  
(0 / 1)

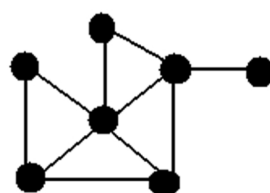

543322-45321-3543-3532-254-253-14  
(0 / 1)

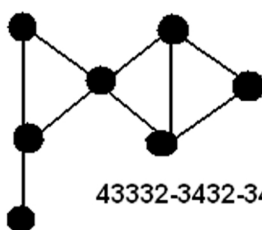

43332-3432-3432-3421-243-233-13  
(1 / 0)

Supplement: Additional file 7 — Figure S4. Motifs belonging to family f5. Network diagrams of motifs up to size 7 (nodes) belonging to family f5. Motif identifier for each motif is displayed below the motif with the number of members for ASCN and APCN respectively in parentheses separated by a front slash. [file 1471-2105-12-195-S7.PDF]

f6a:

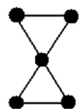

42222-242-242-242-242  
(1 / 3)

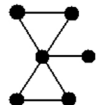

522221-252-252-252-252-15  
(1 / 3)

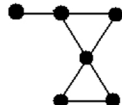

43222-3421-243-242-242-13  
(1 / 1)

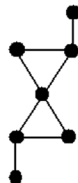

43322-3421-3421-243-243-13-13  
(4 / 3)

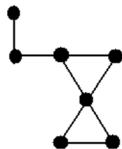

43222-3422-243-242-242-231-12  
(0 / 1)

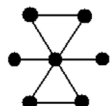

6222211-262-262-262-262-16-16  
(0 / 1)

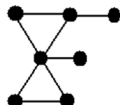

532221-3521-253-252-252-15-13  
(0 / 1)

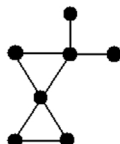

44222-44211-244-242-242-14-14  
(0 / 2)

f6b:

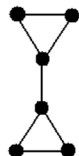

3322-3322-232-232-232-232  
(1 / 1)

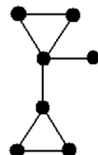

43221-3422-242-242-232-232-14  
(0 / 1)

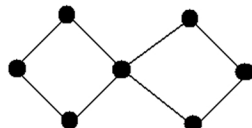

42222-242-242-242-242-222-222  
(1 / 1)

f7:

Supplement: Additional file 8 — Figure S5. Motifs belonging to families f6a, f6b and f7. Network diagrams of motifs up to size 7 (nodes) belonging to family f6a (left panel), f6b (middle) and f7 (right). Motif identifier for each motif is displayed below the motif with the number of members for ASCN and APCN respectively in parentheses separated by a front slash. [file 1471-2105-12-195-S8.PDF]

f8a:

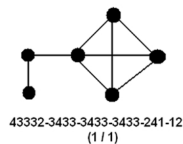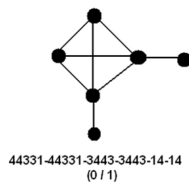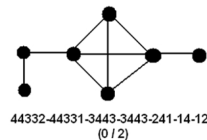

f8b:

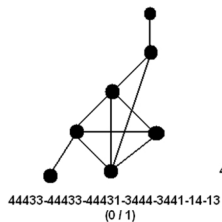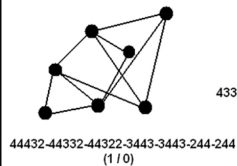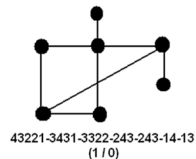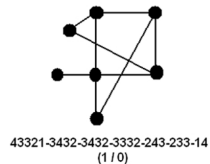

f8c:

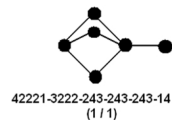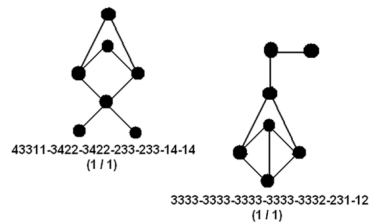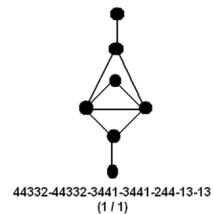

Supplement: Additional file 9 — Figure S6. Motifs belonging to families f8a, f8b and f8c. Network diagrams of motifs up to size 7 (nodes) belonging to family f8a (left panel), f8b (middle) and f8c (right). Motif identifier for each motif is displayed below the motif with the number of members for ASCN and APCN respectively in parentheses separated by a front slash. [file 1471-2105-12-195-S9.PDF]

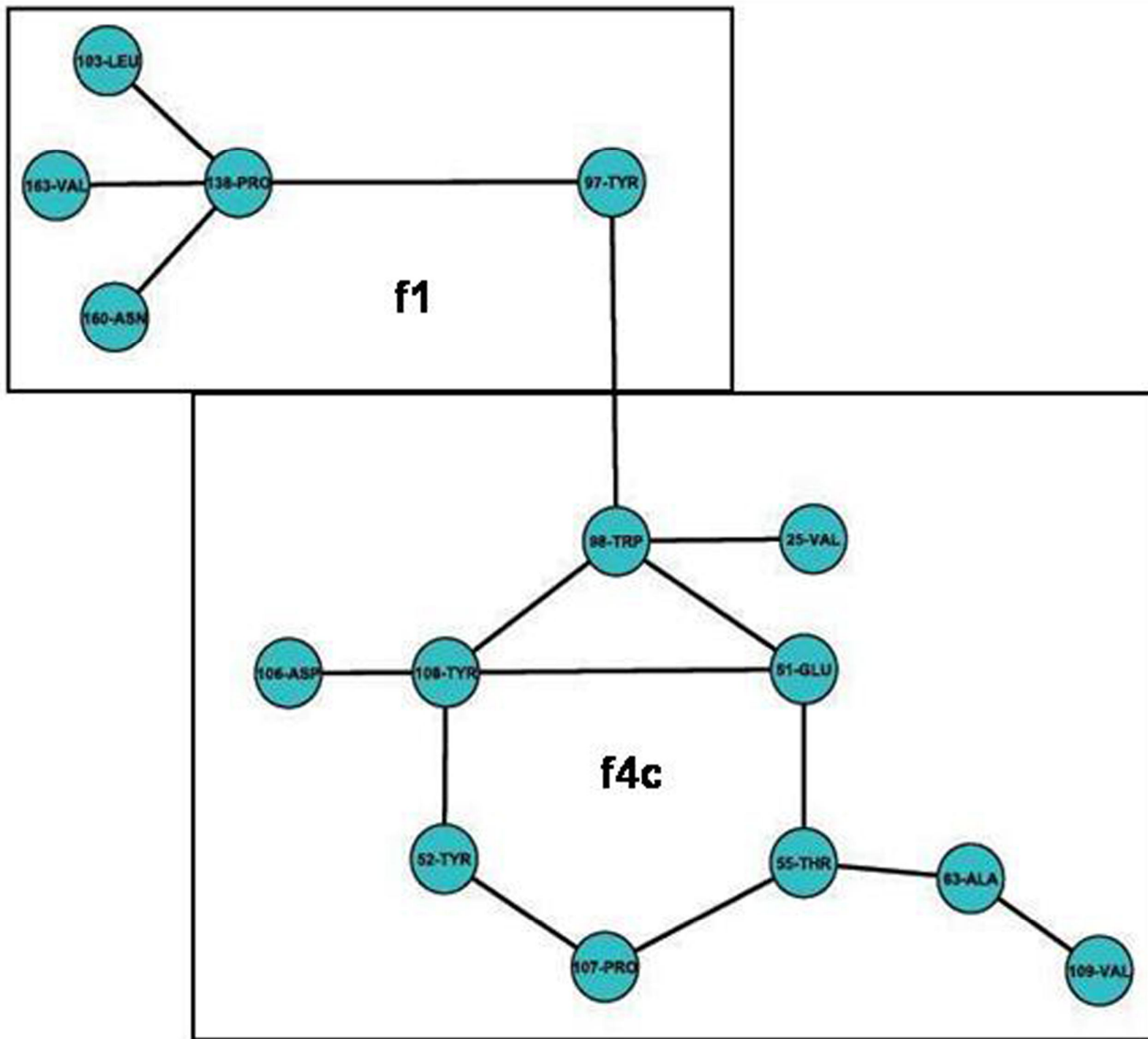

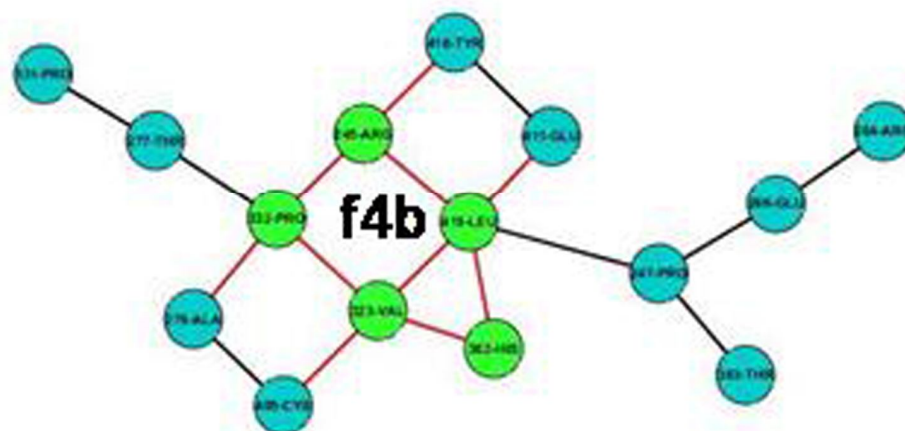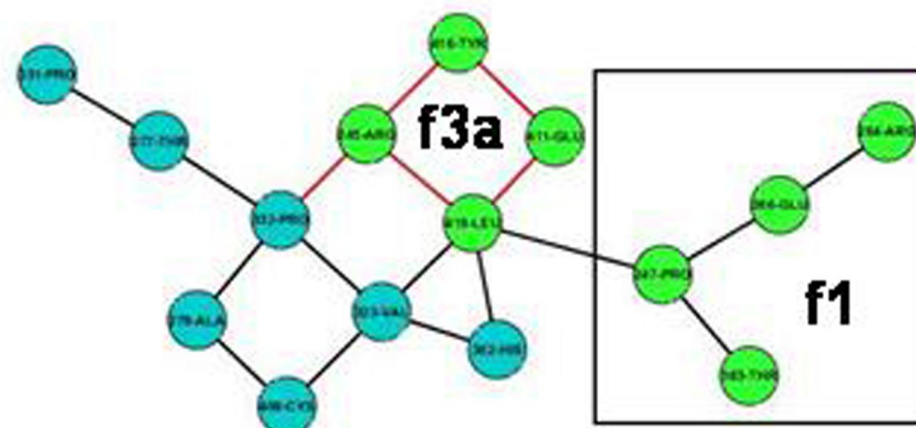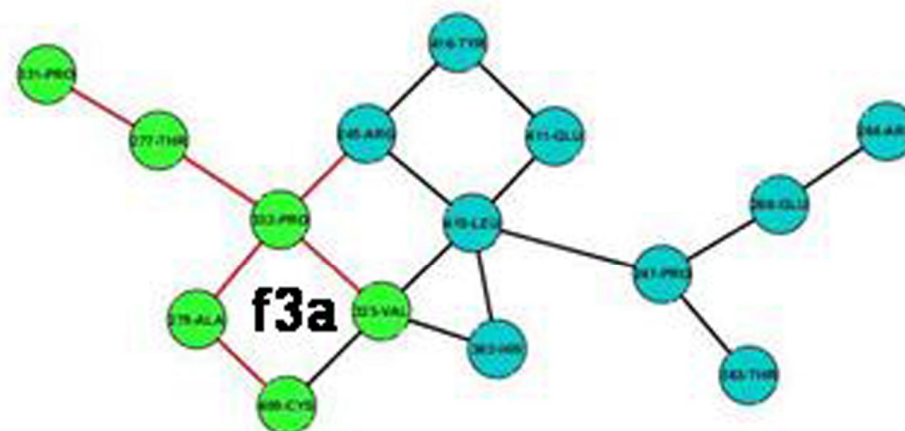

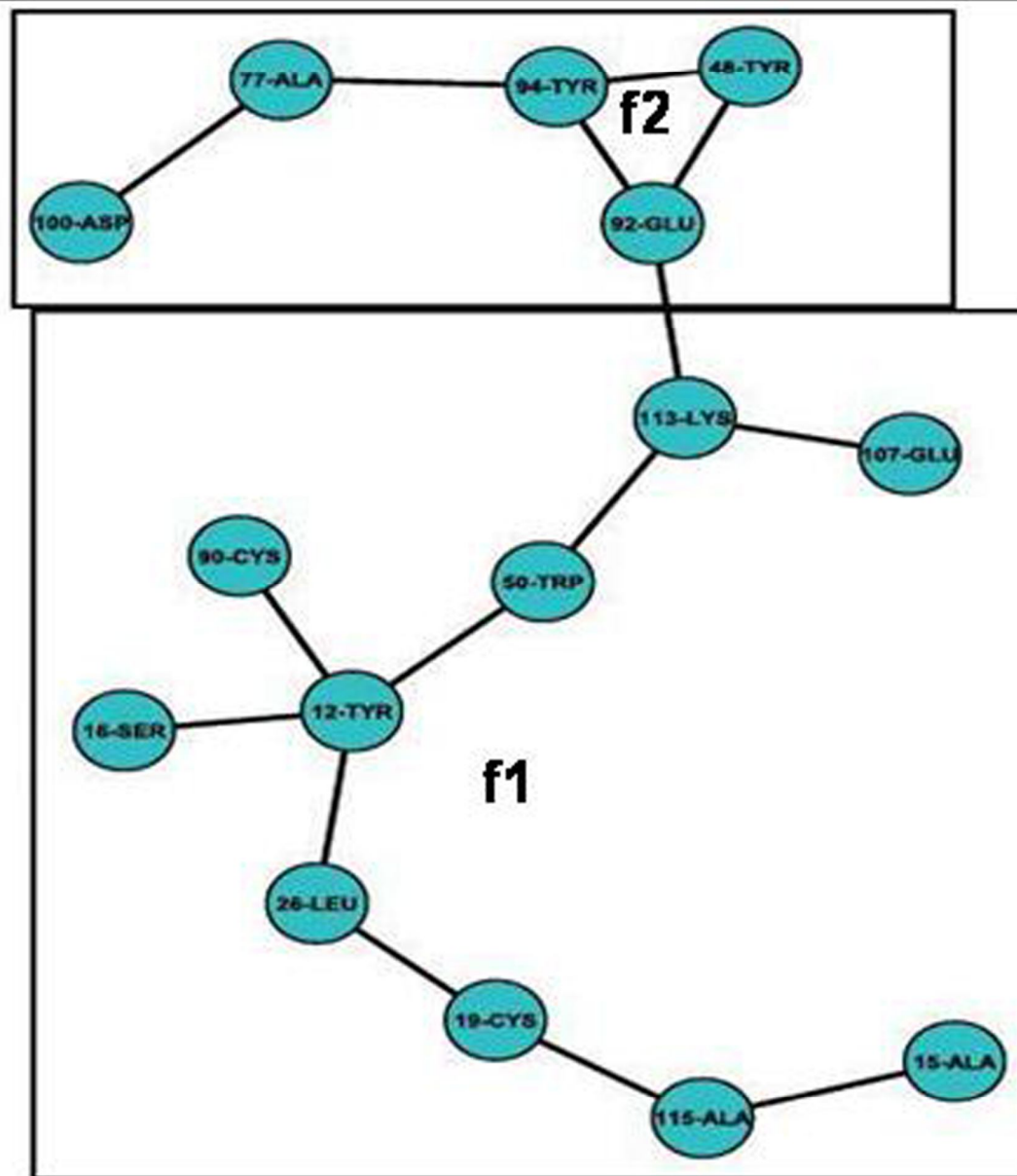

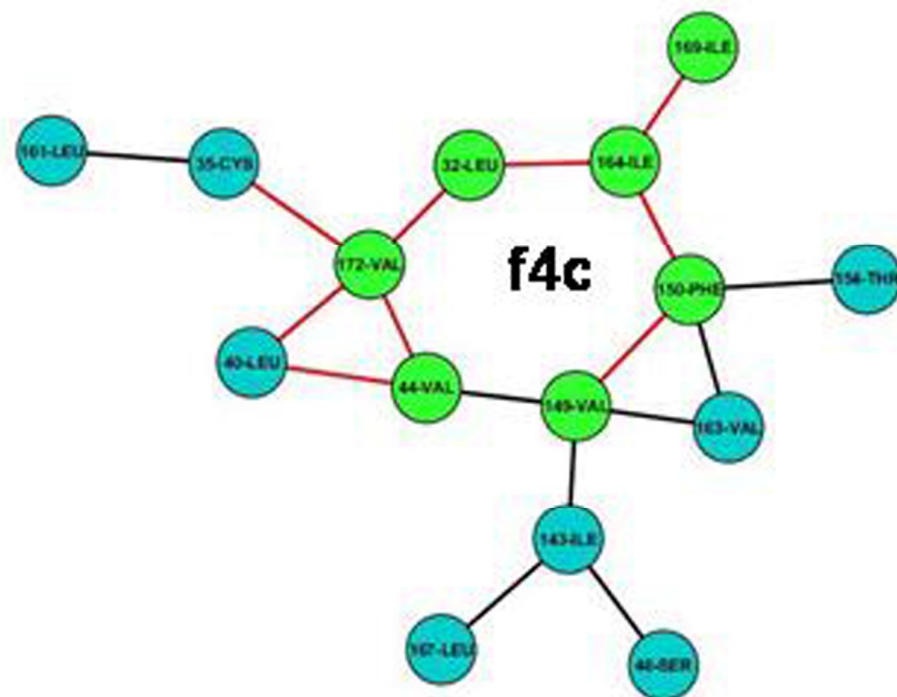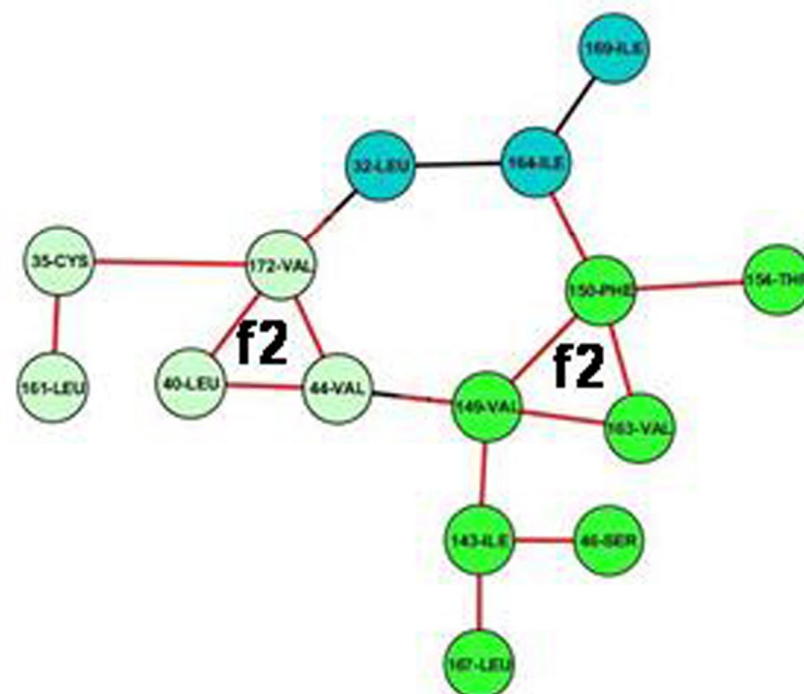

1G8L\_004

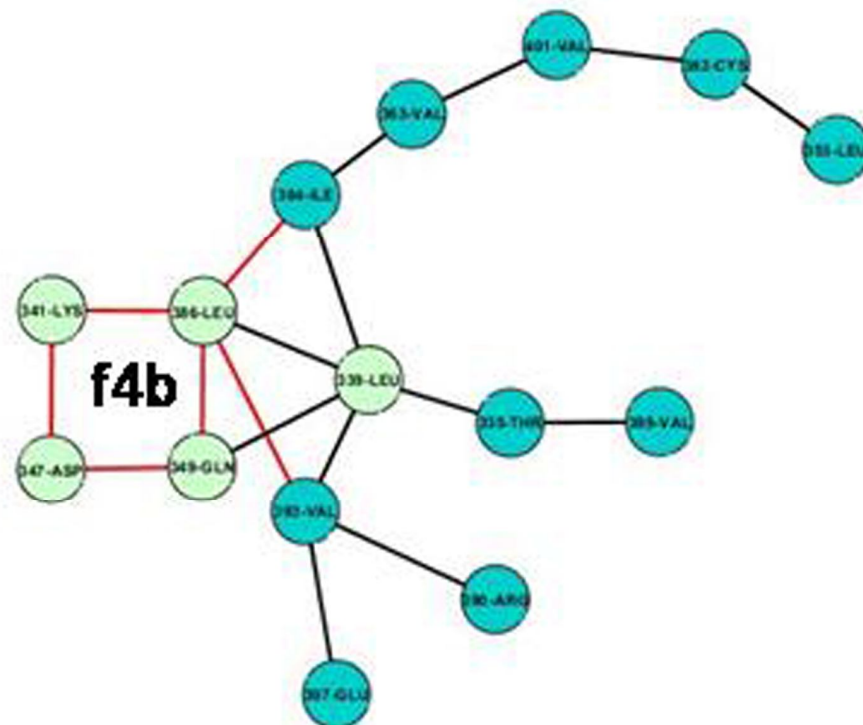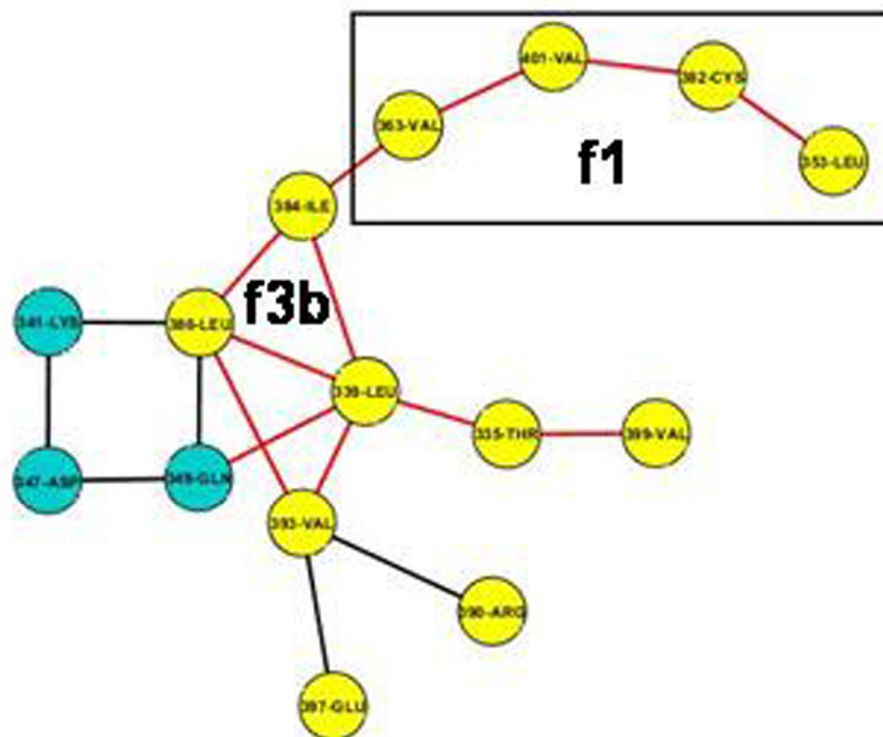

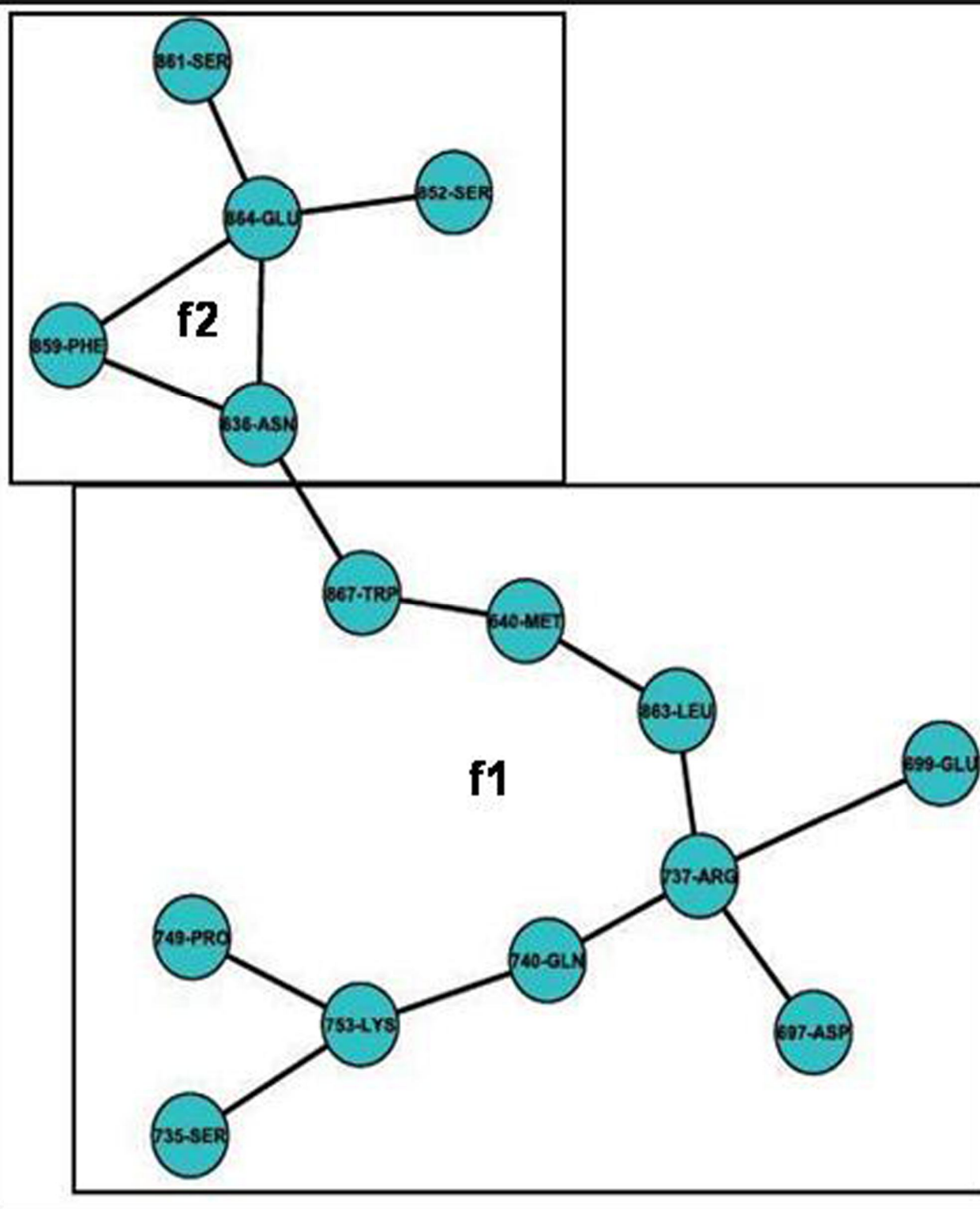

1HYO\_006

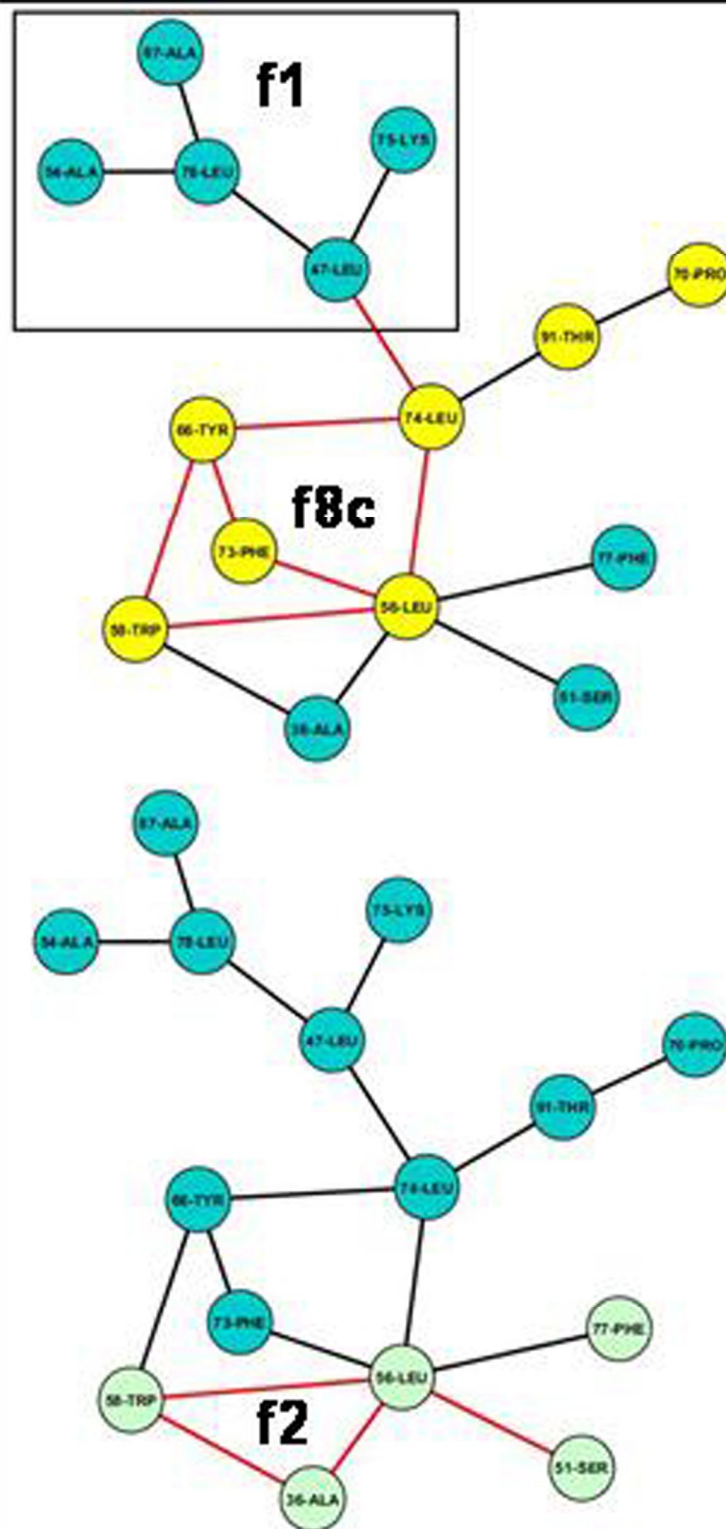

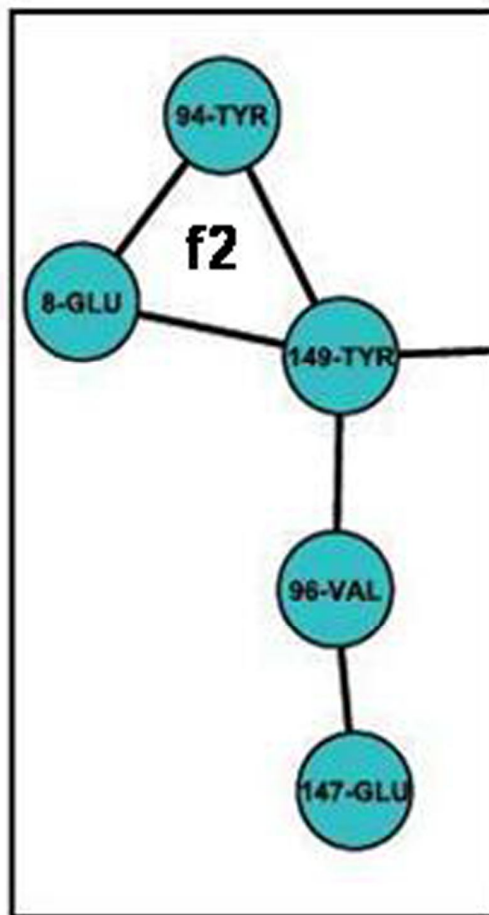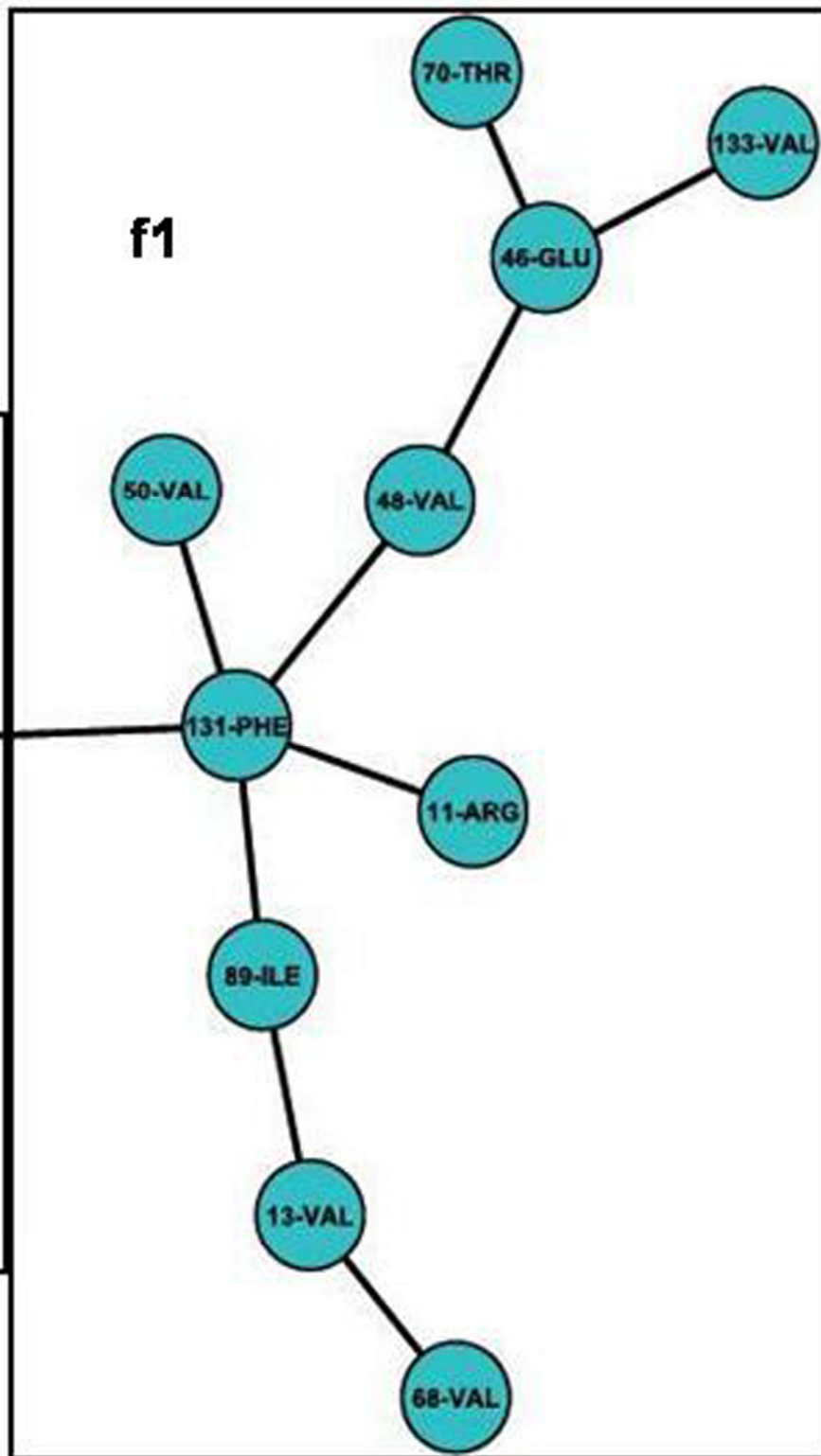

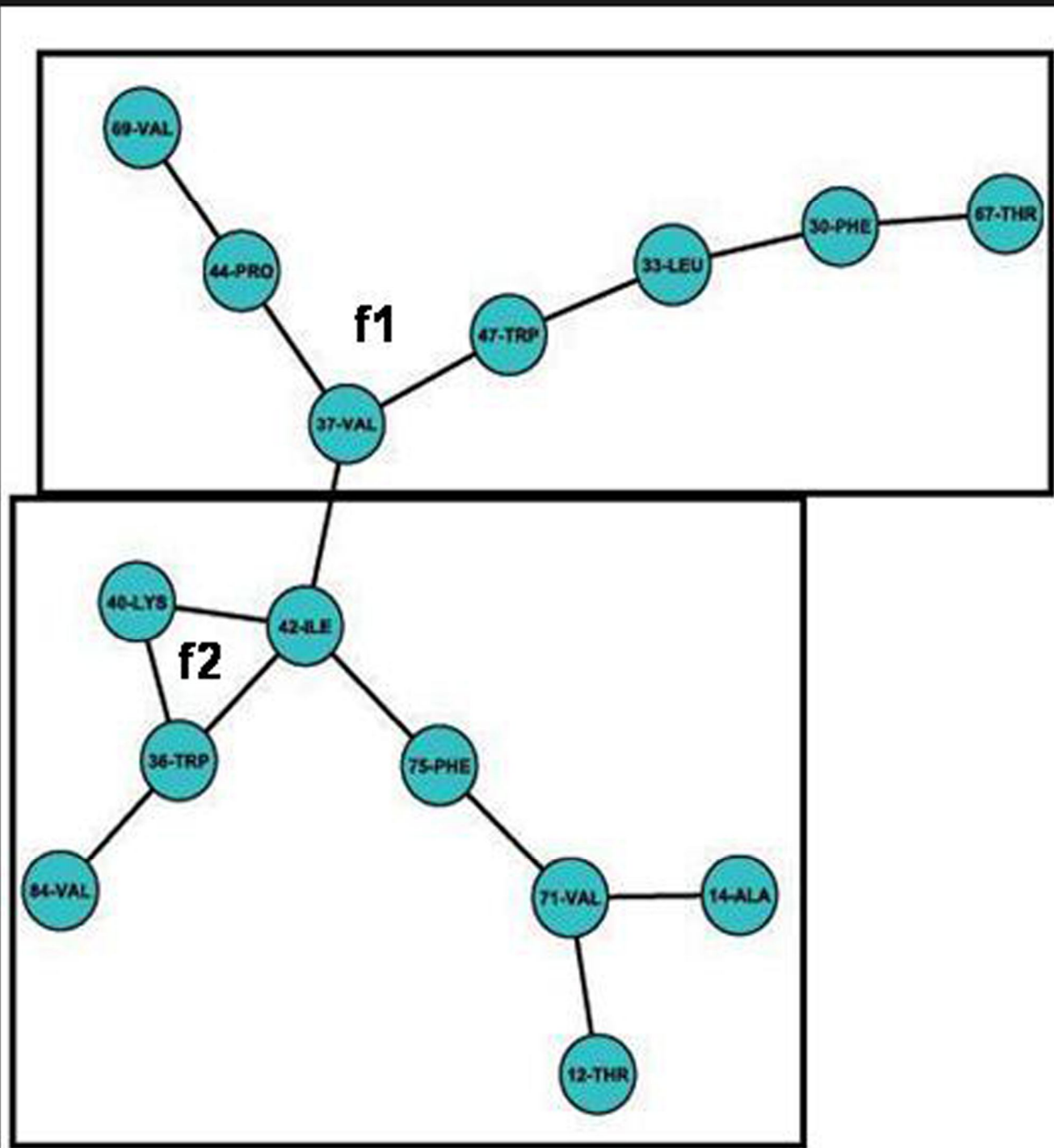

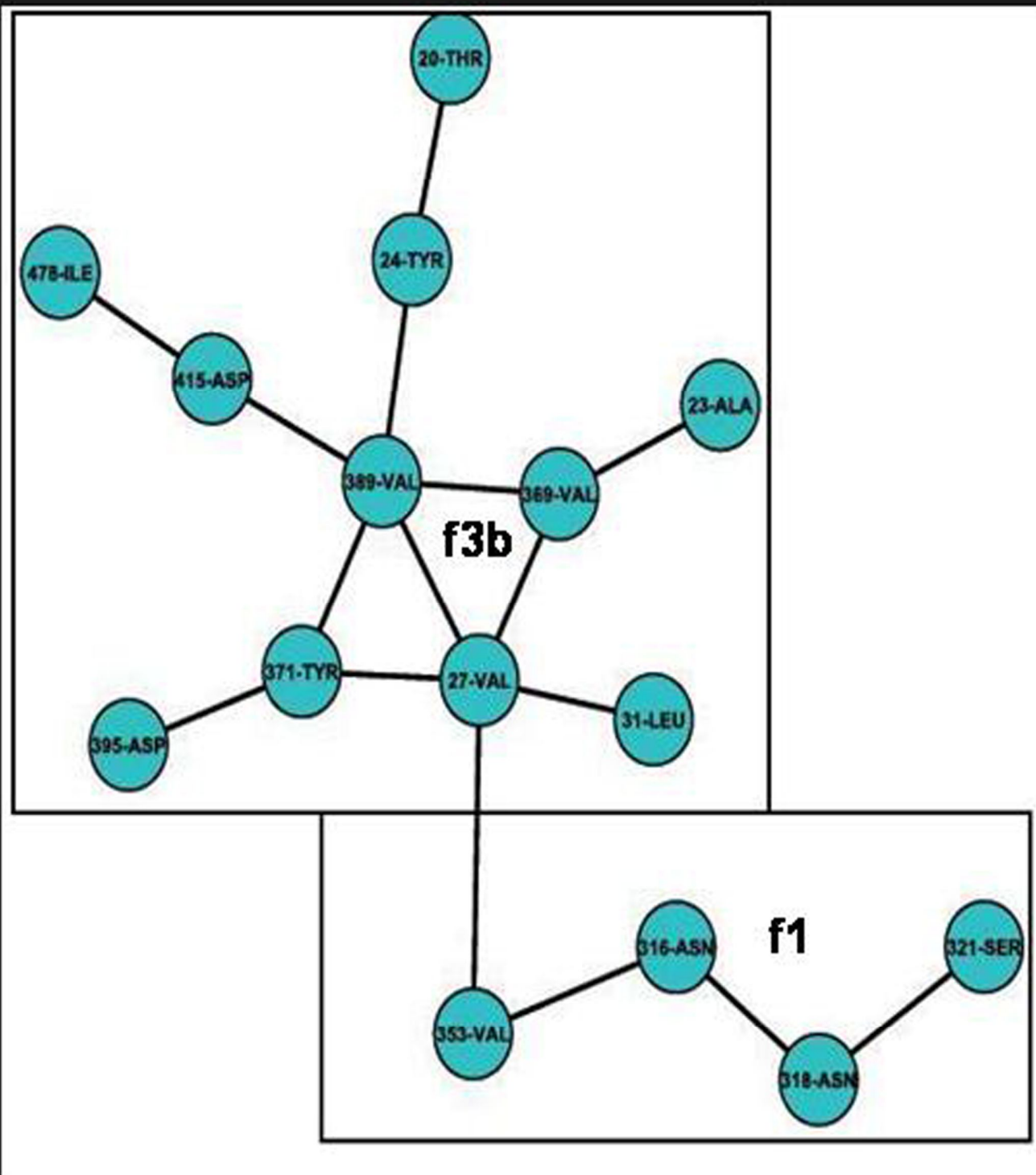

1K7I\_010

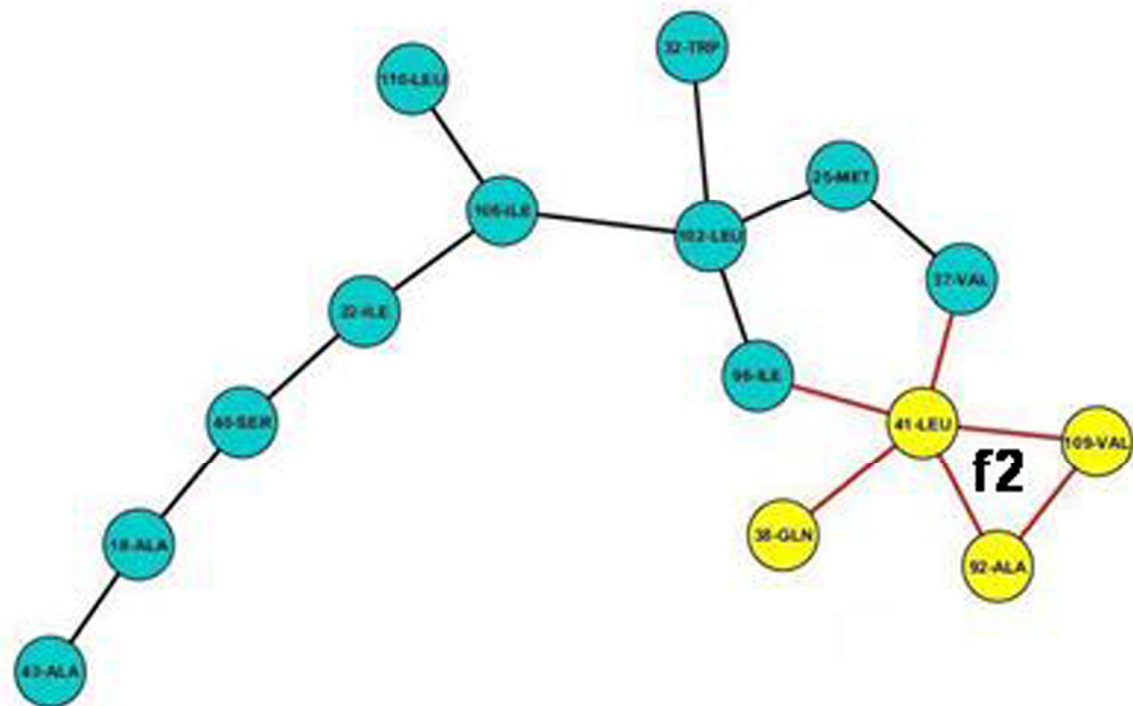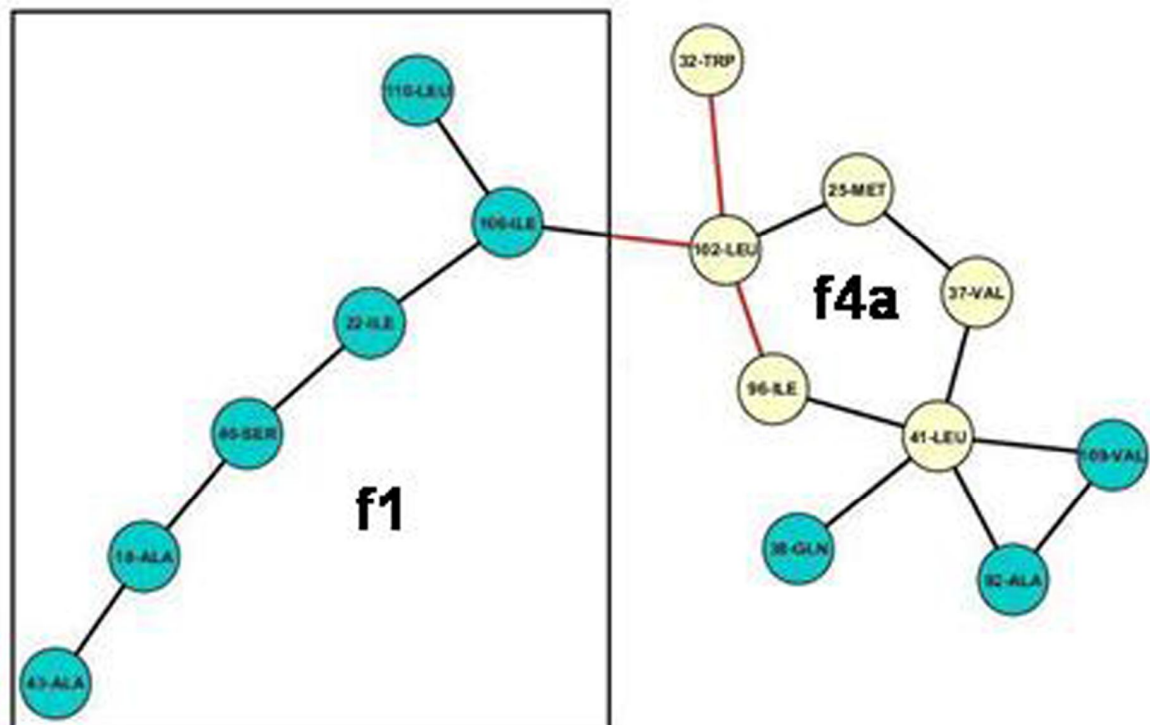

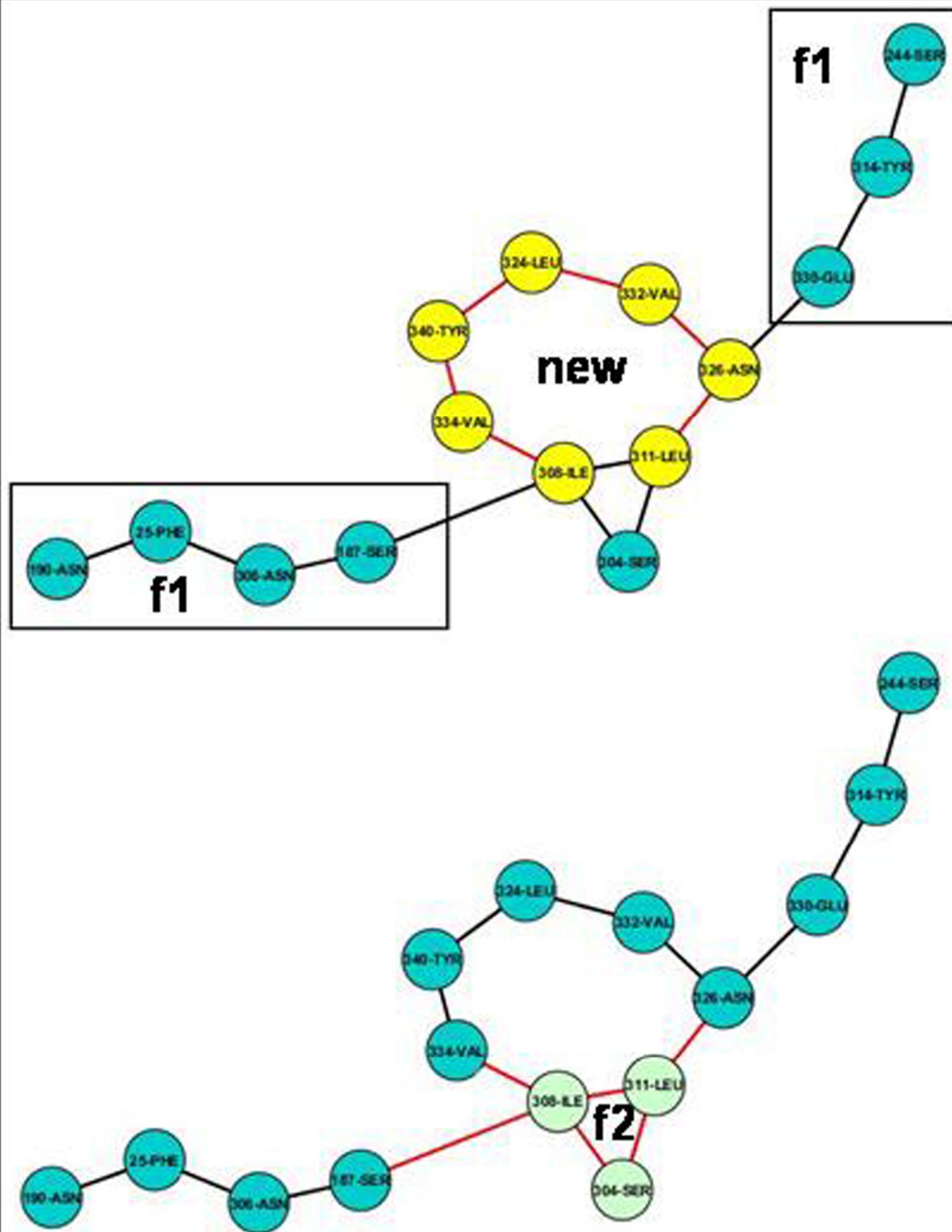

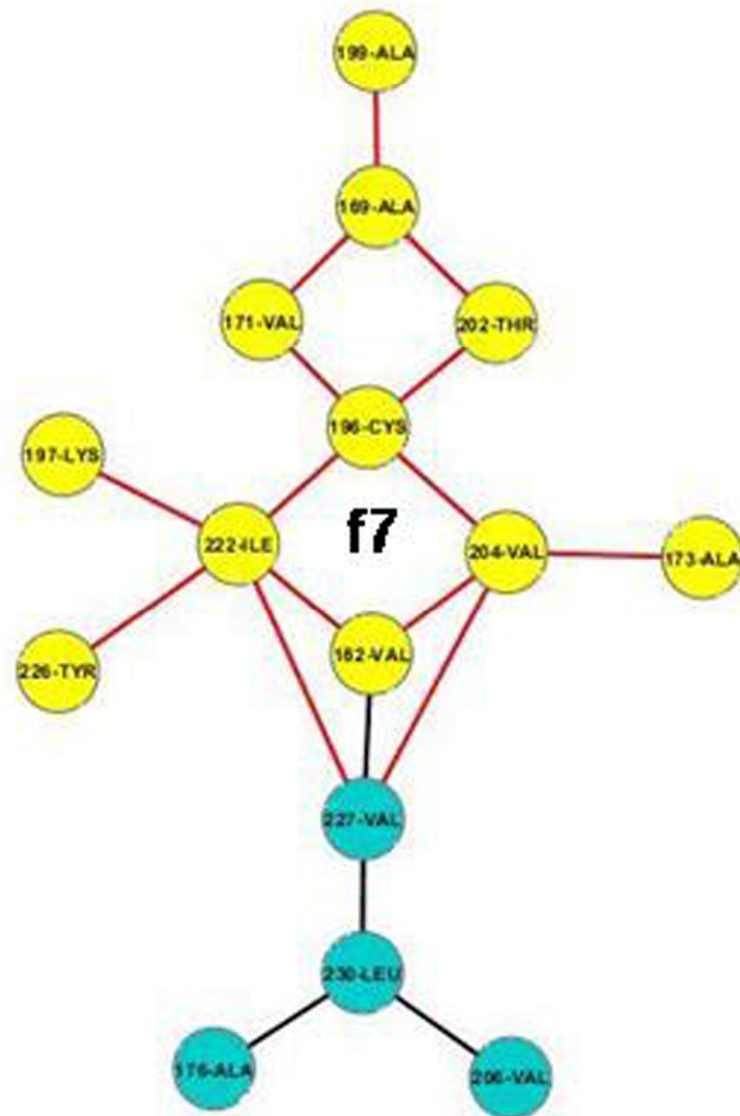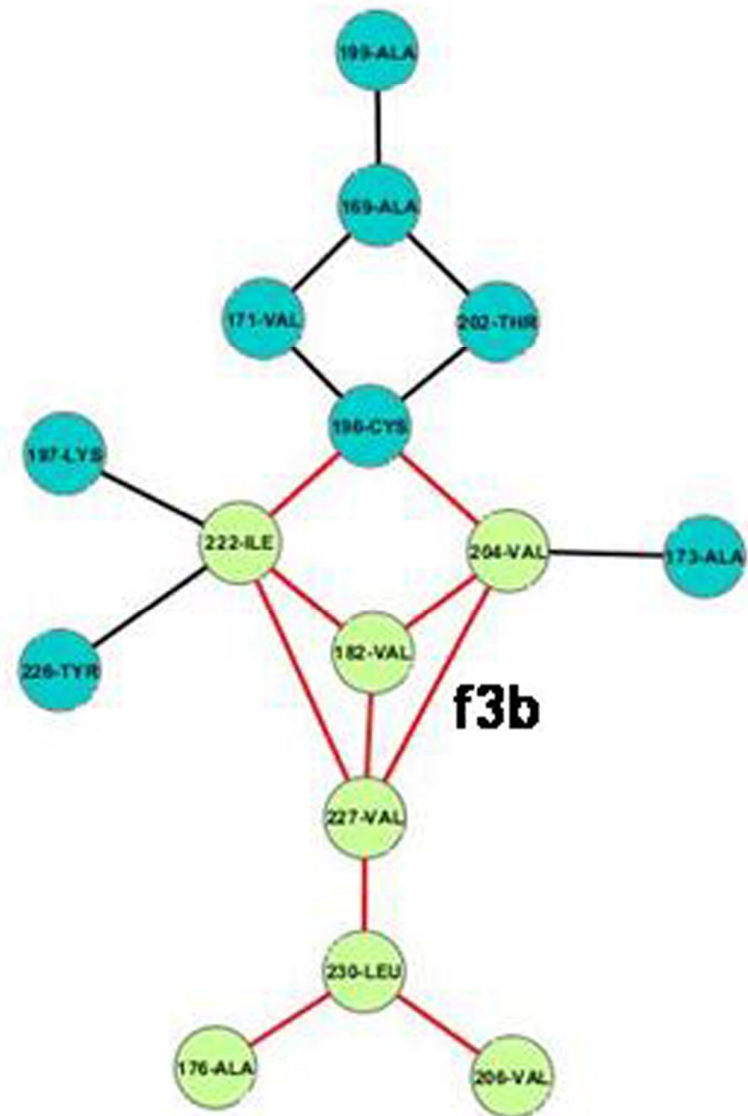

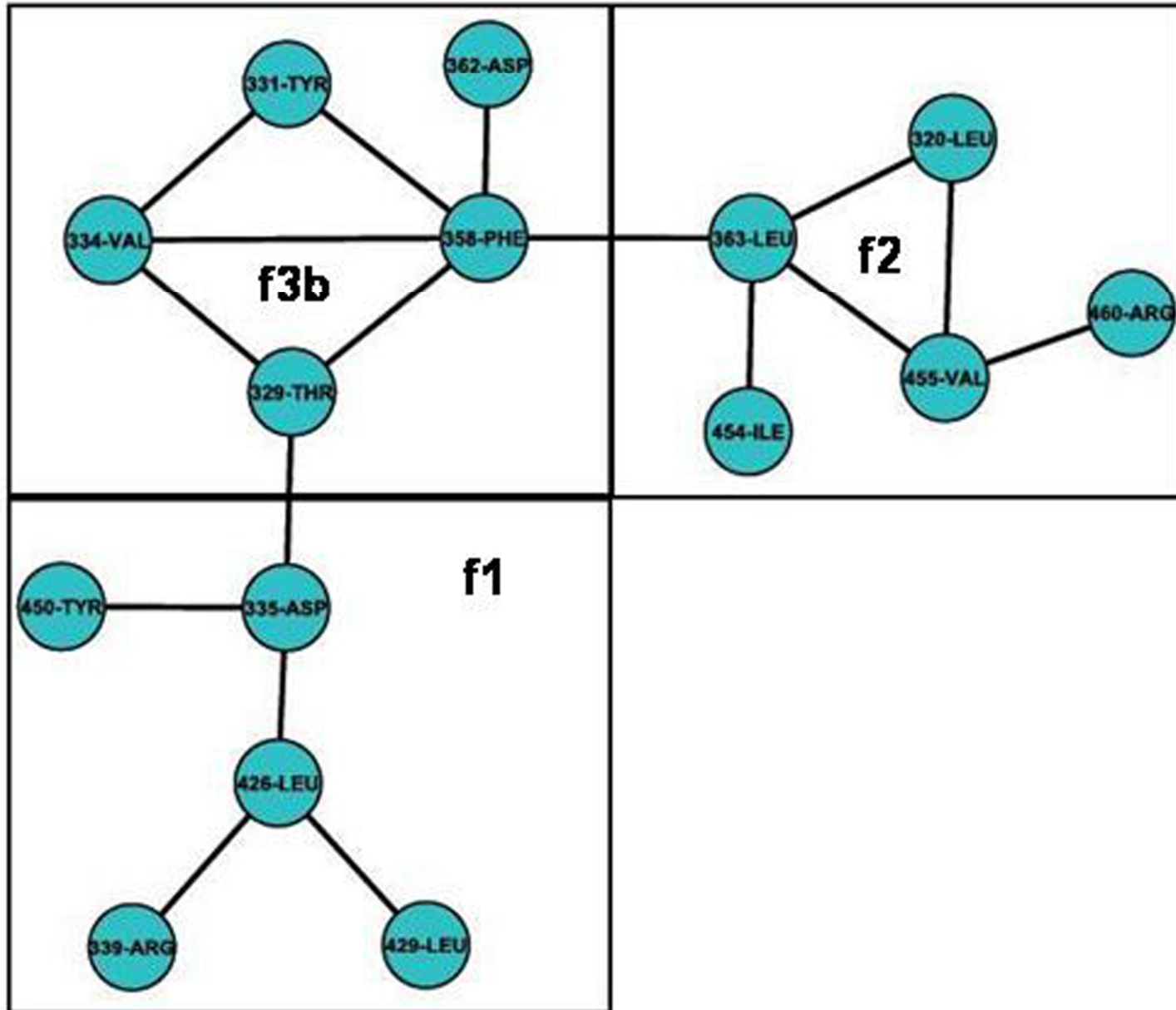

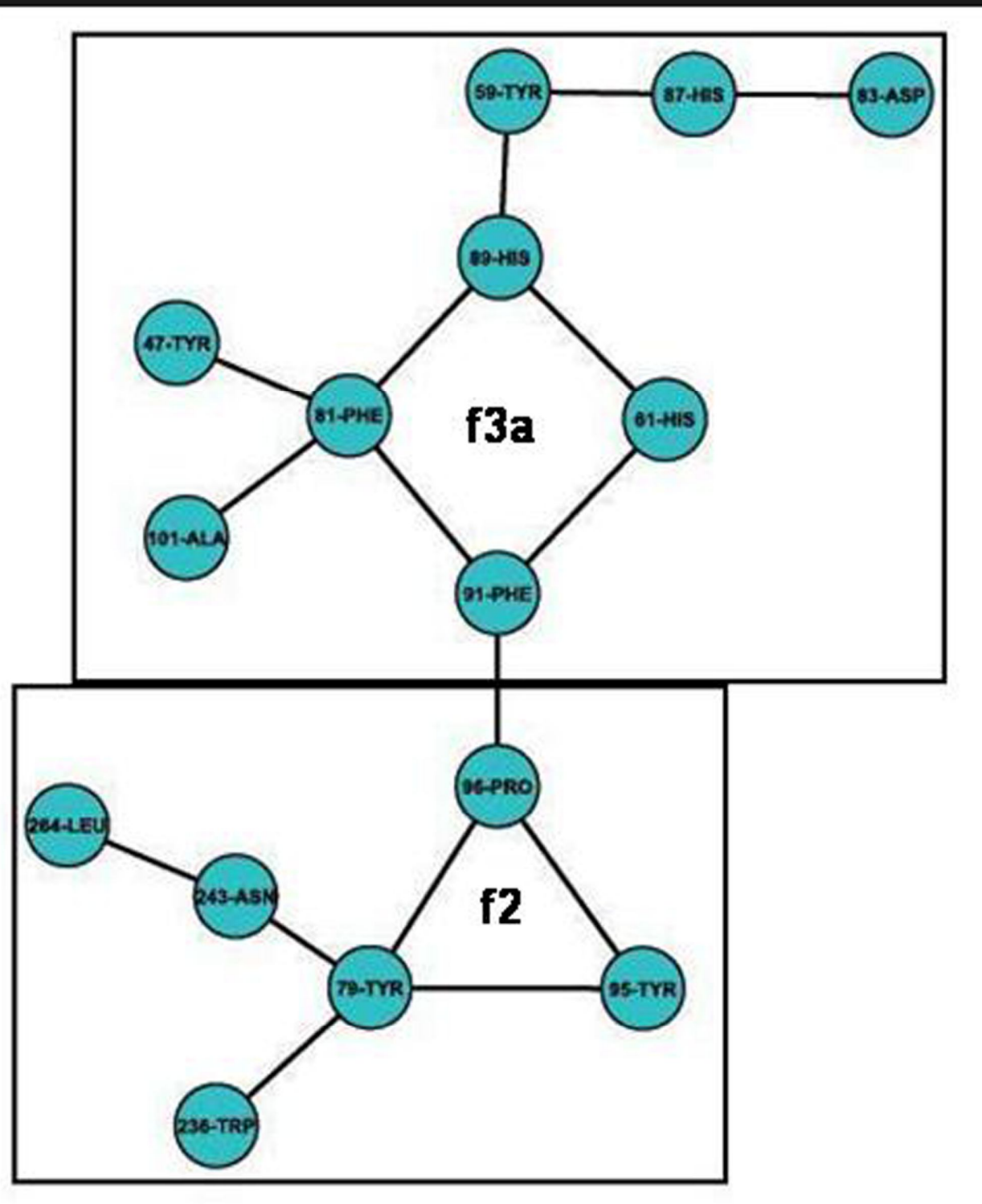

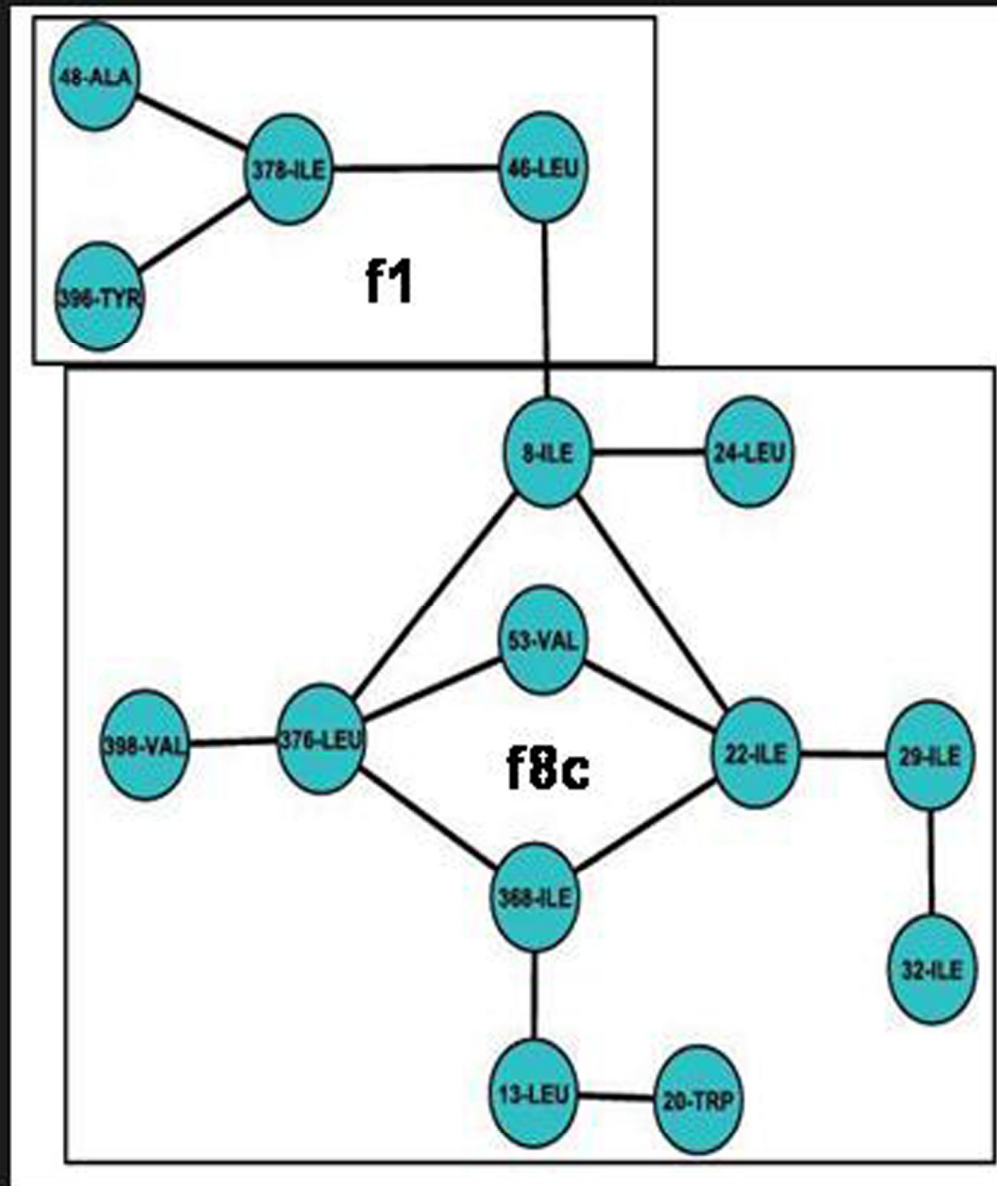

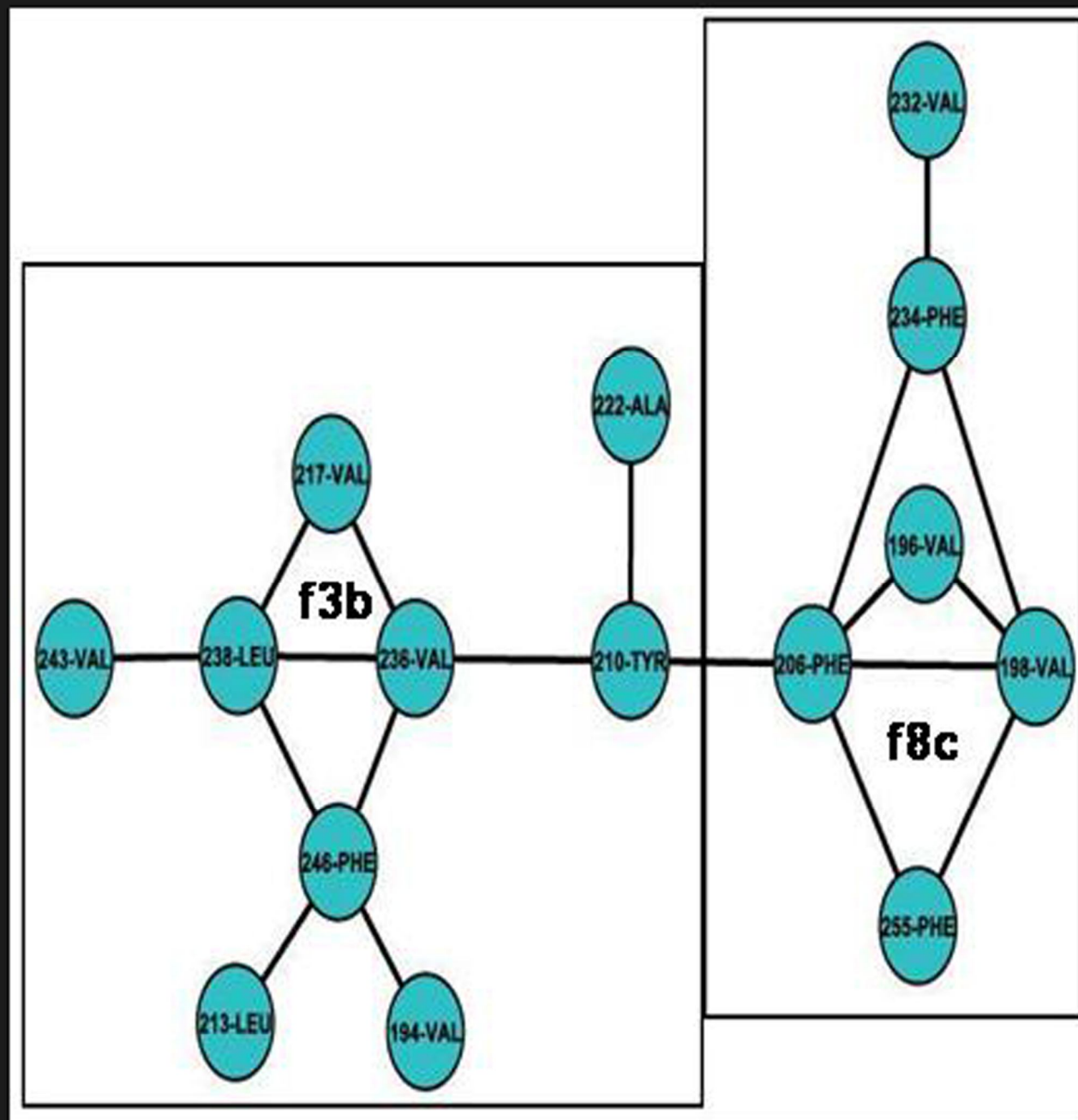

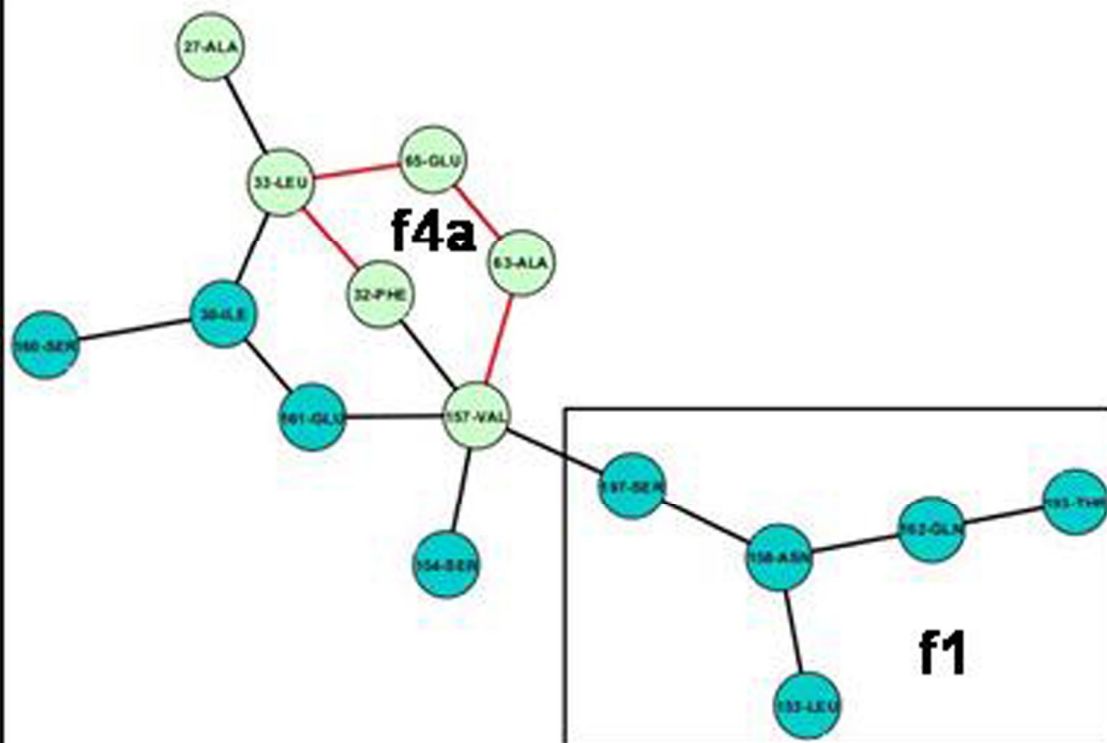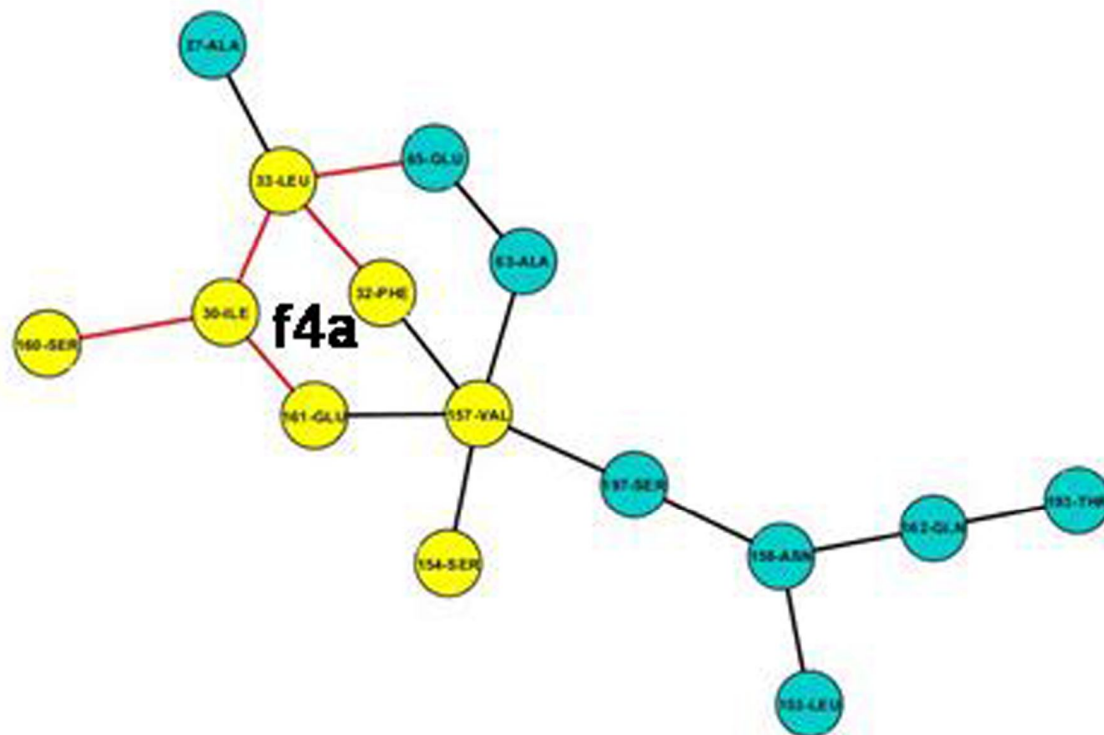

1U7L\_018

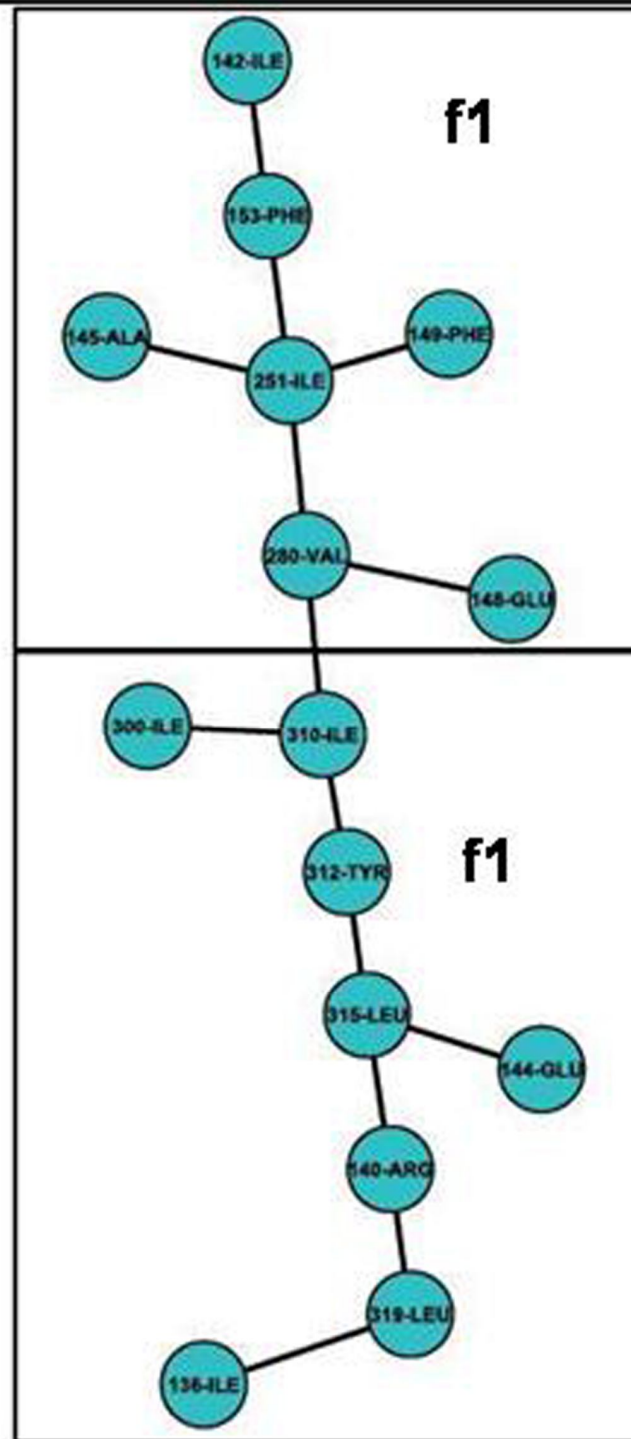

1X13\_019

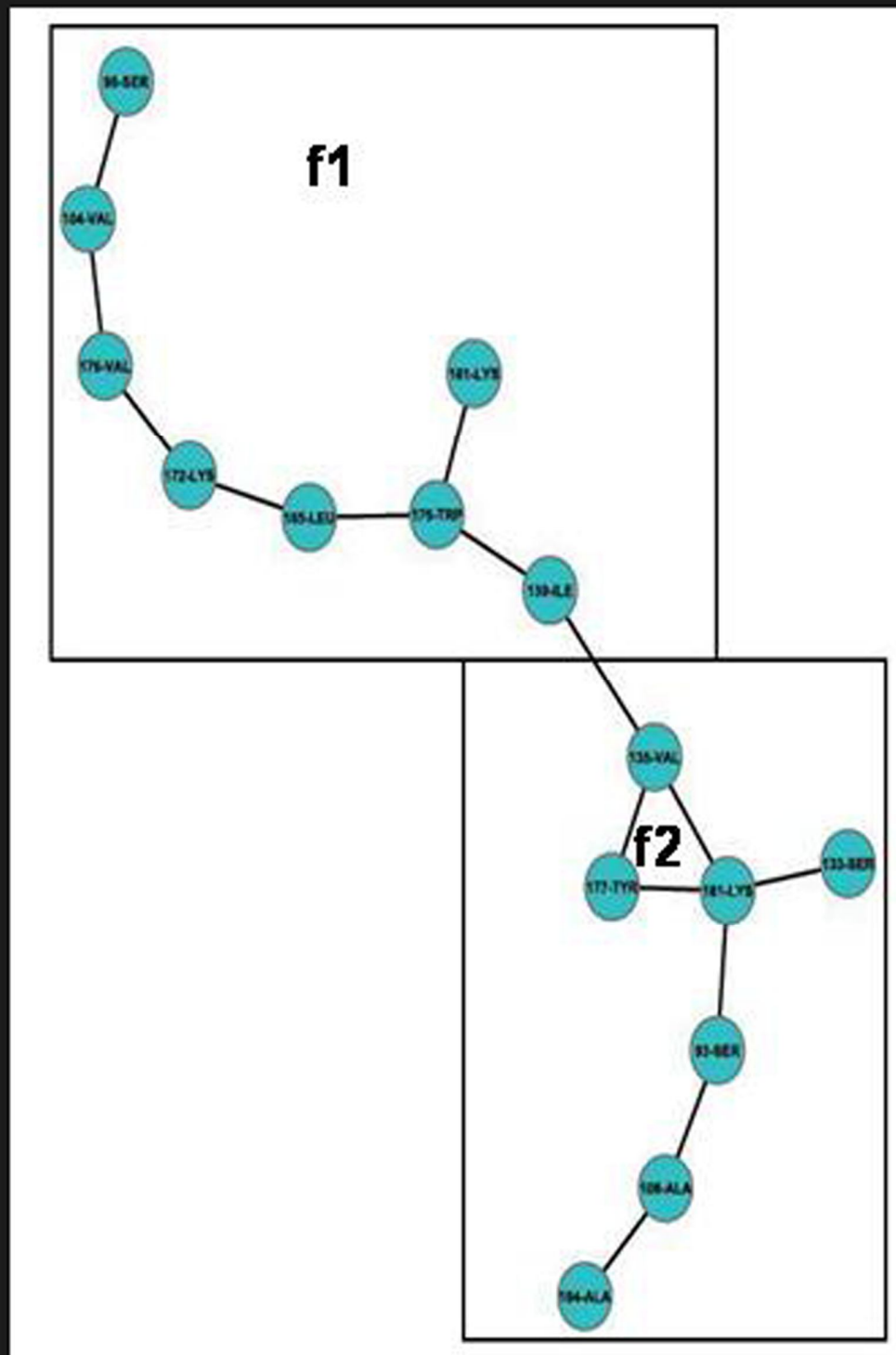

1Y1P\_020

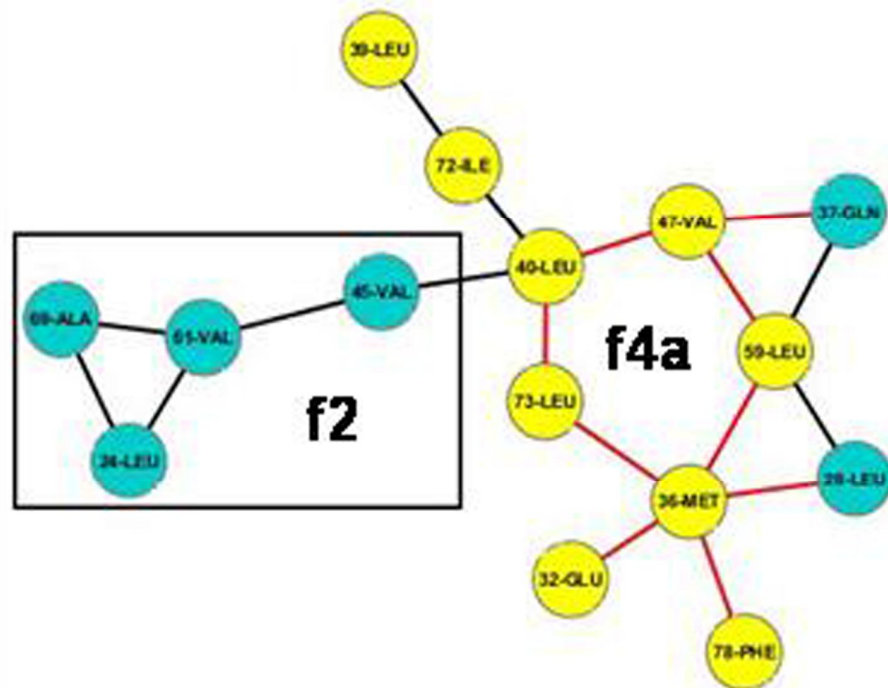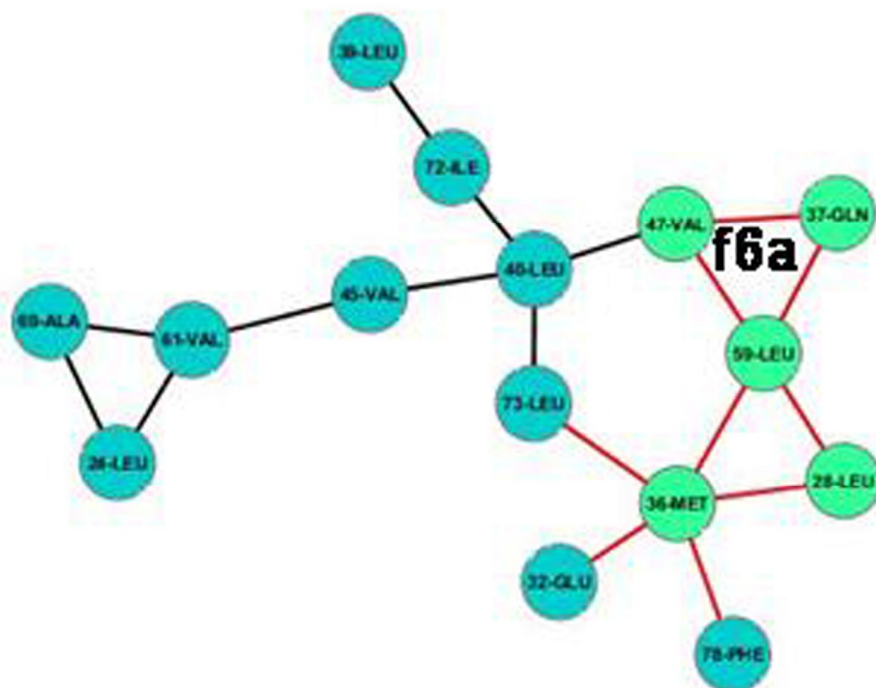

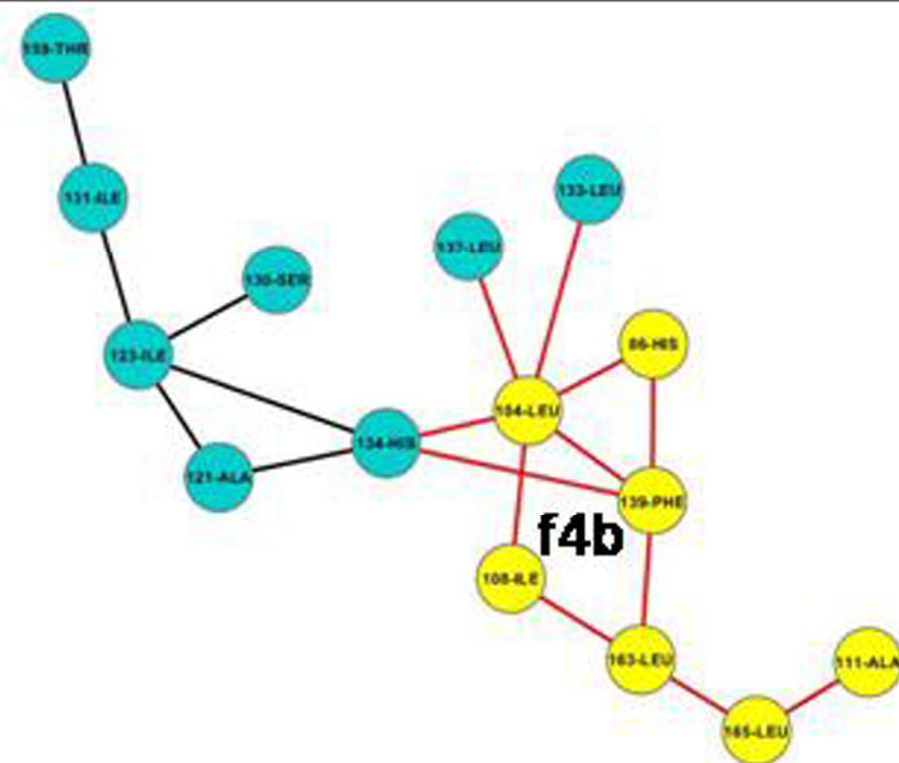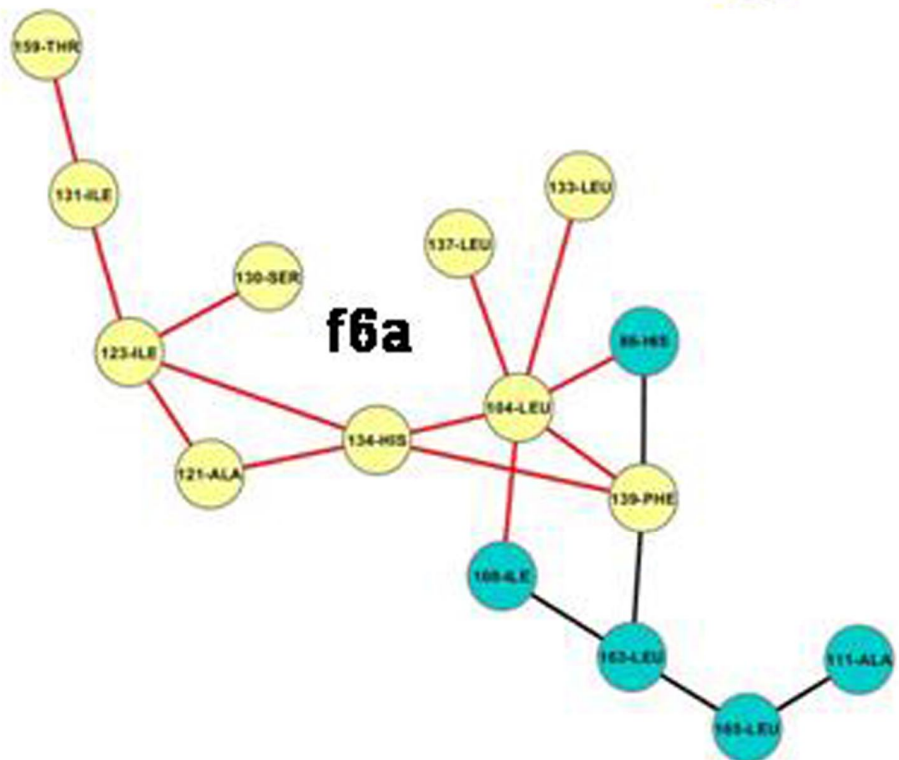

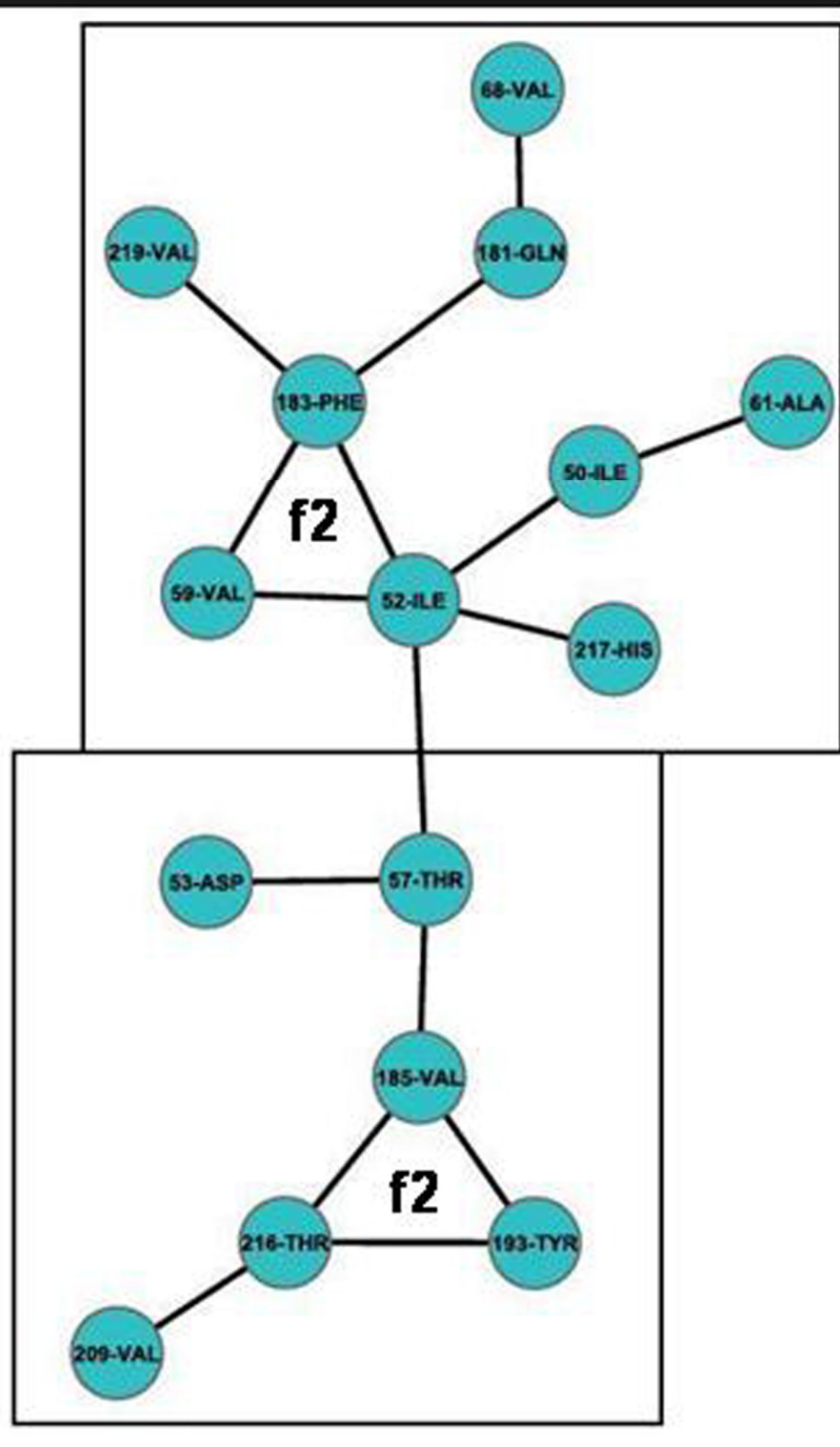

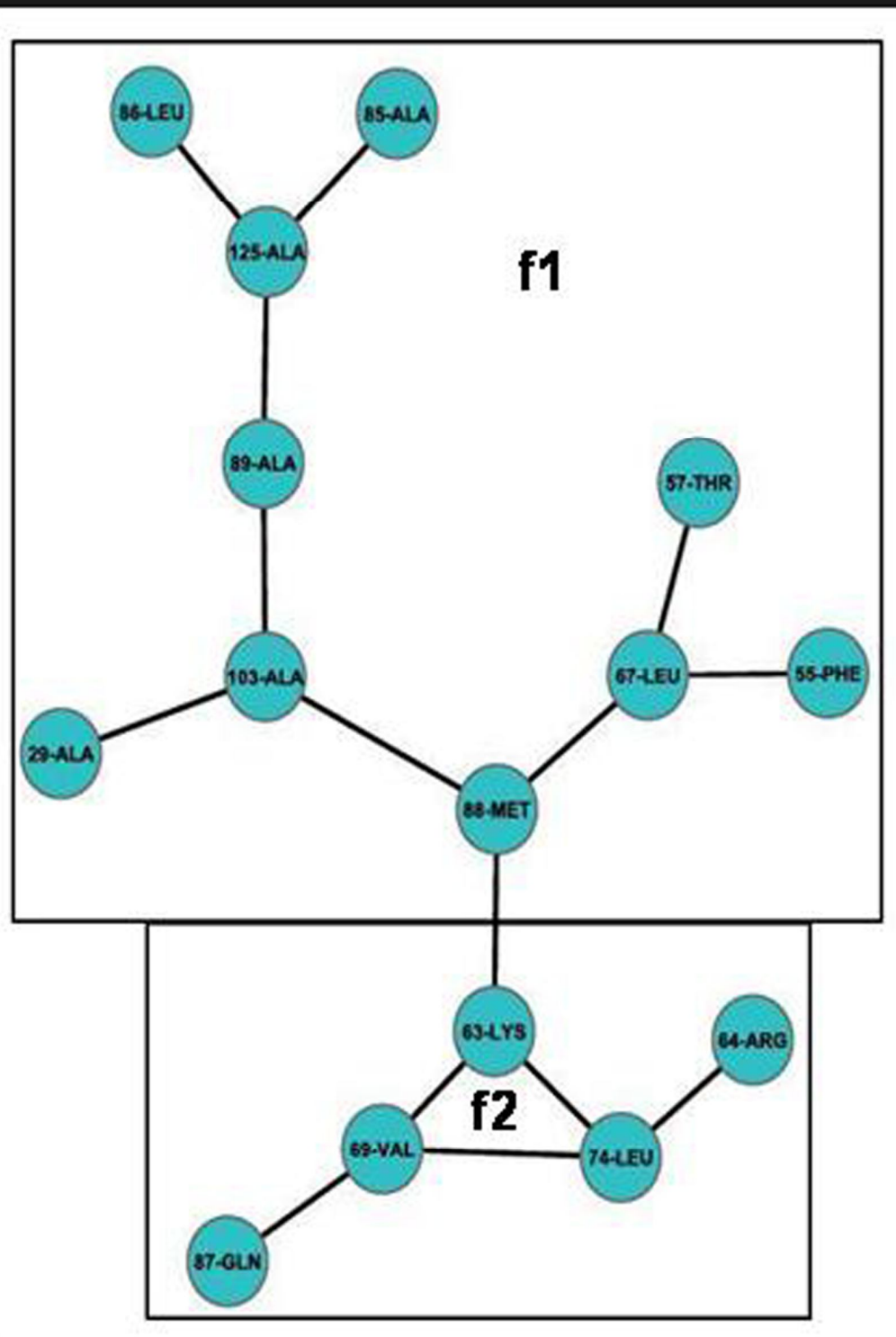

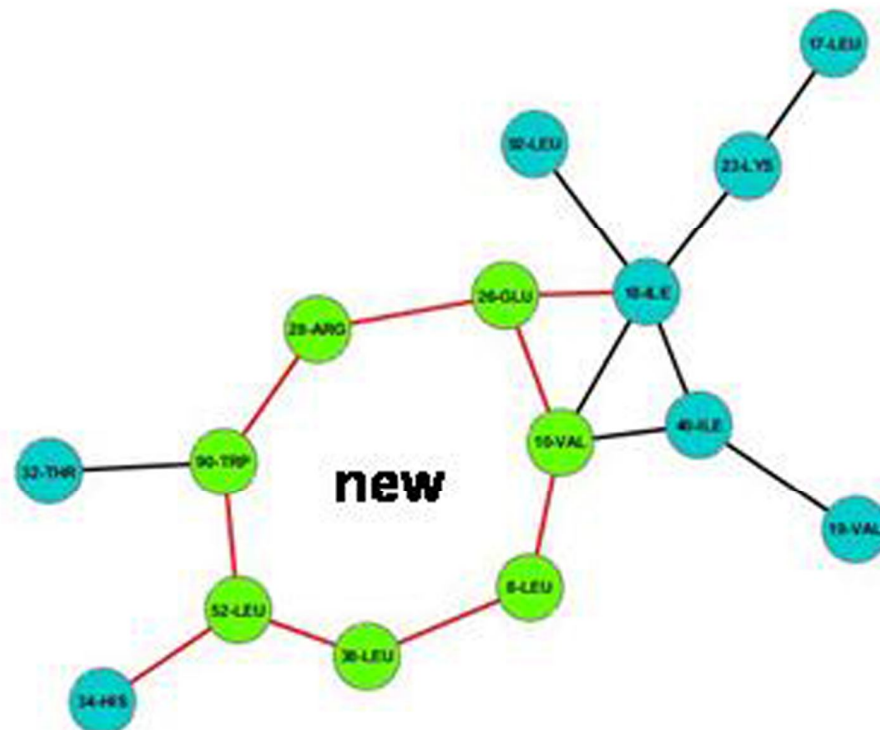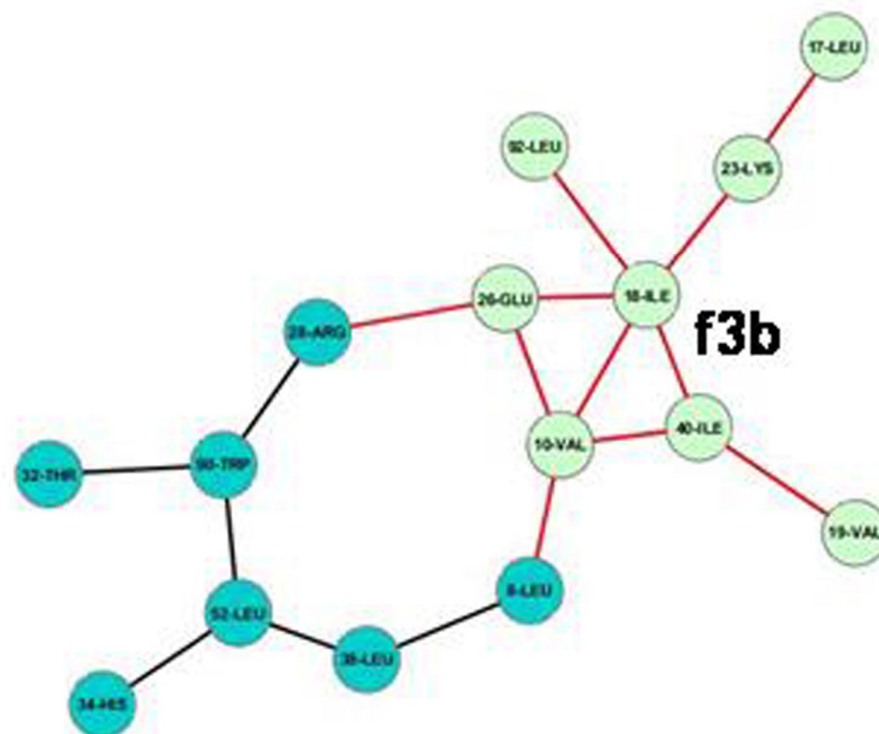

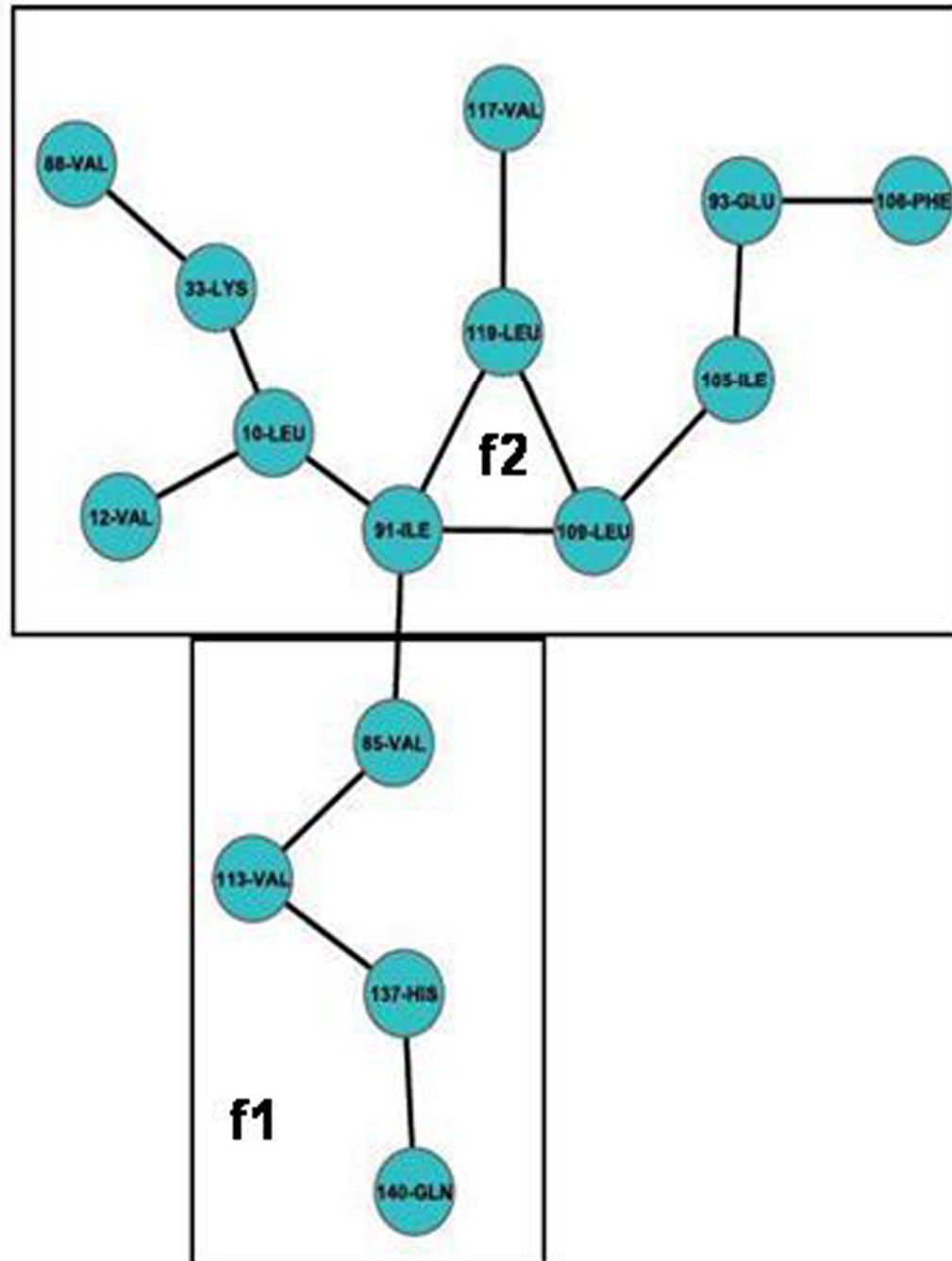

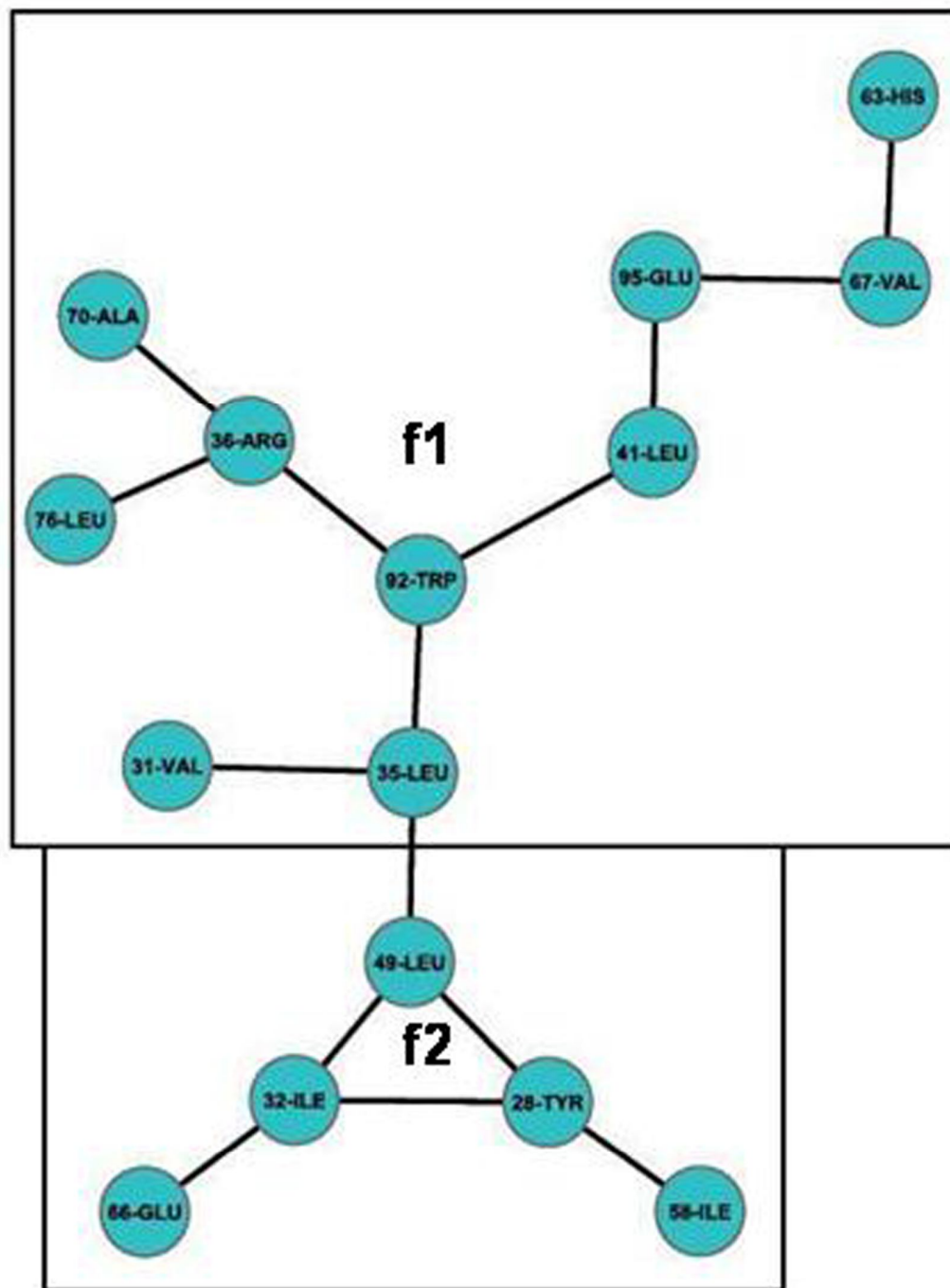

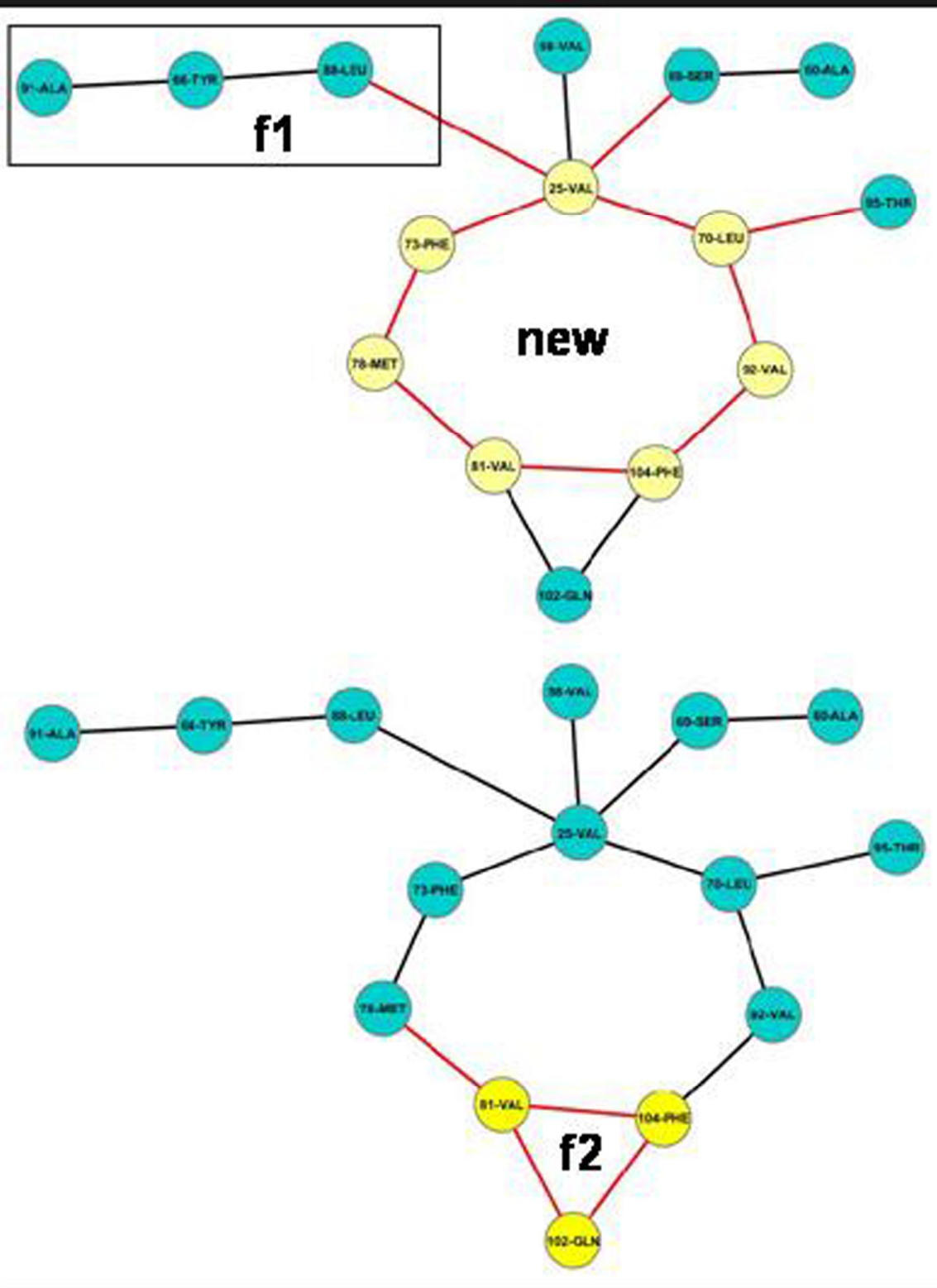

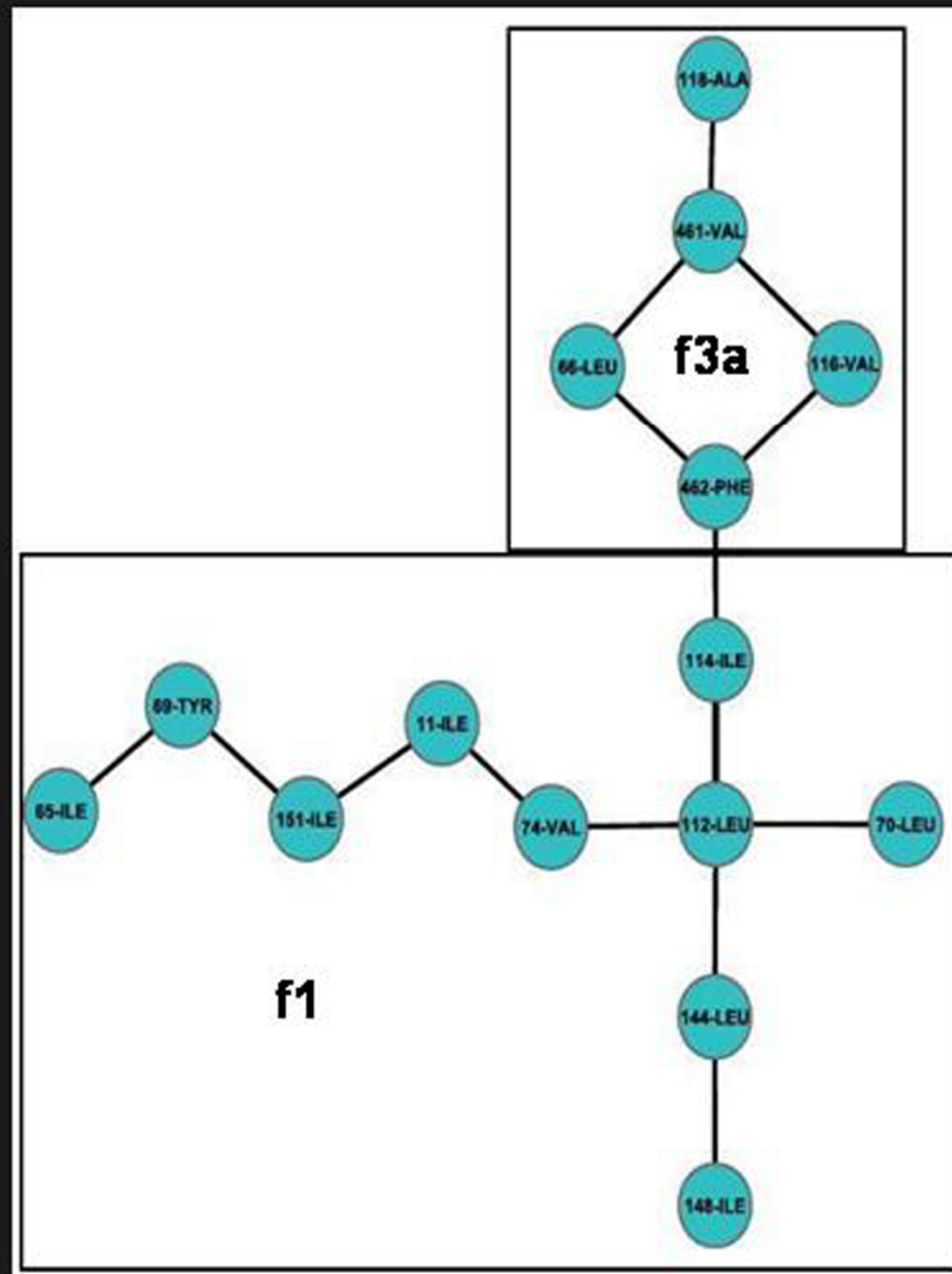

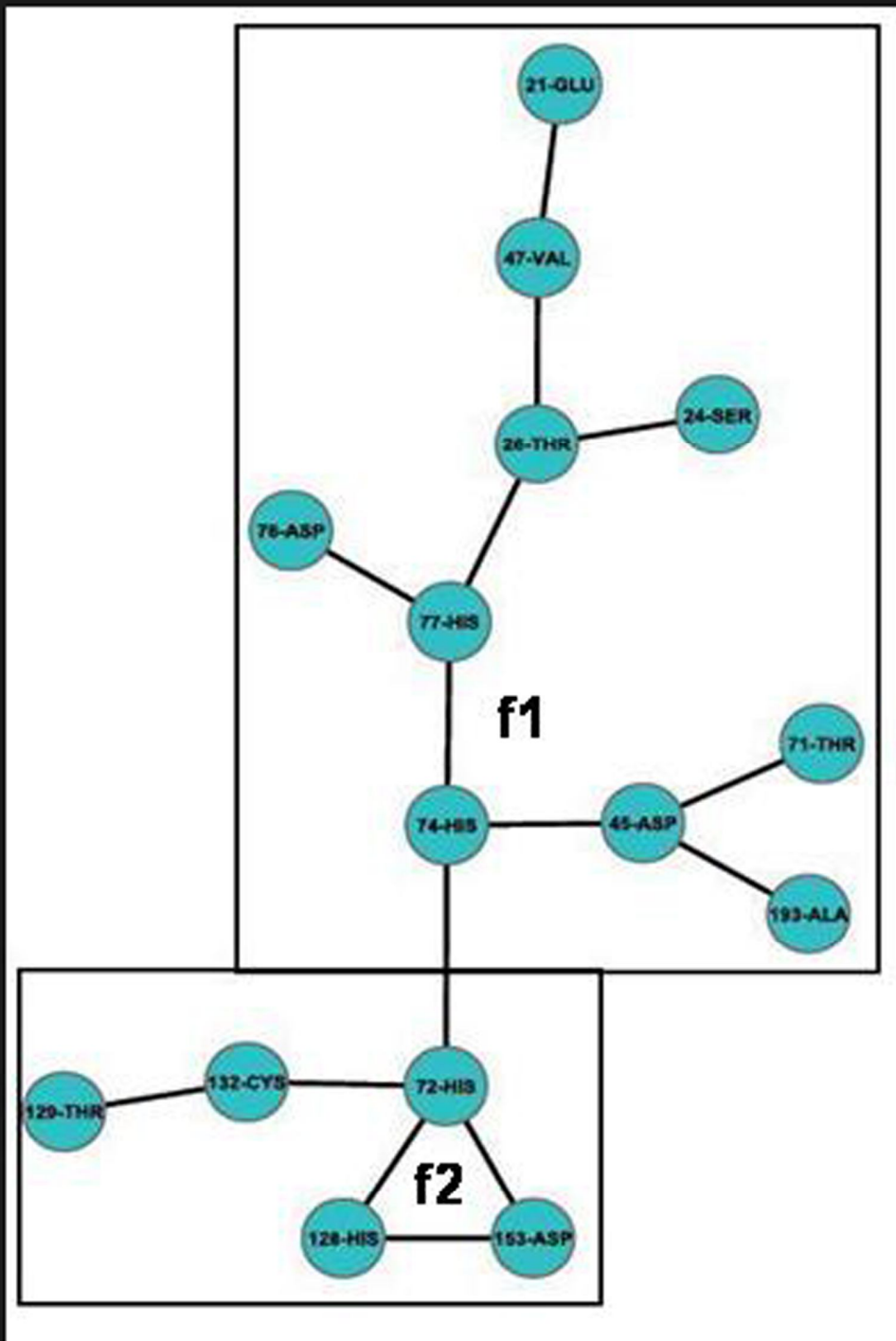

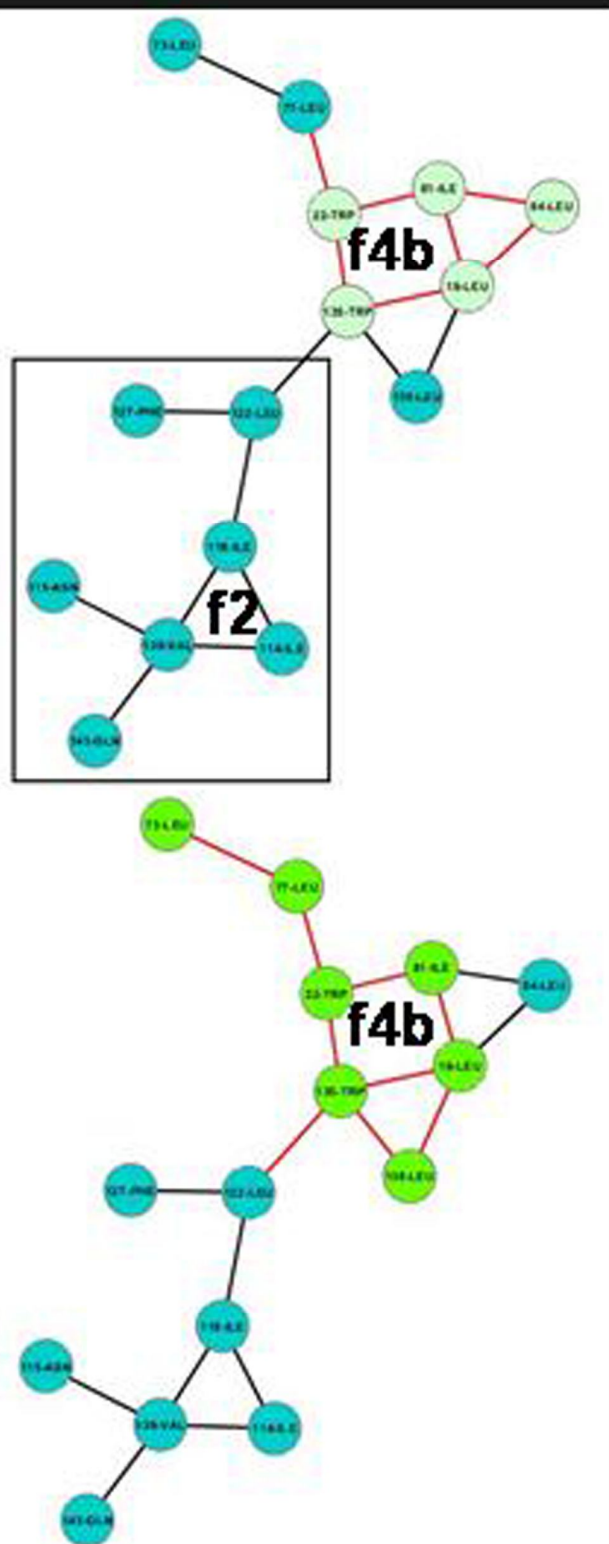

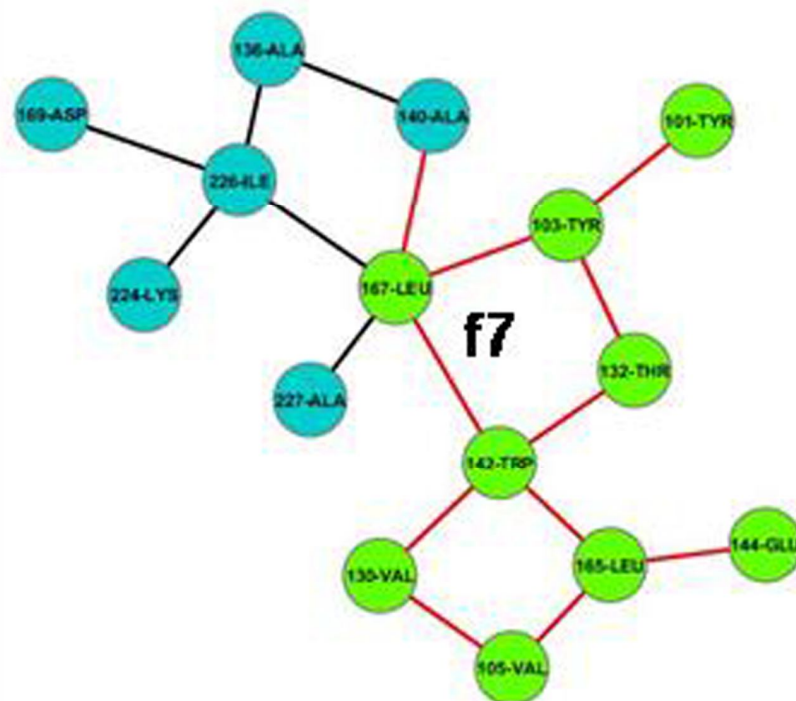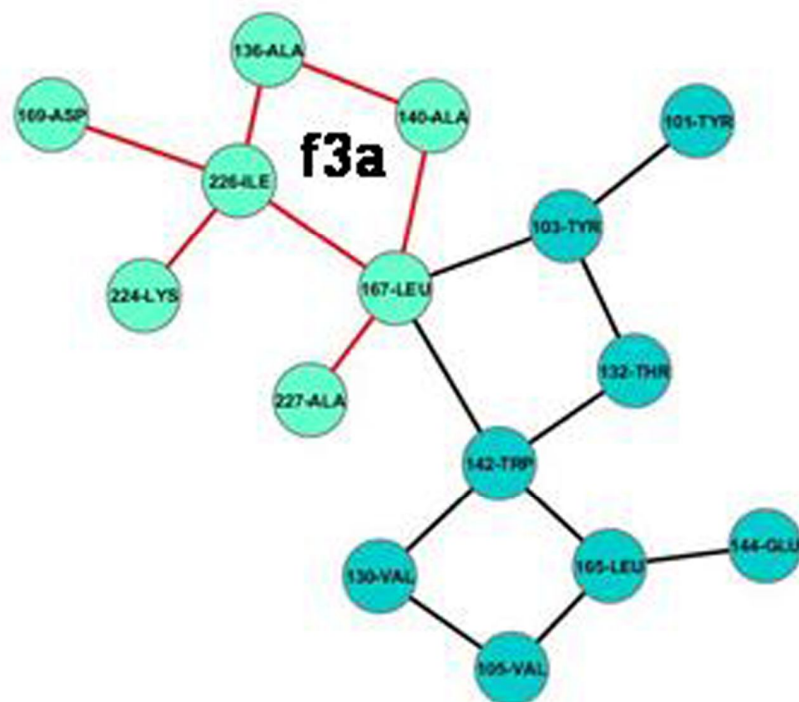

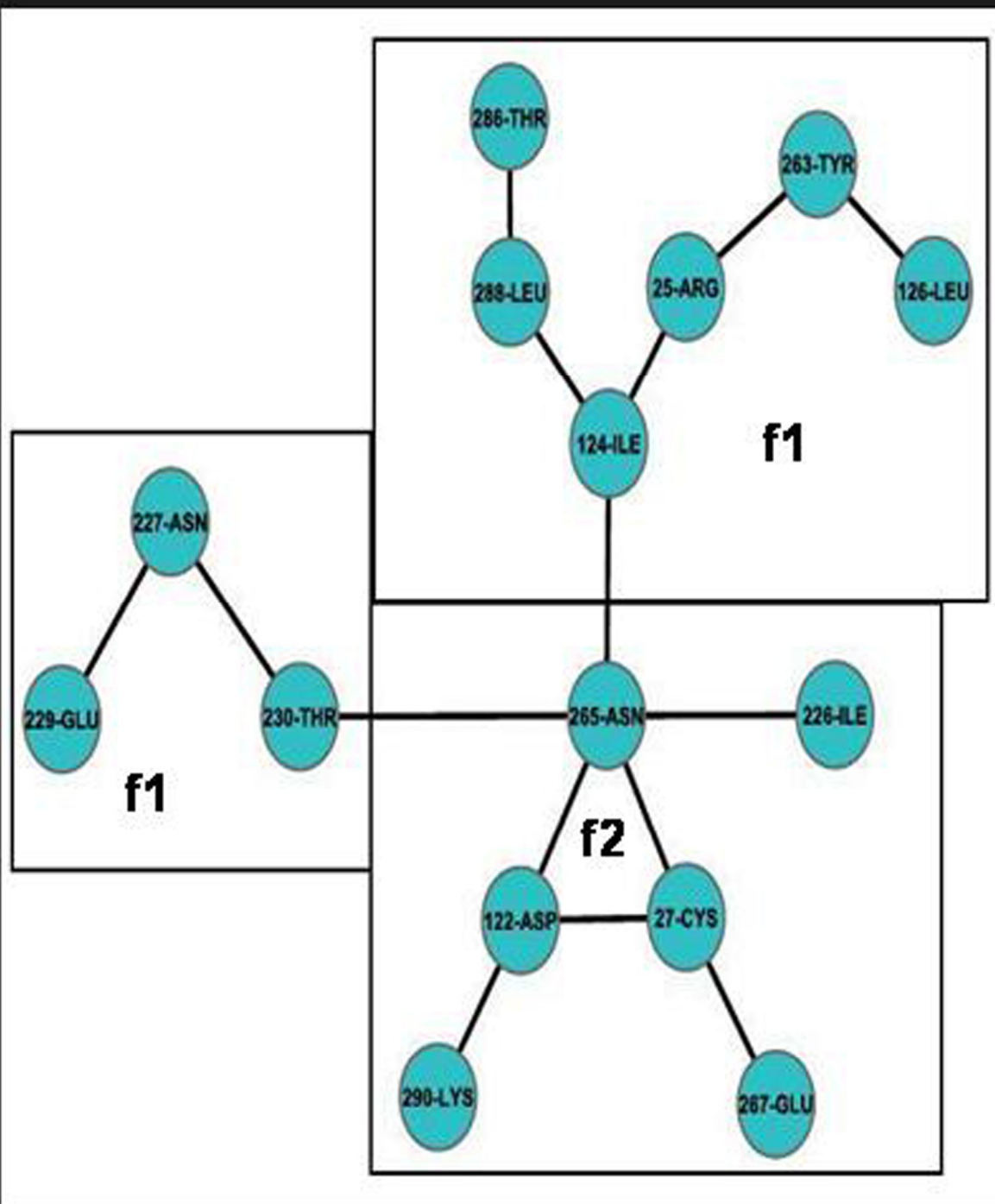

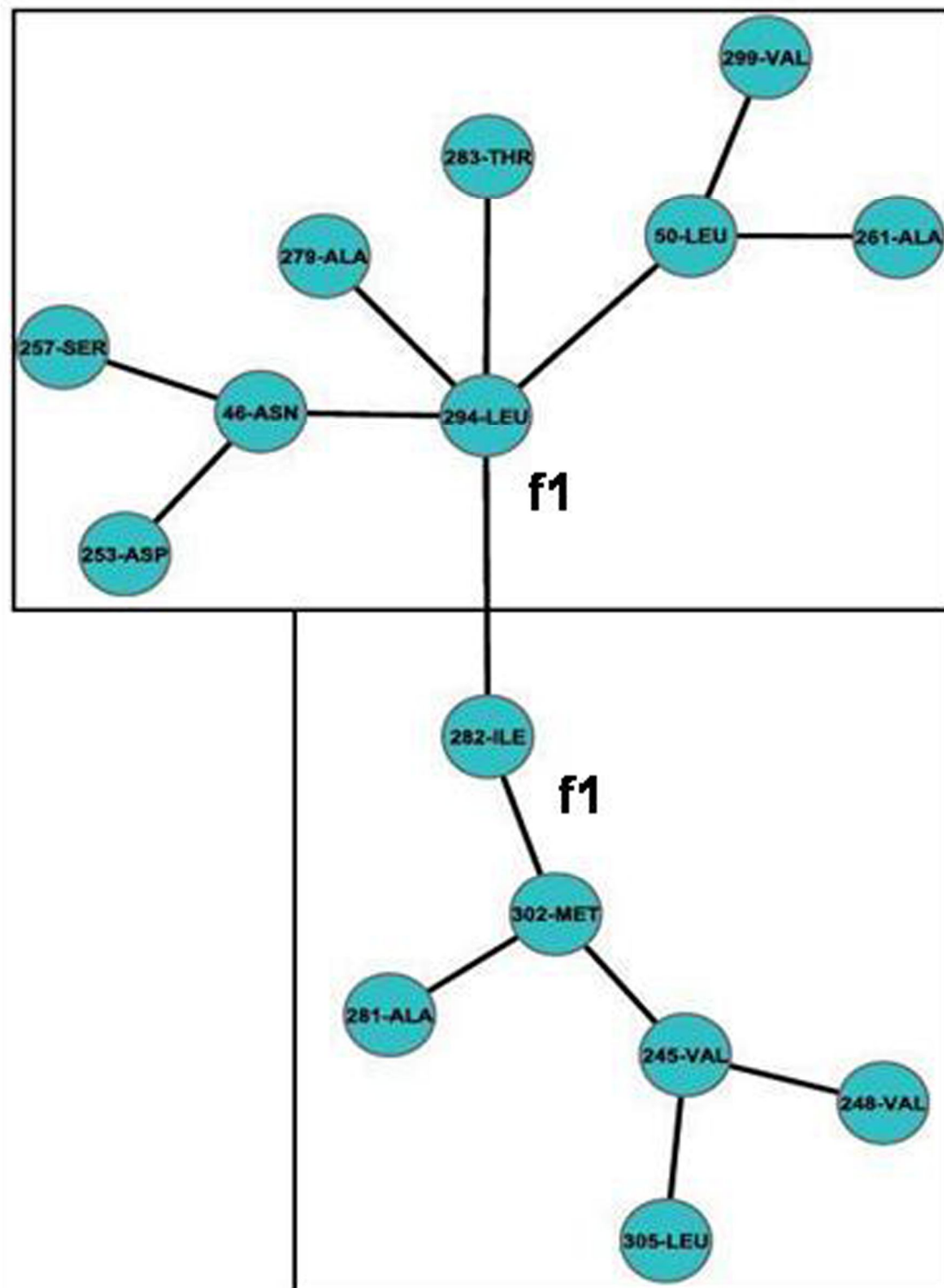

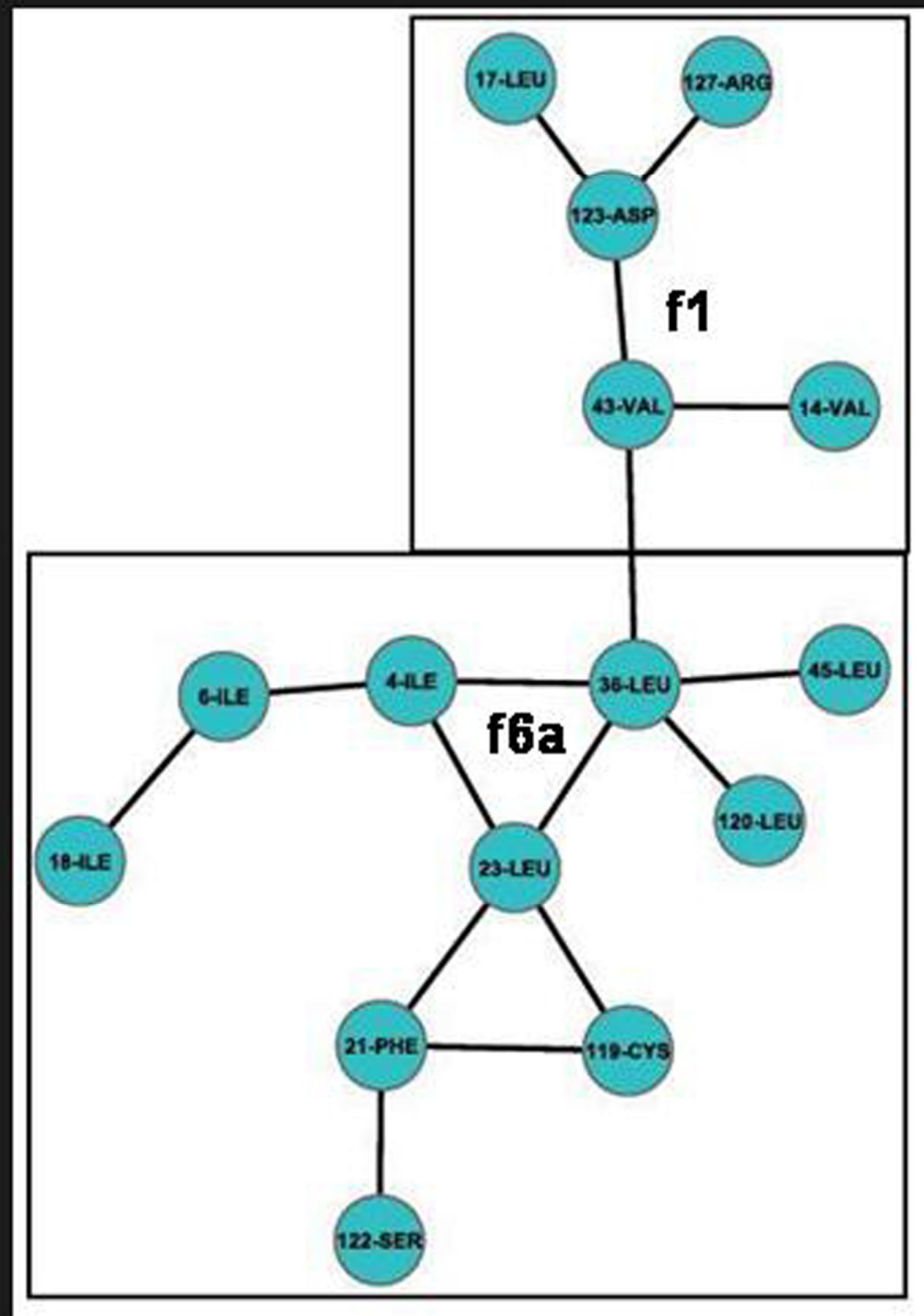

2R6Z\_035

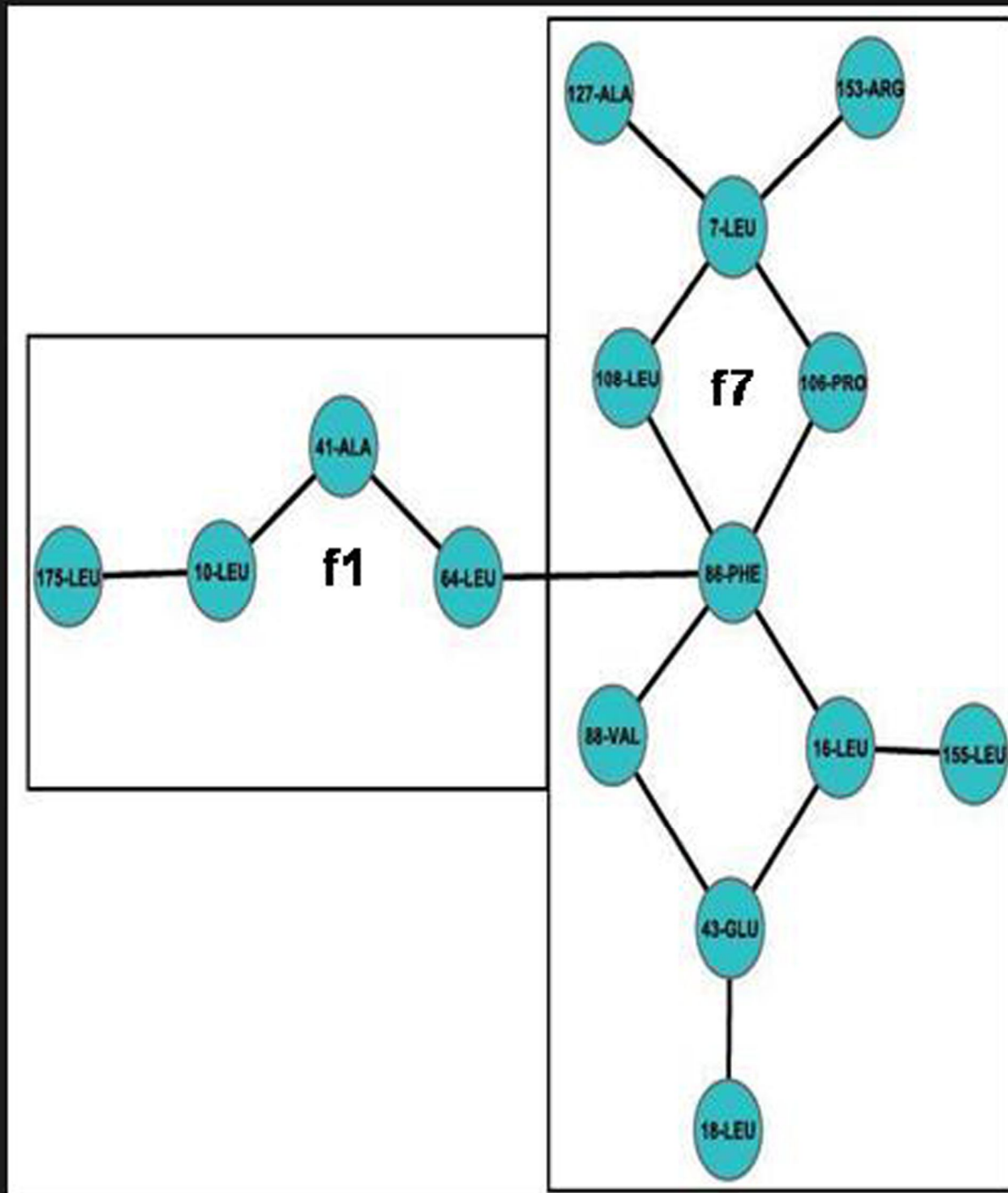

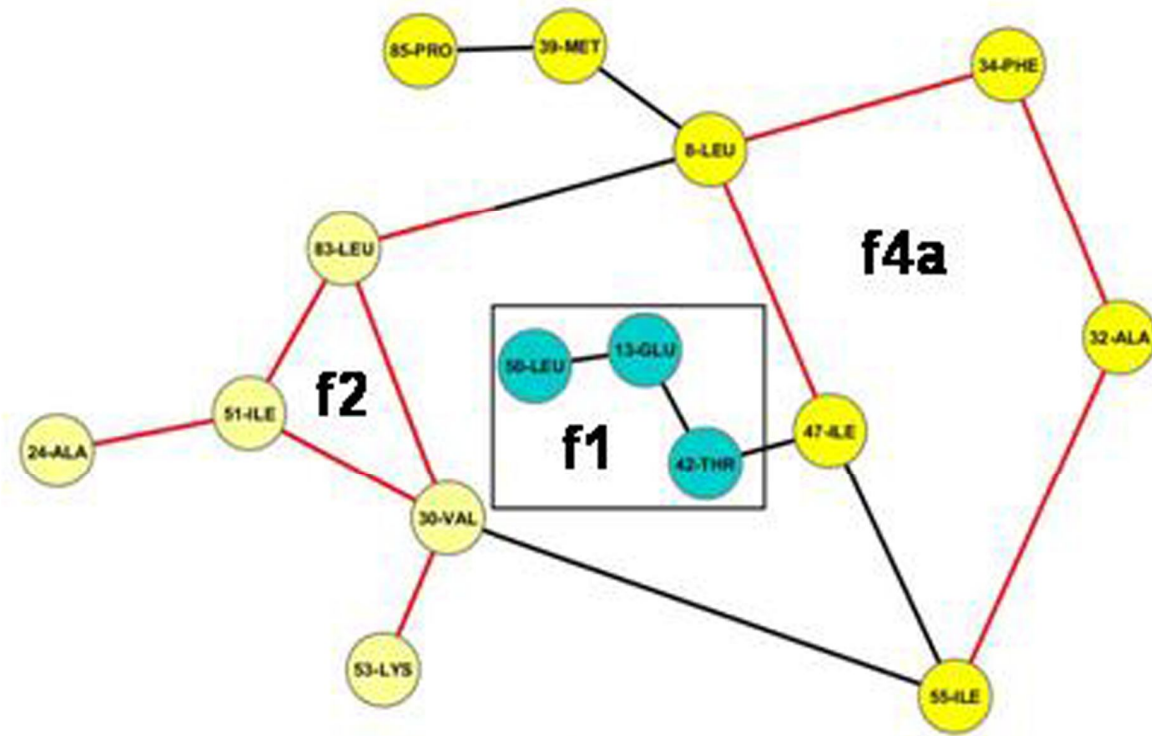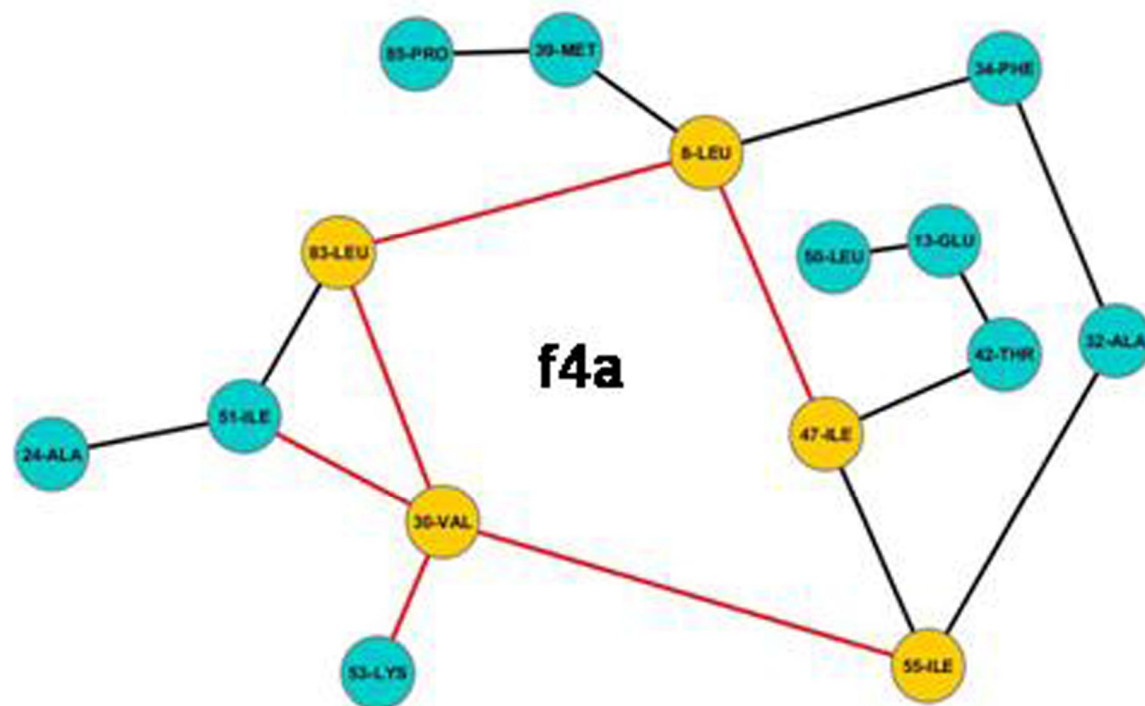

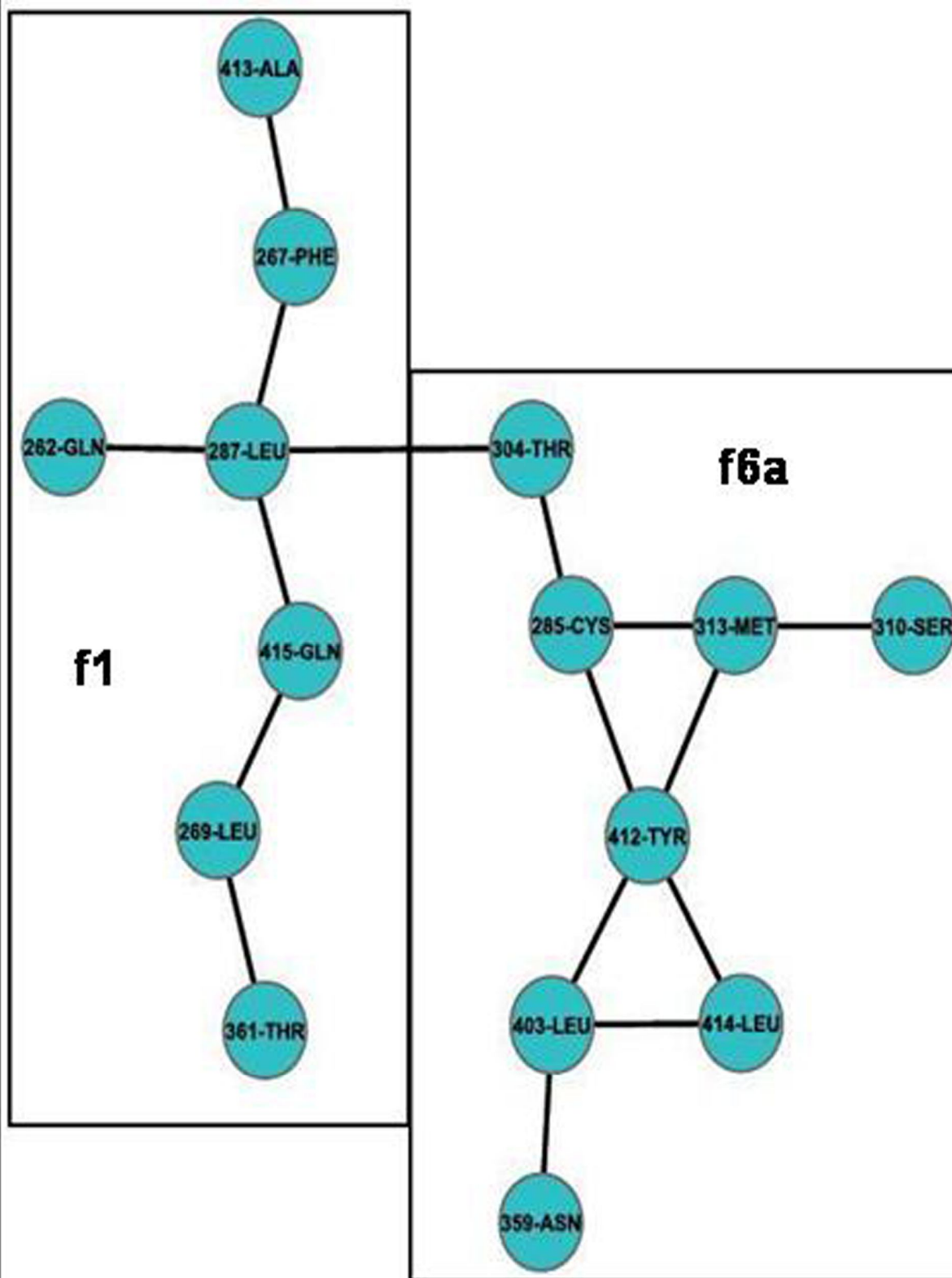

3BIQ\_038

Supplement: Additional file 12 — Dataset S1. Surface contact networks constituted of 15 nodes resolved into optimal set of motifs. Network diagrams of 38 contact networks of size 15 (ASCN) resolved into optimum sets of motifs (or variants) which are either components (separated by boxes) or induced subgraphs (highlighted with different colors). Families of these motifs are also mentioned. Source PDB IDs are displayed at the (right) bottom of each graph. [file 1471-2105-12-195-S12.PDF]

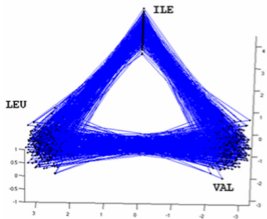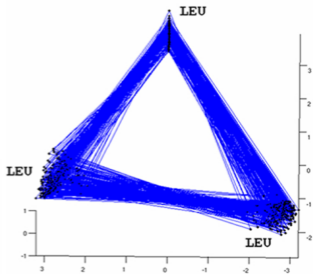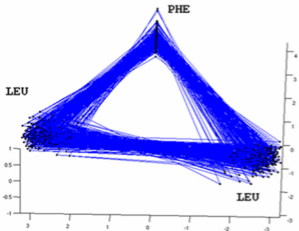

Supplement: Additional file 14 — Figure S7. Optimally superposed triangles. Triangles (formed by joining the origins of the internal frames based on the three residues in a triplet clique) sampled from compositions belonging to categories C1 (all three residues different: top left), C2 (two residues identical: bottom left) and C3 (all three residues identical: right) superposed onto each other. [file 1471-2105-12-195-S14.PDF]
