# Supplementary material for: Mapping the distribution of packing topologies within protein interiors shows predominant preference for specific packing motifs
Source: BMC Bioinformatics. 2011 May 24;12:195. doi: 10.1186/1471-2105-12-195 (PMC3123238; doi:10.1186/1471-2105-12-195)
Supplement: Additional file 2 — Table S1. List of proteins with very large networks. Accession number (PDB ID), protein-class, polypeptide chain length, network size, fold, overall description of the protein and the source organism have been tabulated for each protein. [file 1471-2105-12-195-S2.DOC]

**Table S1:**

| **Pdb id** | **Class** | **Chain length** | **Network size** | **Fold** | **Description** | **Organism** |
| --- | --- | --- | --- | --- | --- | --- |
| 1EU8_A | α|β | 407 | 162 | Periplasmic binding protein-like II | D-maltodextrin-binding protein | *Thermococcus litoralis* |
| 1C7N_C | α|β | 394 | 165 | PLP-dependent transferase like | Cystalysin | *Treponema denticola* |
| 1Z3T_A | all β | 430 | 167 | Concanavalin A-like lectins/glucanases | Cellobiohydrolase I | *Phanerochaete chrysosporium* |
| 1LWD_B | α|β | 413 | 168 | Isocitrate/Isopropylmalate dehydrogenase-like | NADP-dependent isocitrate dehydrogenase Isocitrate | *Sus scrofa* |
| 1HYO_B | all β ( 499-618), α+β ( 619-917) | 419 | 170 | SH3 like barrel, FAH | Fumarylacetoacetate hydrolase, FAH | *Mus musculus* |
| 1F8M_B | α|β | 427 | 173 | TIM beta/alpha-barrel | Isocitrate lyase | *Mycobacterium tuberculosis* |
| 1ZHX_A | α+β | 434 | 178 | Oxysterol-binding protein-like | Oxysterol-binding protein | *Saccharomyces cerevisiae* |
| 1QWO_A | α|β | 435 | 181 | Phosphoglycerate mutase-like | Phytase (myo-inositol-hexakisphosphate-3-phosphohydrolase) | *Aspergillus fumigatus* |
| 1VDK_B | all α | 457 | 185 | L-aspartase-like | Fumarase | *Thermus thermophilus* |
| 2INC_A | all α | 491 | 189 | Ferritin like | Toluene, o-xylene monooxygenase oxygenase subunit TouA [109788]  contains the TouB(TmoB)-binding YHS (sub) domain | *Pseudomonas stutzeri* |
| 1UG6_A | α|β | 426 | 193 | TIM beta/alpha-barrel | Beta-glucosidase A | *Thermus thermophilus* |
| 2HOR_A | α|β | 425 | 201 | PLP-dependent transferase-like | Alliinase | *Allium sativum* |
| 1I9C_A | α|β | 483 | 206 | TIM beta/alpha-barrel | Glutamate mutase, large subunit | Clostridium cochlearium |
| 1OOY_B | α|β | 468 | 215 | NagB/RpiA/CoA transferase-like | Succinate:CoA transferase | *Sus scrofa* |
| 3B6H_B | α+β | 470 | 216 | unassaigned | prostacyclin synthase | *Homo sapiens* |
| 1JAK_A | α+β (8-150), α|β (151-506) | 499 | 220 | TIM-beta/alpha barrel | beta-N-acetylhexosaminidase | *Streptomyces plicatus* |
| 1Y6V_A | α|β | 449 | 223 | Alkaline phosphatase-like | Alkaline phosphatase | *Escherechia coli* |
