# Supplementary material for: Mapping the distribution of packing topologies within protein interiors shows predominant preference for specific packing motifs
Source: BMC Bioinformatics. 2011 May 24;12:195. doi: 10.1186/1471-2105-12-195 (PMC3123238; doi:10.1186/1471-2105-12-195)
Supplement: Additional file 3 — Table S2. Distribution of networks by size amongst protein classes. The top row contains the fraction of the polypeptide chains in each class (bold in parenthesis). Along with frequency, the propensity (see Methods, section: propensity) of the network of a given size to be found in a particular class (enclosed in parenthesis) is also given. [file 1471-2105-12-195-S3.DOC]

**Table S2.**

| **Network size (number of nodes)** | **Number of networks** | | | |
| --- | --- | --- | --- | --- |
| **All α**  **(0.185)** | **All β**  **(0.199)** | **α|β**  **(0.325)** | **α+β**  **(0.291)** |
| 3 | 110 (0.85) | 133 (0.96) | 263 (1.16) | 191 (0.94) |
| 4 | 44 (0.72) | 66 (1.01) | 131 (1.23) | 85 (0.89) |
| 5 | 28 (0.84) | 39 (1.09) | 62 (1.06) | 51 (0.97) |
| 6 | 24 (0.89) | 35 (1.21) | 52 (1.10) | 34 (0.80) |
| 7 | 16 (1.09) | 12 (0.76) | 30 (1.17) | 21 (0.91) |
| 8 | 14 (1.13) | 14 (1.05) | 22 (1.01) | 17 (0.87) |
| 9 | 6 (0.62) | 10 (0.97) | 14 (0.83) | 22 (1.46) |
| 10 | 8 (0.86) | 5 (0.50) | 20 (1.23) | 17 (1.17) |
| 11-20 | 48 (0.66) | 86 (1.10) | 126 (0.99) | 132 (1.16) |
| 21-30 | 34 (0.61) | 70 (1.17) | 108 (1.07) | 98 (1.06) |
| 31-40 | 20 (0.54) | 33 (0.83) | 93 (1.42) | 55 (0.94) |
| 41-50 | 11 (0.59) | 14 (0.70) | 43 (1.32) | 32 (1.10) |
| 51-100 | 30 (0.88) | 21 (0.57) | 98 (1.63) | 35 (0.66) |
| 101-150 | 8 (0.80) | 1 (0.09) | 32 (1.82) | 13 (0.83) |
| 151-200 | 2 (1.35) | 0 (0.0) | 6 (2.30) | 0 (0.0) |
| 201-250 | 0 (0.0) | 0 (0.0) | 4 (2.46) | 1 (0.68) |
