# Supplementary material for: Mapping the distribution of packing topologies within protein interiors shows predominant preference for specific packing motifs
Source: BMC Bioinformatics. 2011 May 24;12:195. doi: 10.1186/1471-2105-12-195 (PMC3123238; doi:10.1186/1471-2105-12-195)
Supplement: Additional file 4 — Table S3. Correlation between number of (unique) motifs: observed in the database versus simulated from random graphs. For a given network size (n), the number of unique motifs observed in the database is tabulated along with the corresponding number generated from simulated random graphs without and with cutoffs on the highest attainable degree of a node. [file 1471-2105-12-195-S4.DOC]

**Table S3.**

| **Network Size (n)** | **Highest possible degree of a node**  **(n-1)** | **Observed Highest Degree** | | **Number of Motifs** | | **Cutoff on the highest attainable degree** | **Number of unique random**  **graphs** |
| --- | --- | --- | --- | --- | --- | --- | --- |
|  |  | **ASCN** | **APCN** | **ASCN** | **APCN** |  |  |
| 3 | 2 | 2 | 2 | 2 | 2 | 2 | 2 |
| 4 | 3 | 3 | 3 | 5 | 5 | 3 | 6 |
| 5 | 4 | 4 | 4 | 12 | 13 | 4 | 22 |
| 6 | 5 | 5 | 5 | 25 | 36 | 5 | 114 |
| 7 | 6 | 5 | 6 | 45 | 61 | 4 | 315 |
|  |  |  |  |  |  | 5 | 639 |
|  |  |  |  |  |  | 6 | 782 |
| 8 | 7 | 6 | 5 | 55 | 76 | 4 | 1179 |
|  |  |  |  |  |  | 5 | 4300 |
|  |  |  |  |  |  | 6 | 7151 |
| 9 | 8 | 5 | 7 | 46 | 93 | 4 | 1410 |
|  |  |  |  |  |  | 5 | 10000 |
|  |  |  |  |  |  | 6 | 25864 |
|  |  |  |  |  |  | 7 | 35002 |
| 10 | 9 | 6 | 7 | 51 | 91 | 4 | 400 |
|  |  |  |  |  |  | 5 | 4512 |
|  |  |  |  |  |  | 6 | 20701 |
|  |  |  |  |  |  | 7 | 39654 |
