# Supplementary material for: Mapping the distribution of packing topologies within protein interiors shows predominant preference for specific packing motifs
Source: BMC Bioinformatics. 2011 May 24;12:195. doi: 10.1186/1471-2105-12-195 (PMC3123238; doi:10.1186/1471-2105-12-195)
Supplement: Additional file 10 — Table S4. Distribution of motifs and families in each protein-class. Motifs (obtained from ASCN, Sm > = 0.4, Ov > = 0.08) are sorted according to size (up to 7 nodes) and grouped under their respective families. The frequency of their occurrence is given along with the propensity (see Methods, section: propensity) of a given class to contain a family of motifs. Other than f1 and f2, rest of the families do not have sufficient members for robust statistics. [file 1471-2105-12-195-S10.DOC]

**Table S4:**

| **Family** | **Motif identifier** | **Motif size** | **class** | | | |
| --- | --- | --- | --- | --- | --- | --- |
|  | | | **All α** | **All β** | **α|β** | **α+β** |
| f1 | 211-12-12 | 3 | 105 | 127 | 243 | 176 |
|  | 221-221-12-12 | 4 | 25 | 36 | 76 | 55 |
|  | 3111-13-13-13 | 4 | 11 | 21 | 33 | 11 |
|  | 222-221-221-12-12 | 5 | 11 | 7 | 24 | 11 |
|  | 3211-231-13-13-12 | 5 | 10 | 18 | 21 | 24 |
|  | 41111-14-14-14-14 | 5 | 3 | 1 | 1 | 2 |
|  | 222-222-221-221-12-12 | 6 | 4 | 4 | 6 | 1 |
|  | 3311-3311-13-13-13-13 | 6 | 2 | 1 | 3 | 4 |
|  | 3221-231-231-13-12-12 | 6 | 5 | 6 | 7 | 7 |
|  | 3211-232-221-13-13-12 | 6 | 4 | 7 | 7 | 4 |
|  | 42111-241-14-14-14-12 | 6 | 2 | 0 | 5 | 3 |
|  | 511111-15-15-15-15-15 | 6 | 1 | 0 | 0 | 0 |
|  | 222-222-222-221-221-12-12 | 7 | 3 | 0 | 1 | 1 |
|  | 3221-232-231-221-13-12-12 | 7 | 3 | 1 | 6 | 3 |
|  | 3211-232-222-221-13-13-12 | 7 | 0 | 0 | 1 | 0 |
|  | 42111-242-221-14-14-14-12 | 7 | 0 | 1 | 0 | 2 |
|  | 42211-241-241-14-14-12-12 | 7 | 0 | 0 | 0 | 1 |
|  | 3321-3311-231-13-13-13-12 | 7 | 2 | 1 | 0 | 3 |
|  | 3222-231-231-231-12-12-12 | 7 | 1 | 0 | 1 | 0 |
|  | 43111-3411-14-14-14-13-13 | 7 | 0 | 1 | 1 | 0 |
|  | 521111-251-15-15-15-15-12 | 7 | 0 | 0 | 0 | 1 |
|  |  | 3-7 | 192 | 232 | 436 | 309 |
|  | f1(given class) / f1 (all classes): |  | 0.164 | 0.198 | 0.372 | 0.264 |
|  | Fraction of each chains in the database: |  | 0.185 | 0.199 | 0.325 | 0.291 |
|  | **Propensity of f1 in each class:** |  | **0.88** | **0.99** | **1.14** | **0.91** |
|  |  |  |  |  |  |  |
| f2 | 222-222-222 | 3 | 5 | 6 | 20 | 15 |
|  | 3221-232-232-13 | 4 | 7 | 8 | 19 | 17 |
|  | 3222-232-232-231-12 | 5 | 2 | 4 | 2 | 2 |
|  | 42211-242-242-14-14 | 5 | 0 | 0 | 3 | 0 |
|  | 3321-3321-233-13-13 | 5 | 2 | 8 | 5 | 5 |
|  | 3322-3321-233-231-13-12 | 6 | 0 | 3 | 6 | 2 |
|  | 43211-3421-243-14-14-13 | 6 | 0 | 0 | 1 | 2 |
|  | 3322-3311-232-232-13-13 | 6 | 1 | 1 | 3 | 0 |
|  | 42221-242-242-241-14-12 | 6 | 0 | 1 | 2 | 1 |
|  | 3222-232-232-232-221-12 | 6 | 1 | 1 | 3 | 2 |
|  | 3331-3331-3331-13-13-13 | 6 | 0 | 1 | 1 | 1 |
|  | 522111-252-252-15-15-15 | 6 | 0 | 0 | 0 | 1 |
|  | 3332-3321-3311-233-13-13-13 | 7 | 1 | 0 | 2 | 0 |
|  | 3322-3321-233-232-221-13-12 | 7 | 0 | 0 | 0 | 1 |
|  | 3332-3331-3331-231-13-13-12 | 7 | 1 | 0 | 0 | 0 |
|  | 3322-3322-233-231-231-12-12 | 7 | 1 | 0 | 0 | 1 |
|  | 43211-3422-243-231-14-14-12 | 7 | 1 | 1 | 1 | 0 |
|  | 42221-242-242-242-221-14-12 | 7 | 0 | 0 | 1 | 1 |
|  | 43311-3431-3431-14-14-13-13 | 7 | 0 | 0 | 0 | 1 |
|  |  | 3-7 | 22 | 34 | 69 | 52 |
|  | f2(given class) / f2 (all classes): |  | 0.124 | 0.192 | 0.389 | 0.293 |
|  | Fraction of each chains in the database: |  | 0.185 | 0.199 | 0.325 | 0.291 |
|  | **Propensity of f2 in each class:** |  | **0.67** | **0.96** | **1.20** | **1.01** |
|  |  |  |  |  |  |  |
| f3a | 222-222-222-222 | 4 | 1 | 0 | 2 | 2 |
|  | 3221-232-232-222-13 | 5 | 1 | 1 | 1 | 3 |
|  | 3222-232-232-231-222-12 | 6 | 1 | 2 | 1 | 1 |
|  | 3321-3321-232-232-13-13 | 6 | 1 | 2 | 0 | 1 |
|  | 3221-3221-233-233-13-13 | 6 | 1 | 0 | 0 | 1 |
|  | 42211-242-242-222-14-14 | 6 | 0 | 1 | 0 | 0 |
|  | 43211-3421-242-232-14-14-13 | 7 | 1 | 0 | 1 | 0 |
|  | 3322-3321-232-232-231-13-12 | 7 | 0 | 2 | 1 | 0 |
|  | 3322-3311-232-232-222-13-13 | 7 | 0 | 0 | 0 | 1 |
|  | 3331-3321-3321-233-13-13-13 | 7 | 0 | 0 | 2 | 0 |
|  | 42211-3221-243-243-14-14-13 | 7 | 0 | 0 | 1 | 1 |
|  | 42221-242-242-241-222-14-12 | 7 | 0 | 0 | 1 | 0 |
|  |  | 4-7 | 6 | 8 | 10 | 10 |
|  | f3a (given class) / f3a (all classes): |  | 0.176 | 0.235 | 0.294 | 0.294 |
|  | Fraction of each chains in the database: |  | 0.185 | 0.199 | 0.325 | 0.291 |
|  | **Propensity of f3a in each class:** |  | **0.95** | **1.18** | **0.90** | **1.01** |
|  |  |  |  |  |  |  |
| f3b | 3322-3322-233-233 | 4 | 0 | 1 | 1 | 0 |
|  | 43221-3422-243-243-14 | 5 | 0 | 1 | 1 | 1 |
|  | 3332-3332-3331-233-13 | 5 | 0 | 1 | 0 | 0 |
|  | 43222-3422-243-243-241-12 | 6 | 0 | 1 | 1 | 0 |
|  | 43321-3432-3431-243-14-13 | 6 | 0 | 0 | 2 | 0 |
|  | 43321-3432-3432-243-231-14-12 | 7 | 0 | 0 | 0 | 1 |
|  | 3332-3332-3332-233-232-221-12 | 7 | 0 | 0 | 1 | 0 |
|  | 43222-3422-243-243-242-221-12 | 7 | 1 | 0 | 0 | 0 |
|  | 43321-3432-3432-241-233-14-12 | 7 | 0 | 0 | 1 | 0 |
|  | 43311-3433-3433-3331-14-14-13 | 7 | 0 | 1 | 0 | 0 |
|  | 532221-3522-253-253-251-15-12 | 7 | 0 | 0 | 0 | 1 |
|  | 43322-3422-3411-243-243-13-13 | 7 | 0 | 1 | 0 | 0 |
|  |  | 4-7 | 1 | 6 | 7 | 3 |
|  | f3b (given class) / f3b (all classes): |  | 0.058 | 0.352 | 0.411 | 0.176 |
|  | Fraction of each chains in the database: |  | 0.185 | 0.199 | 0.325 | 0.291 |
|  | **Propensity of f3b in each class:** |  | **0.31** | **1.77** | **1.26** | **0.61** |
|  |  |  |  |  |  |  |
| f4a | 222-222-222-222-222 | 5 | 0 | 0 | 1 | 0 |
|  | 3221-232-232-222-222-13 | 6 | 0 | 0 | 1 | 2 |
|  |  | 5-7 | 0 | 0 | 2 | 2 |
|  | f4a (given class) / f4a (all classes): |  | 0.0 | 0.0 | 0.5 | 0.5 |
|  | Fraction of each chains in the database: |  | 0.185 | 0.199 | 0.325 | 0.291 |
|  | **Propensity of f4a in each class:** |  | **0.0** | **0.0** | **1.54** | **1.72** |
|  |  |  |  |  |  |  |
| f4b | 3322-3322-233-232-232 | 5 | 0 | 0 | 2 | 2 |
|  | 43322-3432-3432-242-242-233 | 6 | 1 | 0 | 1 | 0 |
|  | 43221-3422-243-242-232-14 | 6 | 0 | 1 | 0 | 0 |
|  | 43322-3432-3432-243-242-232 | 6 | 0 | 1 | 0 | 0 |
|  | 43211-3432-3322-243-233-14-14 | 7 | 0 | 0 | 1 | 0 |
|  | 43222-3422-243-242-241-232-12 | 7 | 0 | 0 | 1 | 0 |
|  |  | 5-7 | 1 | 2 | 5 | 2 |
|  | f4b (given class) / f4b (all classes): |  | 0.1 | 0.2 | 0.5 | 0.2 |
|  | Fraction of each chains in the database: |  | 0.185 | 0.199 | 0.325 | 0.291 |
|  | **Propensity of f4b in each class:** |  | **0.54** | **1.00** | **1.54** | **0.67** |
|  |  |  |  |  |  |  |
| f5 | 44422-44421-44421-244-244-14-14 | 7 | 1 | 0 | 0 | 0 |
|  | 43332-3432-3432-3421-243-233-13 | 7 | 0 | 0 | 1 | 0 |
|  |  | 5-7 | 1 | 0 | 1 | 0 |
|  | f5 (given class) / f5 (all classes): |  | 0.5 | 0.0 | 0.5 | 0.0 |
|  | Fraction of each chains in the database: |  | 0.185 | 0.199 | 0.325 | 0.291 |
|  | **Propensity of f5 in each class:** |  | **2.70** | **0.0** | **1.54** | **0.0** |
|  |  |  |  |  |  |  |
|  |  |  |  |  |  |  |
| f6a | 43222-3421-243-242-242-13 | 6 | 0 | 1 | 2 | 0 |
|  | 43322-3421-3421-243-243-13-13 | 7 | 0 | 0 | 2 | 2 |
|  | 532221-3521-253-252-252-15-13 | 7 | 0 | 0 | 1 | 0 |
|  |  | 5-7 | 0 | 1 | 5 | 2 |
|  | f6a (given class) / f6a (all classes): |  | 0.0 | 0.125 | 0.625 | 0.25 |
|  | Fraction of each chains in the database: |  | 0.185 | 0.199 | 0.325 | 0.291 |
|  | **Propensity of f6a in each class:** |  | **0.0** | **0.63** | **1.92** | **0.86** |
|  |  |  |  |  |  |  |
|  |  |  |  |  |  |  |
| f6b | 3322-3322-232-232-232-232 | 6 | 0 | 1 | 0 | 0 |
|  |  | 6-7 | 0 | 1 | 0 | 0 |
|  | f6b (given class) / f6b (all classes): |  | 0.0 | 1.0 | 0.0 | 0.0 |
|  | Fraction of each chains in the database: |  | 0.185 | 0.199 | 0.325 | 0.291 |
|  | **Propensity of f6b in each class:** |  | **0.0** | **5.03** | **0.0** | **0.0** |
|  |  |  |  |  |  |  |
| f7 | 42222-242-242-242-242-222-222 | 7 | 0 | 1 | 0 | 0 |
|  |  | 7 | 0 | 1 | 0 | 0 |
|  | f7 (given class) / f7 (all classes): |  | 0.0 | 1.0 | 0.0 | 0.0 |
|  | Fraction of each chains in the database: |  | 0.185 | 0.199 | 0.325 | 0.291 |
|  | **Propensity of f7 in each class:** |  | **0.0** | **5.02** | **0.0** | **0.0** |
|  |  |  |  |  |  |  |
| f8a | 43332-3433-3433-3433-241-12 | 7 | 0 | 1 | 0 | 0 |
|  |  | 6-7 | 0 | 1 | 0 | 0 |
|  | f8a (given class) / f8a (all classes): |  | 0.0 | 1.0 | 0.0 | 0.0 |
|  | Fraction of each chains in the database: |  | 0.185 | 0.199 | 0.325 | 0.291 |
|  | **Propensity of f8a in each class:** |  | **0.0** | **5.02** | **0.0** | **0.0** |
|  |  |  |  |  |  |  |
| f8b | 44432-44332-44322-3443-3443-244-244 | 7 | 0 | 0 | 0 | 1 |
|  | 43221-3431-3322-243-243-14-13 | 7 | 0 | 0 | 0 | 1 |
|  |  | 7 | 0 | 0 | 0 | 2 |
|  | f8b (given class) / f8b (all classes): |  | 0.0 | 0.0 | 0.0 | 1.0 |
|  | Fraction of each chains in the database: |  | 0.185 | 0.199 | 0.325 | 0.291 |
|  | **Propensity of f8b in each class:** |  | **0.0** | **0.0** | **0.0** | **3.44** |
|  |  |  |  |  |  |  |
| f8c | 44332-44332-3441-3441-244-13-13 | 7 | 0 | 0 | 1 | 0 |
|  |  | 6-7 | 0 | 0 | 1 | 0 |
|  | f8c (given class) / f8c (all classes): |  | 0.0 | 0.0 | 1.0 | 0.0 |
|  | Fraction of each chains in the database: |  | 0.185 | 0.199 | 0.325 | 0.291 |
|  | **Propensity of f8c in each class:** |  | **0.0** | **0.0** | **3.07** | **0.0** |
|  |  |  |  |  |  |  |
