# Supplementary material for: Mapping the distribution of packing topologies within protein interiors shows predominant preference for specific packing motifs
Source: BMC Bioinformatics. 2011 May 24;12:195. doi: 10.1186/1471-2105-12-195 (PMC3123238; doi:10.1186/1471-2105-12-195)
Supplement: Additional file 11 — Table S5. Distribution of motifs and families obtained at different contact cutoffs. Motifs are sorted according to size (up to 7 nodes) and grouped under their respective families. Cutoffs on Sm and Ov are mentioned in parenthesis. Results for the chosen set of cutoff values, used in the analysis (0.4, 0.08) are highlighted in bold. [file 1471-2105-12-195-S11.DOC]

**Table S5:**

| **Family** | **Motif identifier** | **Motif size** | **Cutoff (Sm, Ov)** | | | | |
| --- | --- | --- | --- | --- | --- | --- | --- |
|  |  |  | **(0.0, 0.0)** | **(0.2, 0.05)** | **(0.3, 0.07)** | **(0.4, 0.08)** | **(0.5, 0.1)** |
| f1 | 211-12-12 | 3 | 40 | 135 | 304 | **660** | 1938 |
|  | 221-221-12-12 | 4 | 16 | 45 | 101 | **205** | 697 |
|  | 3111-13-13-13 | 4 | 5 | 15 | 30 | **85** | 246 |
|  | 222-221-221-12-12 | 5 | 2 | 12 | 32 | **53** | 270 |
|  | 3211-231-13-13-12 | 5 | 2 | 11 | 31 | **75** | 287 |
|  | 41111-14-14-14-14 | 5 | 0 | 1 | 4 | **7** | 17 |
|  | 222-222-221-221-12-12 | 6 | 2 | 5 | 9 | **15** | 101 |
|  | 3311-3311-13-13-13-13 | 6 | 0 | 1 | 2 | **10** | 28 |
|  | 3221-231-231-13-12-12 | 6 | 0 | 2 | 8 | **26** | 110 |
|  | 3211-232-221-13-13-12 | 6 | 0 | 0 | 11 | **22** | 116 |
|  | 42111-241-14-14-14-12 | 6 | 0 | 1 | 5 | **10** | 28 |
|  | 511111-15-15-15-15-15 | 6 | 0 | 0 | 0 | **0** | 1 |
|  | 222-222-222-221-221-12-12 | 7 | 1 | 0 | 2 | **5** | 42 |
|  | 3221-232-231-221-13-12-12 | 7 | 0 | 1 | 5 | **14** | 68 |
|  | 3211-232-222-221-13-13-12 | 7 | 1 | 1 | 3 | **1** | 34 |
|  | 42111-242-221-14-14-14-12 | 7 | 0 | 0 | 0 | **3** | 8 |
|  | 42211-241-241-14-14-12-12 | 7 | 0 | 0 | 0 | **1** | 14 |
|  | 3321-3311-231-13-13-13-12 | 7 | 0 | 1 | 2 | **7** | 38 |
|  | 3222-231-231-231-12-12-12 | 7 | 0 | 0 | 1 | **2** | 11 |
|  | 43111-3411-14-14-14-13-13 | 7 | 0 | 0 | 1 | **2** | 9 |
|  | 521111-251-15-15-15-15-12 | 7 | 0 | 0 | 0 | **0** | 3 |
|  | 3211-3211-233-13-13-13-13 | 7 | 0 | 0 | 0 | **0** | 12 |
|  | 222-222-222-221-221-12-12 | 7 | 0 | 0 | 0 | **5** | 0 |
|  |  | 3-7 | 69 | 231 | 551 | **1204** | 4078 |
|  |  |  |  |  |  |  |  |
| f2 | 222-222-222 | 3 | 18 | 27 | 32 | **47** | 57 |
|  | 3221-232-232-13 | 4 | 17 | 33 | 26 | **52** | 60 |
|  | 3222-232-232-231-12 | 5 | 6 | 7 | 6 | **11** | 17 |
|  | 42211-242-242-14-14 | 5 | 0 | 2 | 3 | **3** | 8 |
|  | 3321-3321-233-13-13 | 5 | 7 | 7 | 12 | **21** | 24 |
|  | 3322-3321-233-231-13-12 | 6 | 4 | 3 | 5 | **12** | 23 |
|  | 43211-3421-243-14-14-13 | 6 | 1 | 2 | 1 | **3** | 3 |
|  | 3322-3311-232-232-13-13 | 6 | 1 | 1 | 1 | **5** | 5 |
|  | 42221-242-242-241-14-12 | 6 | 0 | 1 | 1 | **4** | 5 |
|  | 3222-232-232-232-221-12 | 6 | 1 | 0 | 6 | **8** | 5 |
|  | 3331-3331-3331-13-13-13 | 6 | 1 | 0 | 0 | **3** | 7 |
|  | 522111-252-252-15-15-15 | 6 | 0 | 1 | 1 | **1** | 0 |
|  | 3332-3321-3311-233-13-13-13 | 7 | 0 | 0 | 2 | **3** | 6 |
|  | 3322-3321-233-232-221-13-12 | 7 | 0 | 2 | 1 | **1** | 4 |
|  | 3332-3331-3331-231-13-13-12 | 7 | 0 | 0 | 0 | **1** | 6 |
|  | 3322-3322-233-231-231-12-12 | 7 | 0 | 0 | 2 | **2** | 4 |
|  | 43211-3422-243-231-14-14-12 | 7 | 0 | 0 | 1 | **3** | 4 |
|  | 42221-242-242-242-221-14-12 | 7 | 0 | 1 | 1 | **2** | 2 |
|  | 42222-242-242-241-241-12-12 | 7 | 0 | 0 | 0 | **0** | 1 |
|  | 43221-3421-243-241-14-13-12 | 7 | 0 | 0 | 1 | **3** | 4 |
|  | 43221-3411-242-242-14-13-13 | 7 | 0 | 0 | 2 | **0** | 3 |
|  | 3222-3211-233-232-232-13-13 | 7 | 0 | 1 | 1 | **1** | 3 |
|  | 43311-3431-3431-14-14-13-13 | 7 | 0 | 0 | 0 | **1** | 0 |
|  |  | 3-7 | 56 | 88 | 105 | **187** | 251 |
|  |  |  |  |  |  |  |  |
| f3a | 222-222-222-222 | 4 | 0 | 2 | 3 | **5** | 9 |
|  | 3221-232-232-222-13 | 5 | 0 | 5 | 3 | **7** | 9 |
|  | 3222-232-232-231-222-12 | 6 | 0 | 2 | 1 | **5** | 5 |
|  | 3321-3321-232-232-13-13 | 6 | 0 | 0 | 0 | **4** | 5 |
|  | 3221-3221-233-233-13-13 | 6 | 0 | 1 | 0 | **2** | 3 |
|  | 42211-242-242-222-14-14 | 6 | 0 | 0 | 0 | **1** | 1 |
|  | 43211-3421-242-232-14-14-13 | 7 | 1 | 0 | 1 | **2** | 0 |
|  | 3322-3321-232-232-231-13-12 | 7 | 0 | 2 | 4 | **3** | 8 |
|  | 3322-3311-232-232-222-13-13 | 7 | 0 | 0 | 0 | **1** | 2 |
|  | 3331-3321-3321-233-13-13-13 | 7 | 0 | 0 | 0 | **2** | 2 |
|  | 42211-3221-243-243-14-14-13 | 7 | 0 | 1 | 0 | **2** | 1 |
|  | 3222-232-232-232-222-221-12 | 7 | 0 | 2 | 0 | **0** | 4 |
|  | 43211-3421-242-232-14-14-13 | 7 | 0 | 0 | 0 | **0** | 2 |
|  | 42221-242-242-241-222-14-12 | 7 | 0 | 0 | 0 | **1** | 0 |
|  |  | 4-7 | 1 | 15 | 12 | **35** | 51 |
|  |  |  |  |  |  |  |  |
| f3b | 3322-3322-233-233 | 4 | 13 | 4 | 5 | **2** | 4 |
|  | 43221-3422-243-243-14 | 5 | 1 | 2 | 3 | **3** | 5 |
|  | 3332-3332-3331-233-13 | 5 | 4 | 4 | 1 | **1** | 2 |
|  | 43222-3422-243-243-241-12 | 6 | 3 | 0 | 0 | **2** | 0 |
|  | 43321-3432-3431-243-14-13 | 6 | 1 | 2 | 2 | **2** | 2 |
|  | 44221-44221-244-244-14-14 | 6 | 1 | 1 | 1 | **0** | 0 |
|  | 3332-3332-3332-233-231-12 | 6 | 2 | 1 | 0 | **0** | 0 |
|  | 3333-3333-3331-3331-13-13 | 6 | 2 | 0 | 0 | **0** | 0 |
|  | 43321-3432-3432-243-231-14-12 | 7 | 0 | 0 | 0 | **1** | 1 |
|  | 3333-3333-3332-3331-231-13-12 | 7 | 1 | 0 | 0 | **0** | 0 |
|  | 43331-3433-3431-3431-14-13-13 | 7 | 0 | 0 | 0 | **1** | 1 |
|  | 43321-3432-3432-241-233-14-12 | 7 | 0 | 0 | 0 | **1** | 1 |
|  | 43222-3422-243-243-242-221-12 | 7 | 0 | 0 | 0 | **1** | 0 |
|  | 532221-3522-253-253-251-15-12 | 7 | 0 | 0 | 1 | **1** | 0 |
|  | 43322-3422-3411-243-243-13-13 | 7 | 1 | 0 | 0 | **1** | 0 |
|  |  | 4-7 | 29 | 14 | 13 | **16** | 16 |
|  |  |  |  |  |  |  |  |
| f4a | 222-222-222-222-222 | 5 | 4 | 0 | 0 | **1** | 2 |
|  | 3221-232-232-222-222-13 | 6 | 3 | 0 | 0 | **3** | 2 |
|  | 3332-3322-3321-233-232-232-13 | 7 | 1 | 0 | 0 | **0** | 1 |
|  | 3222-232-232-231-222-222-12 | 7 | 1 | 0 | 0 | **0** | 1 |
|  | 42211-242-242-222-222-14-14 | 7 | 2 | 0 | 1 | **0** | 0 |
|  | 3221-3221-233-232-232-13-13 | 7 | 2 | 0 | 0 | **0** | 2 |
|  | 3321-3321-232-232-222-13-13 | 7 | 0 | 0 | 1 | **0** | 1 |
|  |  | 5-7 | 13 | 0 | 2 | **4** | 9 |
|  |  |  |  |  |  |  |  |
| f4b | 3322-3322-233-232-232 | 5 | 0 | 2 | 2 | **4** | 0 |
|  | 43221-3422-243-242-232-14 | 6 | 0 | 0 | 1 | **1** | 0 |
|  | 3332-3332-3331-232-232-13 | 6 | 0 | 0 | 1 | **0** | 0 |
|  | 43322-3432-3432-242-242-233 | 6 | 0 | 0 | 0 | **1** | 0 |
|  | 43322-3432-3432-243-242-232 | 6 | 1 | 0 | 0 | **1** | 0 |
|  | 43222-3422-243-242-241-232-12 | 7 | 0 | 1 | 0 | **1** | 0 |
|  | 43211-3432-3322-243-233-14-14 | 7 | 0 | 0 | 0 | **1** | 0 |
|  |  | 5-7 | 1 | 3 | 4 | **9** | 0 |
|  |  |  |  |  |  |  |  |
| f4c | 3332-3332-3332-3332-233-233 | 6 | 0 | 0 | 0 | **1** | 0 |
|  | 3322-3322-233-232-232-222 | 6 | 0 | 1 | 0 | **0** | 0 |
|  | 44222-44222-244-244-242-242-222 | 6 | 0 | 0 | 0 | **1** | 0 |
|  | 3332-3332-3221-233-233-233-13 | 7 | 0 | 0 | 0 | **1** | 0 |
|  | 43332-3433-3432-3432-243-231-12 | 7 | 0 | 2 | 0 | **0** | 0 |
|  |  | 6-7 | 0 | 3 | 0 | **3** | 0 |
|  |  |  |  |  |  |  |  |
| f5 | 43322-3432-3432-243-243 | 5 | 5 | 1 | 0 | **0** | 0 |
|  | 3333-3333-3332-3332-233 | 5 | 0 | 1 | 0 | **0** | 0 |
|  | 44332-44332-3442-3442-243-243 | 6 | 2 | 1 | 0 | **0** | 0 |
|  | 43332-3433-3432-3431-243-13 | 6 | 2 | 1 | 1 | **0** | 0 |
|  | 533221-3532-3532-253-253-15 | 6 | 0 | 0 | 1 | **0** | 0 |
|  | 43322-3432-3432-242-242-233 | 6 | 0 | 0 | 0 | **0** | 0 |
|  | 44332-44331-3442-3441-243-14-13 | 7 | 0 | 0 | 1 | **1** | 0 |
|  | 44422-44421-44421-244-244-14-14 | 7 | 0 | 0 | 0 | **1** | 0 |
|  | 44322-44322-3442-244-243-241-12 | 7 | 0 | 1 | 1 | **0** | 0 |
|  | 43332-3432-3432-3421-243-233-13 | 7 | 0 | 0 | 0 | **1** | 0 |
|  |  | 5-7 | 9 | 5 | 4 | **3** | 0 |
|  |  |  |  |  |  |  |  |
| f6a | 42222-242-242-242-242 | 5 | 1 | 1 | 2 | **1** | 0 |
|  | 43222-3421-243-242-242-13 | 6 | 0 | 2 | 2 | **3** | 1 |
|  | 522221-252-252-252-252-15 | 6 | 0 | 0 | 0 | **1** | 1 |
|  | 43322-3421-3421-243-243-13-13 | 7 | 0 | 3 | 1 | **4** | 1 |
|  | 43222-3422-243-242-242-231-12 | 7 | 0 | 1 | 0 | **0** | 0 |
|  | 44222-44211-244-242-242-14-14 | 7 | 0 | 0 | 1 | **0** | 0 |
|  | 532221-3521-253-252-252-15-13 | 7 | 0 | 0 | 0 | **0** | 0 |
|  |  | 5-7 | 1 | 7 | 6 | **9** | 3 |
|  |  |  |  |  |  |  |  |
| f6b | 3322-3322-232-232-232-232 | 6 | 0 | 2 | 0 | **1** | 0 |
|  |  |  |  |  |  |  |  |
| f7 | 42222-242-242-242-242-222-222 | 7 | 0 | 0 | 0 | **1** | 0 |
|  |  |  |  |  |  |  |  |
| f8a | 43331-3433-3433-3433-14 | 5 | 1 | 1 | 0 | **0** | 0 |
|  | 43332-3433-3433-3433-241-12 | 6 | 0 | 0 | 1 | **1** | 0 |
|  |  | 5-6 | 1 | 1 | 1 | **1** | 0 |
|  |  |  |  |  |  |  |  |
| f8b | 43321-3432-3432-3332-243-233-14 | 7 | 0 | 0 | 1 | **1** | 0 |
|  | 44432-44332-44322-3443-3443-244-244 | 7 | 0 | 0 | 0 | **1** | 0 |
|  | 43221-3431-3322-243-243-14-13 | 7 | 0 | 0 | 0 | **1** | 0 |
|  |  | 7 | 0 | 0 | 1 | **3** | 0 |
|  |  |  |  |  |  |  |  |
| f8c | 42221-3222-243-243-243-14 | 6 | 0 | 0 | 0 | **1** | 0 |
|  | 44332-44332-3441-3441-244-13-13 | 7 | 0 | 1 | 0 | **1** | 0 |
|  | 43311-3422-3422-233-233-14-14 | 7 | 0 | 0 | 0 | **1** | 0 |
|  | 3333-3333-3333-3333-3332-231-12 | 7 | 0 | 0 | 0 | **1** | 0 |
|  |  | 6-7 | 0 | 1 | 0 | **4** | 0 |
|  |  |  |  |  |  |  |  |
