# Supplementary material for: Mapping the distribution of packing topologies within protein interiors shows predominant preference for specific packing motifs
Source: BMC Bioinformatics. 2011 May 24;12:195. doi: 10.1186/1471-2105-12-195 (PMC3123238; doi:10.1186/1471-2105-12-195)
Supplement: Additional file 13 — Table S6. Triplet cliques constituted of hydrophobic residues exhibit preferences in their amino acid composition. Frequency distributions of triplet clique compositions in categories (a) C1 (all three residues different), (b) C2 (two residues identical) and (c) C3 (all three identical) are tabulated respectively. [file 1471-2105-12-195-S13.DOC]

**Table S6:**

(a)

| **Composition** | **Frequency** | **Composition** | **Frequency** |
| --- | --- | --- | --- |
| ILE-LEU-VAL | 322 | TRP-PHE-VAL | 25 |
| PHE-ILE-LEU | 276 | PHE-VAL-ALA | 22 |
| PHE-LEU-VAL | 246 | TRP-TYR-LEU | 21 |
| TYR-ILE-LEU | 151 | TRP-ILE-VAL | 21 |
| PHE-ILE-VAL | 150 | TRP-TYR-PHE | 18 |
| TYR-PHE-LEU | 117 | TRP-TYR-ILE | 12 |
| TYR-LEU-VAL | 98 | TRP-TYR-VAL | 10 |
| TYR-PHE-ILE | 85 | PHE-ILE-ALA | 9 |
| TYR-PHE-VAL | 77 | TYR-LEU-ALA | 9 |
| TYR-ILE-VAL | 69 | TYR-PHE-ALA | 8 |
| TRP-PHE-LEU | 56 | TYR-VAL-ALA | 6 |
| LEU-VAL-ALA | 50 | TRP-VAL-ALA | 5 |
| TRP-ILE-LEU | 46 | TYR-ILE-ALA | 4 |
| TRP-LEU-VAL | 41 | TRP-LEU-ALA | 4 |
| TRP-PHE-ILE | 33 | TRP-PHE-ALA | 3 |
| ILE-LEU-ALA | 32 | TRP-ILE-ALA | 3 |
| ILE-VAL-ALA | 30 |  |  |
| PHE-LEU-ALA | 27 | TOTAL | 2086 |

**(b)**

| **Composition** | **Frequency** | **Composition** | **Frequency** |
| --- | --- | --- | --- |
| ILE-LEU-LEU | 291 | LEU-TYR-TYR | 20 |
| VAL-LEU-LEU | 268 | TRP-PHE-PHE | 20 |
| PHE-LEU-LEU | 237 | TRP-ILE-ILE | 19 |
| LEU-ILE-ILE | 187 | VAL-ALA-ALA | 17 |
| LEU-PHE-PHE | 162 | ILE-TYR-TYR | 13 |
| VAL-ILE-ILE | 134 | VAL-TYR-TYR | 12 |
| LEU-VAL-VAL | 128 | LEU-TRP-TRP | 11 |
| ILE-VAL-VAL | 119 | ALA-ILE-ILE | 11 |
| ILE-PHE-PHE | 105 | LEU-ALA-ALA | 9 |
| TYR-LEU-LEU | 104 | ILE-ALA-ALA | 8 |
| PHE-ILE-ILE | 94 | PHE-TRP-TRP | 6 |
| PHE-VAL-VAL | 88 | TRP-VAL-VAL | 6 |
| VAL-PHE-PHE | 67 | ILE-TRP-TRP | 5 |
| TYR-PHE-PHE | 53 | TRP-TYR-TYR | 4 |
| TRP-LEU-LEU | 47 | ALA-PHE-PHE | 3 |
| TYR-ILE-ILE | 46 | VAL-TRP-TRP | 3 |
| TYR-VAL-VAL | 35 | TYR-TRP-TRP | 3 |
| ALA-VAL-VAL | 31 | TRP-ALA-ALA | 1 |
| PHE-TYR-TYR | 29 |  |  |
| ALA-LEU-LEU | 28 | TOTAL | 2434 |

(c)

| **Composition** | **Frequency** | **Composition** | **Frequency** |
| --- | --- | --- | --- |
| LEU-LEU-LEU | 202 | ALA-ALA-ALA | 4 |
| ILE-ILE-ILE | 70 | TRP-TRP-TRP | 2 |
| PHE-PHE-PHE | 43 | TYR-TYR-TYR | 1 |
| VAL-VAL-VAL | 42 | TOTAL | 364 |
