# Supplementary material for: Mapping the distribution of packing topologies within protein interiors shows predominant preference for specific packing motifs
Source: BMC Bioinformatics. 2011 May 24;12:195. doi: 10.1186/1471-2105-12-195 (PMC3123238; doi:10.1186/1471-2105-12-195)
Supplement: Additional file 15 — Table S7: Links constituting the subset {Lcyp}: {Lcyp} represents the subset of links in the surface contact network of 2HAQ which is highly conserved among its close homologues defining the (cyclophilin-like) fold specific subgraph. Sec str represents the location of these links with respect to secondary structural elements and lp stands for the fraction of times the link was found in the corresponding subgraph of the 17 other homologues, used to define {Lcyp}. Two nodes in the same row are connected by a link whose surface complementarity (Sm) and overlap (Ov) are tabulated. In column 2 and 5, Sx, Hy, Lxy and Txy represents Strand x, Helix y, Loop and Turn connecting secondary structural element x and y respectively. [file 1471-2105-12-195-S15.DOC]

**Table S7:**

| **Node1** | | | **Node2** | | | **Link strength** | | |
| --- | --- | --- | --- | --- | --- | --- | --- | --- |
| **Residue** | **Sec str** | **burial** | **Residue** | **Sec str** | **burial** | **Sm** | **Ov** | **lp** |
| 31-PHE | S1 | 0.00 | 181-ILE | S12 | 0.00 | 0.686 | 0.201 | 1.00 |
| 43-ILE | S2 | 0.00 | 154-VAL | S9 | 0.11 | 0.521 | 0.112 | 1.00 |
| 47-LEU | S2 | 0.00 | 59-PHE | H3 | 0.00 | 0.579 | 0.114 | 1.00 |
| 55-THR | H3 | 0.00 | 122-MET | S6 | 0.00 | 0.584 | 0.151 | 1.00 |
| 55-THR | H3 | 0.00 | 149-VAL | T89 | 0.00 | 0.541 | 0.147 | 1.00 |
| 55-THR | H3 | 0.00 | 151-PHE | S9 | 0.00 | 0.675 | 0.183 | 1.00 |
| 59-PHE | H3 | 0.00 | 151-PHE | S9 | 0.00 | 0.613 | 0.162 | 1.00 |
| 62-LEU | H3 | 0.00 | 71-TYR | L34 | 0.00 | 0.708 | 0.162 | 1.00 |
| 106-PHE | L56 | 0.05 | 130-ASN | L67 | 0.00 | 0.7 | 0.15 | 1.00 |
| 115-HIS | L56 | 0.07 | 144-LEU | H8 | 0.09 | 0.708 | 0.128 | 1.00 |
| 115-HIS | L56 | 0.07 | 150-VAL | S9 | 0.00 | 0.679 | 0.128 | 1.00 |
| 120-LEU | S6 | 0.00 | 151-PHE | S9 | 0.00 | 0.514 | 0.137 | 1.00 |
| 122-MET | S6 | 0.00 | 151-PHE | S9 | 0.00 | 0.605 | 0.115 | 1.00 |
| 31-PHE | S1 | 0.00 | 45-ILE | S2 | 0.00 | 0.655 | 0.109 | 0.941 |
| 59-PHE | H3 | 0.00 | 71-TYR | L34 | 0.00 | 0.566 | 0.136 | 0.941 |
| 83-PHE | T45 | 0.18 | 84-MET | S5 | 0.01 | 0.564 | 0.163 | 0.941 |
| 85-ILE | S5 | 0.00 | 164-ILE | H10 | 0.00 | 0.655 | 0.179 | 0.941 |
| 29-VAL | S1 | 0.00 | 47-LEU | S2 | 0.00 | 0.712 | 0.152 | 0.882 |
| 29-VAL | S1 | 0.00 | 63-CYS | H3 | 0.01 | 0.665 | 0.214 | 0.882 |
| 43-ILE | S2 | 0.00 | 160-VAL | H10 | 0.03 | 0.481 | 0.164 | 0.882 |
| 59-PHE | H3 | 0.00 | 134-PHE | S7 | 0.00 | 0.526 | 0.100 | 0.882 |
| 76-PHE | L34 | 0.00 | 85-ILE | S5 | 0.00 | 0.686 | 0.167 | 0.882 |
| 76-PHE | L34 | 0.00 | 181-ILE | S12 | 0.00 | 0.527 | 0.151 | 0.882 |
| 100-SER | L56 | 0.02 | 106-PHE | L56 | 0.05 | 0.605 | 0.196 | 0.882 |
| 108-ASP | L56 | 0.17 | 149-VAL | T89 | 0.00 | 0.673 | 0.11 | 0.882 |
| 136-ILE | S7 | 0.00 | 161-VAL | H10 | 0.00 | 0.646 | 0.144 | 0.882 |
| 43-ILE | S2 | 0.00 | 161-VAL | H10 | 0.00 | 0.605 | 0.144 | 0.824 |
| 71-TYR | L34 | 0.00 | 134-PHE | S7 | 0.00 | 0.545 | 0.105 | 0.824 |
| 77-HIS | L34 | 0.06 | 86-GLN | S5 | 0.08 | 0.505 | 0.146 | 0.824 |
| 80-ILE | S4 | 0.22 | 84-MET | S5 | 0.01 | 0.532 | 0.156 | 0.824 |
| 86-GLN | S5 | 0.08 | 123-ALA | L67 | 0.08 | 0.612 | 0.145 | 0.824 |
