# Supplementary material for: Mapping the distribution of packing topologies within protein interiors shows predominant preference for specific packing motifs
Source: BMC Bioinformatics. 2011 May 24;12:195. doi: 10.1186/1471-2105-12-195 (PMC3123238; doi:10.1186/1471-2105-12-195)
Supplement: Additional file 16 — Table S8. Specific geometry more clearly manifested by surfaces than point atoms. χ2 of tilt angles (θ1t, θ2t, θ3t) and swivel angles (φ1s, φ2s, φ3s) for triplet compositions for (a) ASCN (b) APCN. 1, 2, 3 corresponds to the same sequence of residues given in the table e.g., ILE → 1, LEU → 2, VAL → 3 for the first entry of (a). χ20.05 for three-bin and six-bin models are 5.991 and 11.071, respectively. Compositions which have a predicted frequency of less than 5 for any particular angular bin assuming a random distribution are marked with an asterisk (*). This minimal number (of data points) is 37 for a three-bin and 74 for a six-bin model for tilt (θt) angles and 30 for a six-bin model for swivel (φs) angles. Only those compositions have been given whose frequencies are greater than equal to 25. [file 1471-2105-12-195-S16.DOC]

**Table S8.**

**(a)**

| **Composition** | | | **Frequency** | **χ^2^(θ1_t_)** | **χ^2^(θ2_t_)** | **χ^2^(θ3_t_)** | **χ^2^(φ1_s_)** | **χ^2^(φ2_s_)** | **χ^2^(φ3_s_)** |
| --- | --- | --- | --- | --- | --- | --- | --- | --- | --- |
| ILE | LEU | VAL | 322 | 8.0 | 22.7 | 5.6 | 3.6 | 5.8 | 2.5 |
| ILE | LEU | LEU | 291 | 17.1 | 14.7 | 36.6 | 2.7 | 7.5 | 4.4 |
| PHE | ILE | LEU | 276 | 48.5 | 11.2 | 35.8 | 15.3 | 14.5 | 5.8 |
| VAL | LEU | LEU | 268 | 8.1 | 15.7 | 15.0 | 9.3 | 5.1 | 17.9 |
| PHE | LEU | VAL | 246 | 60.0 | 20.6 | 5.2 | 14.0 | 8.5 | 6.8 |
| PHE | LEU | LEU | 237 | 72.1 | 25.5 | 13.5 | 13.9 | 20.8 | 3.8 |
| LEU | LEU | LEU | 202 | 18.7 | 32.7 | 7.2 | 4.9 | 5.0 | 24.2 |
| LEU | ILE | ILE | 187 | 17.6 | 18.2 | 13.1 | 5.4 | 5.8 | 6.5 |
| LEU | PHE | PHE | 162 | 9.6 | 32.1 | 4.8 | 4.7 | 4.9 | 14.3 |
| TYR | ILE | LEU | 151 | 39.0 | 2.9 | 7.3 | 20.5 | 16.0 | 10.0 |
| PHE | ILE | VAL | 150 | 37.7 | 9.3 | 16.7 | 19.4 | 4.9 | 19.5 |
| VAL | ILE | ILE | 134 | 15.6 | 10.6 | 9.4 | 7.3 | 4.3 | 2.3 |
| LEU | VAL | VAL | 128 | 10.5 | 4.5 | 4.3 | 9.5 | 0.3 | 5.6 |
| ILE | VAL | VAL | 119 | 18.1 | 8.5 | 11.1 | 4.6 | 20.8 | 2.1 |
| TYR | PHE | LEU | 117 | 44.6 | 6.2 | 2.8 | 18.5 | 5.7 | 10.6 |
| ILE | PHE | PHE | 105 | 3.9 | 1.7 | 19.4 | 2.4 | 6.1 | 3.1 |
| TYR | LEU | LEU | 104 | 46.7 | 15.2 | 8.6 | 6.4 | 5.3 | 16.4 |
| TYR | LEU | VAL | 98 | 35.5 | 8.7 | 9.5 | 7.4 | 3.1 | 6.6 |
| PHE | ILE | ILE | 94 | 17.2 | 2.5 | 9.2 | 3.3 | 2.0 | 5.7 |
| PHE | VAL | VAL | 88 | 21.6 | 24.9 | 5.1 | 14.1 | 4.2 | 4.5 |
| TYR | PHE | ILE | 85 | 7.7 | 4.0 | 10.0 | 9.4 | 8.3 | 3.3 |
| TYR | PHE | VAL | 77 | 20.3 | 3.0 | 4.0 | 9.0 | 10.2 | 2.0 |
| ILE | ILE | ILE | 70 | 4.0* | 11.3* | 14.7* | 9.5 | 7.3 | 5.6 |
| TYR | ILE | VAL | 69 | 23.4 | 2.5 | 3.8 | 4.5 | 2.0 | 4.7 |
| VAL | PHE | PHE | 67 | 1.7* | 6.6* | 11.2* | 12.1 | 2.9 | 3.8 |
| TRP | PHE | LEU | 56 | 19.1* | 7.2* | 9.6* | 10.6 | 0.6 | 11.7 |
| TYR | PHE | PHE | 53 | 13.3 | 0.9 | 1.8 | 6.9 | 14.8 | 4.2 |
| LEU | VAL | ALA | 50 | 10.3* | 2.9* | - | 0.9 | 3.8 | - |
| TRP | LEU | LEU | 47 | 29.5* | 4.0* | 5.1* | 3.9 | 17.7 | 2.9 |
| TRP | ILE | LEU | 46 | 11.6* | 4.3* | 11.5* | 3.3 | 12.2 | 4.9 |
| TYR | ILE | ILE | 46 | 5.4 | 11.9 | 1.6 | 7.48 | 8.8 | 9.0 |
| PHE | PHE | PHE | 43 | 7.0 | 2.5 | 12.3 | 7.9 | 4.3 | 9.3 |
| VAL | VAL | VAL | 42 | 8.5* | 18.2* | 9.2* | 10.3 | 3.4 | 6.6 |
| TRP | LEU | VAL | 41 | 23.9* | 5.3* | 3.0* | 6.3 | 6.3 | 3.9 |
| TYR | VAL | VAL | 35 | 15.2* | 3.5* | 2.8* | 11.5 | 3.2 | 2.9 |
| TRP | PHE | ILE | 33 | 12.4* | 10.2* | 9.0* | 5.0 | 1.0 | 13.4 |
| ILE | LEU | ALA | 32 | 2.6* | 2.6* | - | 3.3 | 2.5 | - |
| ILE | VAL | ALA | 30 | 5.3* | 8.6* | - | 10.0 | 3.2 | - |
| PHE | TYR | TYR | 29 | 1.3* | 15.6* | 2.6* | 3.5* | 8.5* | 8.9* |
| PHE | LEU | ALA | 27 | 6.6* | 12.4* | - | 14.6* | 3.9* | - |
| TRP | PHE | VAL | 25 | 10.1* | 4.7* | 2.7* | 3.6* | 8.4* | 12.2* |

**(b)**

| **Composition** | | | **Frequency** | **χ^2^(θ1_t_)** | **χ^2^(θ2_t_)** | **χ^2^(θ3_t_)** | **χ^2^(φ1_s_)** | **χ^2^(φ2_s_)** | **χ^2^(φ3_s_)** |
| --- | --- | --- | --- | --- | --- | --- | --- | --- | --- |
| PHE | ILE | LEU | 67 | 19.1 | 9.0 | 6.0 | 7.3 | 13.2 | 8.1 |
| TYR | PHE | LEU | 66 | 27.5 | 2.4 | 8.6 | 13.5 | 12.3 | 10.2 |
| TYR | PHE | ILE | 65 | 8.6 | 10.5 | 3.3 | 7.3 | 3.8 | 23.3 |
| TYR | PHE | VAL | 56 | 14.0 | 2.4 | 7.7 | 14.9 | 29.9 | 10.3 |
| TYR | PHE | PHE | 51 | 12.7 | 4.9 | 0.2 | 5.9 | 8.8 | 16.4 |
| TRP | PHE | LEU | 50 | 15.8* | 1.4* | 8.7* | 12.7 | 4.1 | 18.3 |
| LEU | PHE | PHE | 49 | 7.7* | 12.7* | 2.0* | 36.4 | 6.2 | 6.2 |
| PHE | LEU | VAL | 43 | 10.8 | 0.6 | 6.8 | 15.1 | 6.4 | 6.9 |
| ILE | PHE | PHE | 42 | 8.3* | 1.5* | 4.7* | 39.6 | 12.1 | 7.0 |
| TYR | ILE | LEU | 41 | 1.2 | 1.2 | 9.1 | 16.2 | 8.2 | 10.0 |
| PHE | TYR | TYR | 40 | 7.0 | 2.4 | 12.1 | 5.6 | 4.7 | 4.9 |
| TYR | LEU | VAL | 39 | 18.7 | 4.7 | 10.4 | 9.9 | 7.0 | 9.2 |
| TYR | ILE | VAL | 36 | 3.4* | 5.2* | 6.7* | 5.8 | 2.1 | 12.0 |
| TRP | PHE | PHE | 34 | 5.5* | 3.2* | 2.0* | 8.5 | 13.7 | 5.6 |
| TYR | LEU | LEU | 34 | 4.2* | 6.7* | 2.0* | 9.6 | 7.2 | 15.4 |
| PHE | ILE | VAL | 32 | 10.4* | 6.2* | 1.3* | 11.3 | 11.5 | 6.9 |
| TRP | TYR | PHE | 32 | 4.6* | 0.1* | 1.2* | 9.9 | 10.9 | 4.5 |
| PHE | LEU | LEU | 29 | 8.0* | 7.0* | 7.0* | 7.4* | 14.2* | 12.7* |
| PHE | PHE | PHE | 29 | 4.6* | 3.4* | 4.2* | 19.2* | 5.9* | 8.2* |
| TRP | PHE | ILE | 28 | 10.8* | 9.2* | 5.5* | 9.2* | 6.6* | 11.0* |
| VAL | PHE | PHE | 28 | 7.2* | 2.7* | 2.3* | 20.4* | 5.3* | 9.9* |
| TRP | TYR | LEU | 27 | 6.3* | 2.0* | 8.0* | 7.5* | 1.8* | 5.6* |
| LEU | TYR | TYR | 27 | 4.7* | 5.2* | 2.5* | 10.4* | 0.8* | 17.3* |
| TRP | PHE | VAL | 26 | 11.4* | 5.1* | 0.9* | 20.4* | 10.3* | 6.6* |
| PHE | ILE | ILE | 25 | 2.1* | 1.4* | 9.9* | 13.8* | 23.0* | 7.9* |
