# Supplementary material for: Mapping the distribution of packing topologies within protein interiors shows predominant preference for specific packing motifs
Source: BMC Bioinformatics. 2011 May 24;12:195. doi: 10.1186/1471-2105-12-195 (PMC3123238; doi:10.1186/1471-2105-12-195)
Supplement: Additional file 17 — Table S9. The Database. Polypeptide chains used in the analysis sorted according to class. The accession numbers (PDB ID), chain identifiers along with resolution in Angstroms and the first and last residue numbers (in case of multidomain proteins) are given in parenthesis. [file 1471-2105-12-195-S17.DOC]

**Table S9:**

**All α :**

**1C02_B (1.80), 1C1K_A (1.45), 1D2T_A (1.90), 1DLJ_A (1.80, 197-294), 1DLW_A (1.54), 1EL4_A (1.73), 1ELK_B (1.50), 1EYH_A (1.56), 1FP2_A (1.40, 8-108), 1FW1_A (1.90, 88-212), 1G5N_A (1.90), 1G8E_A (1.80), 1I8O_A (1.15), 1IAP_A (1.90), 1J0P_A (0.91), 1JKV_A (1.39), 1JMW_A (1.90), 1K3Y_A (1.30, 81-222), 1KR7_A (1.50), 1LJ9_A (1.60), 1M48_B (1.95), 1M70_C (1.25), 1MOJ_A (1.90), 1MQV_B (1.78), 1MXR_B (1.42), 1MZ4_A (1.80), 1OOH_B (1.25), 1OWL_A (1.80, 205-475), 1OXJ_A (1.80), 1PUO_B (1.85), 1Q08_A (1.90), 1Q5Z_A (1.80), 1QYZ_A (1.40), 1R0D_I (1.90), 1R1T_B (1.70), 1R7J_A (1.47), 1S29_A (1.60), 1S2X_A (1.90), 1SBX_A (1.65), 1SG6_B (1.70, 185-392), 1SZH_A (1.50), 1T0F_A (1.85, 169-268), 1TU9_A (1.20), 1U2W_B (1.90), 1U84_A (1.60), 1U9P_A (1.90), 1UCR_B (1.20), 1VDK_B (1.80), 1XG0_D (0.97), 1Y02_A (1.80), 1Y0U_B (1.60), 1YQS_A (1.05, 61-270), 1YT3_A (1.60, 194-294), 1ZEL_B (1.93, 1-82), 1ZKR_A (1.64), 1TFJ_A (1.61), 1WXC_A (1.20), 1EZJ_A (1.90), 1WPA_A (1.50), 1R5Z_B (1.95), 1U60_C (1.61, 61-201), 1U7L_A (1.75, 50-169, 263-324), 1UJN_A (1.80, 162-324), 1XFI_A (1.70, 1-119), 2A61_A (1.80), 2AIB_A (1.10), 2AO9_A (1.90), 2AP3_A (1.60), 2CWL_A (1.65), 2FBQ_A (1.80), 2FD5_A (1.70), 2FMM_E (1.80), 2FP1_A (1.55), 2G62_A (1.00), 2GA2_A (1.95, 375-448), 2GAU_A (1.90, 10-151), 2GEN_A (1.70), 2GYQ_A (1.40), 2GZJ_F (1.60, 3-85), 2HEK_B (2.00), 2I1Q_A (1.90, 5-64), 2I5U_A (1.50), 2INC_A (1.85), 2O38_A (1.83), 2O70_F (1.80), 2OEB_A (1.66), 2OOC_B (1.52), 2PQR_B (1.88), 2R8U_B (1.35), 2B8I_A (1.80), 2CWC_A (1.65), 2D0W_B (1.98), 2D48_A (1.65), 2D4X_A (1.90), 2DH4_A (1.98), 2DPO_A (1.70), 2EH3_A (1.55), 2ERB_A (1.50), 2EXV_A (1.86), 2F2B_A (1.68), 2FDV_B (1.65), 2FU4_A (1.80), 2GRH_A (1.50), 2GXG_A (1.45), 2H8O_A (1.60), 2HRA_A (1.90), 2I2O_A (1.92), 2I3F_B (1.38), 2IC6_B (1.15), 2IEQ_B (1.75), 2IGP_A (1.80), 2IMI_B (1.00), 2NX0_A (0.95), 2NZ7_B (1.00), 2O37_A (1.25), 2OIF_G (1.80), 2OQG_C (1.54), 2P0B_A (1.74), 2P0N_A (1.41), 2P58_C (1.80), 2POS_C (1.60), 2PVQ_A (1.80), 2QRW_K (1.93), 2QSA_A (1.68), 2RKN_A (1.60), 2ZCA_B (1.80), 2BA2_C (1.80), 2CZ1_B (1.39, 1-111), 3BGE_B (1.85), 3DHZ_B (1.63), 3B4Q_B (1.55), 3B9W_A (1.30), 3BG2_B (1.95), 3BJD_C (1.85), 3BS7_B (1.90), 3BT5_A (1.35), 3C1D_B (1.00), 3CHM_A (1.50), 3DT5_A (1.94), 3ECH_B (1.80), 3FES_D (1.00), 2E1N_B (1.80), 3E6S_D (1.95).**

**All β :**

**1D2S_A (1.55), 1DJR_H (1.30), 1DMH_B (1.70), 1DQG_A (1.70), 1DQI_B (1.70), 1DTO_A (1.90), 1EZG_B (1.40), 1F41_B (1.30), 1F60_A (1.67, 241-441), 1F7D_A (1.40), 1G8L_B (1.95, 7-177), 1HM9_B (1.75, 252-447), 1HYO_B (1.30, 499-618), 1I0R_B (1.50), 1I3H_A (1.20), 1I4U_A (1.15), 1IAZ_B (1.90), 1IBY_B (1.65), 1IG3_A (1.90, 179-263), 1J71_A (1.80), 1JB9_A (1.70, 6-162), 1K12_A (1.90), 1K7I_A (1.59, 259-479), 1KHX_A (1.80), 1KJL_A (1.40), 1KT6_A (1.10), 1KTB_A (1.90, 294-388), 1KZQ_B (1.70), 1LR5_C (1.90), 1LXZ_A (1.25), 1LYQ_B (1.50), 1M1H_A (1.95, 51-131), 1MRZ_B (1.90, 459-589), 1N08_B (1.60), 1N12_A (1.87), 1N68_A (1.70), 1NJH_A (1.70), 1NTV_A (1.50), 1NYK_B (1.31), 1NYW_B (1.60), 1ORU_B (1.80), 1OU8_B (1.60), 1OW1_A (1.80), 1P1M_A (1.50, 1-49, 331-404), 1P3C_A (1.50), 1PU5_B (1.90), 1PXD_A (1.80), 1QB5_E (1.90), 1QUP_A (1.80, 2-73), 1QWR_A (1.80), 1QWX_A (1.50), 1QWY_A (1.30), 1QXM_A (1.70), 1R8O_A (1.83), 1RA0_A (1.12, 4-55, 376-426), 1RG8_B (1.10), 1ROC_A (1.50), 1RQP_C (1.80, 193-298), 1RU4_A (1.60), 1RWI_B (1.80), 1S1D_A (1.60), 1SMX_A (1.80), 1SZN_A (1.54, 315-417), 1T2W_C (1.80), 1T9H_A (1.60, 1-67), 1TH7_L (1.68), 1V8H_A (1.20), 1VM9_A (1.48), 1WD3_A (1.75), 1WLG_B (1.80), 1WLI_B (1.60), 1WS7_A (1.90), 1X8Q_A (0.85), 1XRT_B (1.61, 1-55, 366-422), 1XU1_B (1.90), 1Y0Y_A (1.60, 73-163), 1YFQ_A (1.10), 1YI9_A (1.70), 1YOA_A (1.90), 1YU0_A (1.56, 5-170), 1Z0N_A (1.49), 1Z3T_A (1.70), 1Z8K_B (1.71), 1ZCE_A (1.30), 1ZDS_B (1.55), 1ZJC_A (1.80, 234-415), 1ZPS_B (1.70), 1ZV9_A (1.28), 1R77_B (1.75), 1WZO_B (1.90), 1ZD7_B (1.70), 1ZLD_A (1.65), 1ZVT_B (1.70), 1LSL_A (1.90), 1SE0_A (1.75), 1Z6F_A (1.60, 263-356), 1U7L_A (1.75, 170-262), 2A13_A (1.32), 2A6V_A (1.52), 2AYW_A (0.97), 2CXK_A (1.85), 2CZ1_B (1.39, 112-221), 2DCY_C (1.40), 2DP9_A (1.90), 2ERF_A (1.45), 2ET1_A (1.60), 2G9F_A (1.90), 2GAU_A (1.90, 152-232), 2GH2_A (1.50), 2GUD_A (0.94), 2GUY_A (1.59, 382-476), 2GVV_A (1.73), 2HAL_A (1.35), 2O1A_A (1.60), 2O8L_A (1.50), 2PA7_A (1.50), 2PUZ_B (1.83, 17-79, 381-420), 2AEN_H (1.60), 2AI4_A (1.57), 2ASK_B (1.55), 2AYD_A (1.60), 2BOU_A (1.90), 2D37_A (1.70), 2DUR_A (1.65), 2EI5_A (1.88), 2F6E_A (1.85), 2FIM_B (1.90), 2G6Y_A (1.60), 2GGV_B (1.80), 2HNF_A (1.80), 2HZQ_A (1.80), 2ICC_A (1.20), 2IQY_A (1.40), 2O30_A (1.66), 2OFZ_A (1.17), 2OR7_B (1.50), 2OZI_B (1.15), 2PCX_A (1.54), 2QF4_A (1.20), 2R2A_A (1.82), 2R2C_A (1.80), 2R5O_A (1.30), 2RJ2_A (1.70), 3ETJ_A (1.00, 277-355), 3B4N_A (1.45), 3BOV_A (1.77), 3BU1_A (1.40), 3CBT_A (1.70), 3D0F_A (1.64), 3D9X_C (1.13), 3EC6_A (1.60), 3EDS_A (1.76), 3F2Z_A (1.30), 3F5R_A (1.70).**

**α|β :**

**1C7N_C (1.90), 1CXQ_A (1.02), 1D0I_H (1.80), 1D4O_A (1.21), 1DJE_A (1.71), 1DLJ_A (1.80, 1-196, 295-402), 1DQZ_B (1.50), 1EEH_A (1.90), 1EEJ_B (1.90, 61-216), 1EQC_A (1.85), 1ESJ_B (1.80), 1EU8_A (1.90), 1F60_A (1.67, 2-240), 1F8M_B (1.80), 1FP2_A (1.40, 109-352), 1FW1_A (1.90, 5-87), 1G5T_A (1.80), 1G60_A (1.74), 1G8A_A (1.40), 1G8L_B (1.95, 178-326, 327-409), 1GA6_A (1.00), 1HM9_B (1.75, 2-251), 1I5G_A (1.40), 1I6W_B (1.50), 1I9C_B (1.90), 1IG3_A (1.90, 10-178), 1IM5_A (1.65), 1INL_C (1.50), 1IZC_A (1.70), 1J3A_A (1.60), 1J9B_A (1.26), 1JAK_A (1.75, 151-506), 1JAY_B (1.65), 1JB9_A (1.70, 163-316), 1JJF_A (1.75), 1JKE_C (1.55), 1JX6_A (1.50), 1K3Y_A (1.30, 2-80), 1K4M_B (1.90), 1K7C_A (1.12), 1KTB_A (1.90, 1-293), 1LBV_A (1.80, 142-152), 1LL2_A (1.90), 1LOK_A (1.20), 1LUA_B (1.90), 1LWD_B (1.85), 1LZJ_A (1.32), 1M65_A (1.57), 1MEO_A (1.72), 1MRZ_B (1.90, 302-458), 1N3Y_A (1.65), 1NF9_A (1.50), 1NLF_B (1.95), 1NP6_B (1.90), 1NQU_B (1.75), 1O03_A (1.40), 1O04_G (1.42), 1OOE_B (1.65), 1OOY_B (1.70), 1OWL_A (1.80, 3-204), 1OZN_A (1.52), 1P0K_B (1.90), 1P1M_A (1.50, 50-330), 1P6O_B (1.14), 1Q0R_A (1.45), 1Q74_A (1.70), 1Q7L_A (1.40), 1QWO_A (1.50), 1R7O_A (1.85), 1RA0_A (1.12, 56-375), 1RKU_B (1.47), 1RQP_C (1.80, 8-192), 1SG0_B (1.50), 1SW5_B (1.80), 1SZN_A (1.54, 1-314), 1T0F_A (1.85, 7-168), 1T1V_B (1.60), 1T2D_A (1.10, 1-150), 1T4B_B (1.60, 1-133, 355-367), 1T5B_A (1.40), 1T6T_2 (1.80), 1T9H_A (1.60, 68-298), 1TJY_A (1.30), 1TP9_B (1.62), 1TQ4_A (1.95), 1TVP_A (1.60), 1TZZ_B (1.86, 2146-2392), 1UB3_B (1.40), 1UC7_A (1.90), 1UEH_B (1.73), 1UG6_A (0.99), 1UJP_A (1.34), 1UK8_A (1.60), 1V2X_A (1.50), 1V37_A (1.40), 1V6S_B , (1.50), 1V6T_A (1.70), 1VBK_A (1.90, 176-307), 1VFL_A (1.80), 1WL8_A (1.45), 1WL9_A (1.90, 1-176), 1WN5_B (1.80), 1WO8_A (1.70), 1WOU_A (1.80), 1WPN_A (1.30), 1WZN_B (1.90), 1X7Y_A (1.57), 1X9I_B (1.16), 1XBY_A (1.58), 1XDZ_A (1.60), 1XQ6_B (1.80), 1XRT_B (1.61, 56-365), 1XS5_A (1.85), 1Y0Y_A (1.60, 6-72, 164-351), 1Y1P_A (1.60), 1Y6V_A (1.60), 1Y7L_A (1.55), 1YB6_A (1.54), 1YT3_A (1.60, 1-193), 1Z6N_A (1.50), 1ZJC_A (1.80, 1-233), 1ZL0_A (1.10), 1T2B_B (1.70), 1WX1_B (1.97), 1X13_A (1.90), 1XRO_A (1.80), 1XX1_B (1.75), 1Y1T_A (1.77), 1YD0_A (1.50), 1YJ7_D (1.80), 1YM3_A (1.75), 1YN9_B (1.50), 1YQZ_B (1.54), 1YSQ_A (1.75), 1YZX_B (1.93), 1Z0W_A (1.20), 1ZOI_C (1.60), 1ZR6_A (1.55), 1ZX0_C (1.86), 1ZY7_A (1.70), 1ZZG_A (1.95), 1ZZW_B (1.60), 1DGF_B (1.50, 69-501), 1I2A_A (1.85, 1-56, 57-148, 149-212), 1I2K_A (1.79, 1-109, 110-269), 1SG6_B (1.70, 1-184), 1UJN_A (1.80, 1-161), 1XFI_A (1.70, 120-365), 2A14_A (1.70), 2A1I_A (1.90), 2A9S_A (1.75), 2ACF_C (1.40), 2AEE_A (1.95), 2AEU_A (1.70), 2AFW_B (1.56), 2AP1_A (1.90), 2AVD_B (1.70), 2AXO_A (1.80), 2B82_A (1.25), 2CYG_A (1.45), 2FPR_B (1.70), 2FXU_A (1.35), 2FY6_A (1.90), 2G2C_A (1.50), 2G84_A (1.40), 2GDQ_B (1.80, 119-374), 2GUY_A (1.59, 1-381), 2GZ1_B (1.80, 2-127, 330-357), 2H1C_A (1.80), 2HL6_B (1.55), 2HOR_A (1.60), 2HRC_B (1.70), 2HY5_B (1.72), 2I1Q_A (1.90, 65-322), 2I7N_B (1.90), 2I9I_A (1.80), 2IGT_C (1.89), 2NZX_C (1.90), 2P0S_A (1.60), 2PUZ_B (1.83, 80-380), 2R4Q_A (1.60), 2RBK_A (1.00), 2RDI_A (1.92, 1-238, 239-352), 2A2K_A (1.52), 2AGD_B (1.90), 2ARR_A (1.55), 2B1K_A (1.90), 2B3F_B (1.56), 2B6D_A (1.40), 2DEJ_A (1.50), 2FB9_A (1.90), 2FQX_A (1.70), 2G95_B (1.90), 2GAS_B (1.60), 2GSO_A (1.30), 2H1V_A (1.20), 2HC9_A (1.85), 2HHG_A (1.20), 2HJP_A (1.90), 2HPJ_A (1.70), 2HU9_A (1.78), 2HWW_A (1.80), 2HXU_A (1.80), 2HXW_B (1.60), 2HZL_B (1.40), 2I49_A (1.35), 2I56_C (1.97), 2NWR_A (1.50), 2O0B_A (1.15), 2O0J_A (1.80), 2O7R_A (1.40), 2OUI_B (1.77), 2P51_A (1.40), 2PGE_A (1.60), 2PJM_A (1.78), 2PRB_A (1.80), 2PS1_A (1.75), 2QDX_A (1.55), 2QIP_A (1.48), 2QSI_A (1.80), 2QUL_B (1.79), 2REM_C (1.90), 2RHJ_A (1.76), 2YW3_D (1.67), 2Z1Y_B (1.75), 2Z6R_B (1.50), 2FLA_A (0.95), 2I71_B (1.70, 2-236, 237-377), 3BOE_A (1.40), 3ETJ_A (1.00, 1-78), 3B4U_B (1.20), 3B8B_A (1.70), 3B8X_B (1.70), 3BJE_B (1.44), 3BWY_A (1.30), 3C9H_B (1.90), 3CAW_B (1.87), 3CQ5_B (1.80), 3D2H_A (1.00), 3DAF_A (1.00), 3DSK_A (1.55), 3ED5_A (1.72), 3ES7_B (1.90), 3EUR_A (1.00), 3EVF_A (1.00), 3F67_A (1.74), 3FCX_B (1.00), 3FDX_A (1.00), 2E3D_A (1.95), 2E5F_A (1.35), 3E96_B (1.80).**

**α+β :**

**1C8U_A (1.90), 1DJ0_A (1.50), 1DLG_B (1.90), 1EEJ_B (1.90, 1-60), 1EKG_A (1.80), 1EQ6_A (1.90), 1EW0_A (1.40), 1F1U_A (1.50), 1F46_B (1.50), 1F5M_B (1.90), 1F9Z_B (1.50), 1G1T_A (1.50), 1G2R_A (1.35), 1HYO_B (1.30, 619-917), 1I4J_B (1.80), 1IEE_A (0.94), 1IQZ_A (0.92), 1J1G_A (1.60), 1J1Y_A (1.70), 1J27_A (1.70), 1J3W_B (1.50), 1J98_A (1.20), 1JAK_A (1.75, 8-150), 1JYH_A (1.80), 1JYO_B (1.90), 1K2A_A (1.00), 1K7I_A (1.59, 18-258), 1KJO_A (1.60), 1KQB_C (1.80), 1KUX_A (1.80), 1LG7_A (1.96), 1LN4_A (1.50), 1LO7_A (1.50), 1LQP_B (1.19), 1M1H_A (1.95, 5-50,132-186), 1M4D_A (1.80), 1M4J_B (1.60), 1M5S_A (1.85), 1NE9_A (1.70), 1NWA_A (1.50), 1NWW_B (1.20), 1NWZ_A (0.82), 1OQV_A (1.30), 1OZ9_A (1.89), 1P0H_A (1.60), 1PC4_A (1.65), 1PPV_B (1.70), 1Q4R_A (1.90), 1QUP_A (1.80, 74-222), 1R1M_A (1.90), 1R29_A (1.30), 1R45_A (1.57), 1R4P_A (1.77), 1R8H_C (1.90), 1RC9_A (1.60), 1RKI_A (1.60), 1RWZ_A (1.80), 1RYL_A (1.60), 1S5U_E (1.70), 1SJY_A (1.39), 1SQE_B (1.50), 1T2D_A (1.10, 151-315), 1T4B_B (1.60, 134-354), 1T92_A (1.60), 1TA8_A (1.80), 1TFZ_A (1.80), 1TIQ_A (1.90), 1TKE_A (1.46), 1TP6_A (1.50), 1TR0_I (1.80), 1TU1_A (1.95), 1TUA_A (1.50), 1TUH_A (1.85), 1TUV_A (1.70), 1TZP_A (1.40), 1TZZ_B (1.86, 2005-2145), 1U07_A (1.13), 1U0K_A (1.50), 1UKK_A (1.60), 1UMG_A (1.80), 1VBK_A (1.90, 1-175), 1WJ9_A (1.90), 1WL9_A (1.90, 177-440), 1WRI_A (1.20), 1WWZ_B (1.75), 1XBI_A (1.45), 1XMT_A (1.15), 1XPP_D (1.60), 1XQA_B (1.80), 1XY7_B (1.80), 1Y60_D (1.90), 1YAR_D (1.90), 1YQH_A (1.70), 1YQS_A (1.05, 1-60, 271-347), 1YSR_A (1.78), 1YU0_A (1.56, 171-380), 1YVO_B (1.90), 1Z4R_A (1.74), 1ZDY_A (1.44), 1ZEL_B (1.93, 83-294), 1ZHX_A (1.50), 1ZXU_A (1.70), 1YGT_A (1.70), 1YPY_A (1.51), 1YWM_A (1.86), 1ZHQ_G (1.90), 1ZT3_A (1.80), 1JD5_A (1.90), 1Z6F_A (1.60, 4-262), 1LBV_A (1.80, 1-141), 1U60_C (1.61, 1-60, 202-310), 2A15_A (1.00), 2A6S_B (1.77), 2AAL_A (1.65), 2ACY_A (1.80), 2AZW_A (1.90), 2B06_A (1.40), 2B0V_A (1.55), 2BEI_B (1.84), 2CVE_A (1.60), 2DSY_D (1.90), 2FBL_B (1.90), 2FH1_B (1.55), 2FHZ_A (1.15), 2FL4_A (1.60), 2FRE_A (1.90), 2FVV_A (1.25), 2G3A_A (1.90), 2G64_A (1.80), 2G8O_A (1.30), 2GA2_A (1.95, 110-374, 449-478), 2GDQ_B (1.80, 4-118), 2GEY_A (1.80), 2GIA_G (1.89), 2GMN_B (1.40), 2GU3_A (1.74), 2GZ1_B (1.80, 128-329), 2GZJ_F (1.60, 4-135), 2HIY_C (1.40), 2HNG_A (1.63), 2HQY_A (1.80), 2IF6_A (1.80), 2IMJ_A (1.50), 2NN5_A (1.45), 2NQW_A (1.30), 2NR7_A (1.30), 2NRK_A (1.65), 2NZC_D (1.95), 2P3H_A (1.80), 2P8I_B (1.40), 2PAG_A (1.60), 2PD1_A (1.86), 2PHN_A (1.35), 2PKH_C (1.95), 2PLI_A (1.70), 2PWW_A (1.82), 2R2Z_A (1.20), 2B18_A (1.80), 2BBE_A (1.97), 2DT4_A (1.60), 2DXU_A (1.28), 2EA1_A (1.80), 2F7V_A (1.75), 2FJR_B (1.95), 2FL7_A (1.85), 2G0C_A (1.70), 2GCU_C (1.48), 2GEB_A (1.70), 2GH5_B (1.70), 2GHT_A (1.80), 2GWM_A (1.50), 2H2Z_A (1.60), 2HBT_A (1.60), 2I2Q_A (1.72), 2I7D_A (1.20), 2IG8_B (1.90), 2NTT_B (1.56), 2NWH_A (1.86), 2NYI_A (1.80), 2O28_A (1.80), 2OH5_A (1.98), 2OIX_A (1.80), 2P0W_A (1.90), 2P25_A (1.70), 2P2O_B (1.74), 2PLQ_A (1.90), 2PN0_A (1.70), 2PU3_A (1.50), 2Q0L_B (1.45), 2Q9F_A (1.90), 2QKP_B (1.75), 2QLW_A (1.60), 2QQZ_A (1.92), 2QTS_E (1.90), 2QUD_A (1.60), 2R47_B (1.88), 2R6Z_B (1.80), 2R78_C (1.60), 2RBB_A (1.82), 2Z51_A (1.35), 2FYG_A (1.80), 3BK8_A (1.60), 3ETJ_A (1.00, 79-276), 3B6H_B (1.62), 3B79_A (1.37), 3BB7_A (1.50), 3BCY_A (1.70), 3BIQ_A (1.73), 3BJK_C (1.90), 3BP1_A (1.53), 3BV8_A (1.75), 3BWL_A (1.73), 3C8I_B (1.95), 3CK6_E (1.90), 3COU_A (1.80), 3D01_L (1.70), 3D7J_E (1.45), 3DA4_A (1.70), 3DF8_A (1.65), 3DHA_A (0.95), 3DN7_B (1.80), 2E8G_B (1.00), 2E11_B (1.73), 2E12_A (1.70).**
